# Sustainable Polymer Aerogels: Multiscale Design from Biomass and Thermoset Networks to AI-Guided Materials Discovery

**DOI:** 10.3390/gels12090824

**Published:** 2026-09-08

**Authors:** Trung Chi Duong, Phan Minh Quoc Binh, Dam Thi Thanh Hai, Le Thanh Thanh, Truong Thanh Tuan, Nguyen Thi Phuong Nhung, Nguyen Van Kiet, Nga H. N. Do, Hai M. Duong

**Affiliations:** 1 Refining and Petrochemical Department, Petroleum Faculty, Petrovietnam University, 762 Cach Mang Thang Tam, Ba Ria Ward, Ho Chi Minh City 78117, Vietnam; binhpmq@pvu.edu.vn (P.M.Q.B.); haidtt@pvu.edu.vn (D.T.T.H.); thanhlt@pvu.edu.vn (L.T.T.); tuantt@pvu.edu.vn (T.T.T.); nhungntp@pvu.edu.vn (N.T.P.N.); kietnv@pvu.edu.vn (N.V.K.); 2 Institute for Tropical Technology (VITTEP), 57A Truong Quoc Dung Street, Phu Nhuan Ward, Ho Chi Minh City 70073, Vietnam; ngado.spe@gmail.com; 3 Faculty of Agriculture and Fisheries, Mekong University (MKU), Vinh Long City 85216, Vietnam; haiduong@nus.edu.sg; 4 Department of Mechanical Engineering, National University of Singapore (NUS), Singapore 117575, Singapore

**Keywords:** polymer aerogels, cleaner production, life-cycle assessment, dynamic covalent networks, circular economy, machine-learning-guided design

## Abstract

Polymer aerogels have attracted increasing attention as lightweight porous materials for thermal insulation, separation, adsorption, remediation, and other environmental applications. Their low density and tunable surface chemistry also make them suitable for converting renewable, recycled, and waste-derived feedstocks into value-added materials. However, their overall sustainability remains difficult to assess because most studies focus on material properties, whereas solvent use, drying energy, processing yield, durability, regeneration, and end-of-life pathways are reported less consistently. This review examines sustainable polymer aerogels from the perspectives of cleaner production and waste valorization and focuses on two main features. First, a unified multiscale framework of structure, formation, and performance links network formation mechanisms, pore architecture, and macroscopic behavior across biomass-derived, thermoset, dynamic covalent, hybrid, and recycled polymer aerogels, which are compared in terms of feedstock origin, processing intensity, functional performance, durability, and circularity. Second, structure–property mapping is combined with sustainability-constrained, AI-guided design, with environmental descriptors treated as optimization objectives from the outset rather than as post hoc justifications. Particular attention is given to waste and secondary resources, including agricultural residues, textile waste, paper waste, recycled poly(ethylene terephthalate), and end-of-life tire fibers. The review also discusses how life-cycle assessment, service-based functional units, and minimum reporting standards can help assess whether sustainability claims are supported by measurable environmental benefits. Several recurring limitations emerge from the literature: sustainability is often discussed only qualitatively, processing data are insufficient to support robust life-cycle assessments, solvent exchange and drying remain major environmental hotspots, and circularity claims frequently conflate bio-based content, biodegradability, recyclability, and reusability. Finally, the review discusses how data-driven tools, including literature mining, machine learning, and multi-objective optimization, can support polymer-aerogel design when environmental descriptors are included from the beginning of materials development. The review also proposes a reporting and design roadmap for future work toward polymer aerogels that combine useful performance with lower resource intensity and credible end-of-life value retention.

## 1. Introduction

Polymer aerogels combine ultralow density, hierarchical porosity, and tunable surface chemistry, enabling demanding functions in thermal insulation, separation, adsorption, catalysis, sensing, and energy storage with a minimal amount of solid matter. Two main precursor families dominate their development. Biomass-derived precursors—including cellulose, nanocellulose, chitosan, chitin, alginate, starch, lignin, gelatin, and silk fibroin, together with agricultural, forestry, and marine residues—offer renewable origin, abundant functional groups, mild aqueous processing, and clear waste-valorization potential, although they typically suffer from moisture sensitivity, low wet strength, limited thermal stability, and batch-to-batch variability. Thermoset precursors—phenolic, resorcinol–formaldehyde, polyimide, polyurethane, polyurea, and epoxy-based systems—provide high thermal stability, chemical resistance, and mechanical robustness, but their permanent covalent crosslinks hinder repair, reshaping, and closed-loop recycling. Recent work on dynamic covalent networks and vitrimers, using transesterification, imine, boronic ester, disulfide, vinylogous urethane, or siloxane exchange, offers a way to retain thermoset-like performance while enabling reprocessing, self-healing, and chemical recycling, helping bridge the long-standing trade-off between high-performance and circular polymer aerogels.

The macroscopic performance of polymer aerogels emerges from structures organized across multiple length scales, from molecular crosslinking and nanoscale skeleton topology to mesoscale pore architecture and macroscopic shape stability. Achieving such hierarchical control requires regulating coupled non-equilibrium processes during sol–gel transition, phase separation, aging, solvent exchange, and drying. Reaction–diffusion competition during gelation, the pathway of phase separation (spinodal decomposition vs. nucleation–growth), kinetic arrest, and curvature-driven coarsening (Ostwald ripening) collectively dictate network connectivity, fractal-like topology, and pore-size distribution. During drying, capillary stresses associated with Laplace pressure can cause shrinkage, collapse, or cracking unless balanced by mechanical resistance through supercritical, freeze-, or carefully managed ambient-pressure routes. Because each step simultaneously determines the resulting structure and the associated processing intensity—solvent volume, drying energy, time, and yield—multi-scale design must be approached as an integrated structure–processing problem rather than as a sequence of independently optimized steps.

The high-dimensional coupling among precursor chemistry, gelation route, aging conditions, drying method, pore architecture, and target performance makes purely empirical, trial-and-error optimization slow, costly, and poorly reproducible. Data-driven and AI-guided strategies—including literature mining and natural language processing, surrogate property prediction, Bayesian optimization, active learning, generative models, and autonomous self-driving laboratories—can substantially accelerate the navigation of this design space. This design space provides a basis for materials optimization. Combined with multiscale computational modeling, these tools allow synthesis conditions to be steered toward target structures and properties while requiring substantially fewer experimental iterations than conventional one-factor-at-a-time screening. For sustainable polymer aerogels, however, predictive efficiency is meaningful only when environmental descriptors—solvent intensity, drying energy, renewable or recycled content, hazard profile, regeneration cycles, and end-of-life pathway—are embedded as explicit design variables alongside performance, rather than appended as post hoc justifications after a candidate has already been selected.

Despite rapid progress, four critical gaps limit the translation of polymer aerogels into cleaner-production technologies: (i) sustainability is typically discussed qualitatively and only at the end of narratives organized by material class, hindering service-normalized comparison; (ii) processing histories, shrinkage, density, geometry, and durability protocols are inconsistently reported, weakening both life-cycle assessment and machine-learning model development; (iii) cleaner-processing strategies remain less developed than chemical innovations, with solvent volumes, drying energy, recovery ratios, and yields rarely quantified; and (iv) circularity claims often conflate bio-based content, biodegradability, recyclability, and reusability, which in fact represent distinct end-of-life pathways. Against this background, the present review brings together three design aspects that are often treated separately—feedstock sustainability (biomass-derived, waste-derived, and recycled precursors), network circularity (dynamic covalent thermosets and vitrimer-like aerogels), and data-driven sustainability optimization (AI- and modeling-guided discovery anchored on environmental descriptors). We compare biomass, conventional thermoset, dynamic covalent, and hybrid aerogel families through conceptual, literature-informed structure–property–performance maps, evaluate applications by service-based functional units rather than isolated record properties, and propose a roadmap toward “born-sustainable” aerogels in which renewable or recycled feedstocks, dynamic networks, and validated end-of-life pathways are co-designed at the molecular stage rather than added downstream.

The key cleaner-production design dimensions and corresponding sustainability metrics considered throughout this review are summarized in Table 1.

**Table 1:** Cleaner-production design dimensions and corresponding metrics for sustainable polymer aerogels.

**Design Dimension**	**Examples of Metrics**	**Cleaner-Production Relevance**	**Discussed in**
Feedstock circularity	Bio-based fraction; recycled content; waste-stream origin; precursor yield	Distinguishes renewable origin from verified resource efficiency	Introduction; Section 3 and Section 9
Processing intensity	Solvent volume; solvent recovery ratio; drying time; drying energy; carbonization yield	Identifies hotspots and cleaner processing opportunities	Introduction; Section 7, Section 9 and Section 10
Functional performance	Thermal resistance over lifetime; adsorption capacity retained over cycles; strength-to-density ratio	Moves beyond isolated record properties toward service-based comparison	Section 5, Section 8 and Section 9
Durability and regeneration	Cycles to 80% performance retention; mass loss per cycle; moisture stability; creep resistance	Determines whether high performance persists during real use	Section 8, Section 9 and Section 10
End-of-life value retention	Repairability; reprocessability; monomer recovery; biodegradation route; downcycling risk	Clarifies circular economy claims and avoids vague sustainability language	Section 4, Section 7, Section 9 and Section 10

## 2. Fundamentals of Polymer Aerogel Design

### 2.1. Universal Mechanistic Framework of Aerogel Formation

Aerogel formation can be described as a nonequilibrium transition from a fluid-like precursor sol to a mechanically coherent porous network. Unlike equilibrium phase transitions, which are primarily governed by free-energy minimization, polymer aerogel formation proceeds through dynamic pathways in which molecular association, cluster growth, phase separation, and kinetic arrest occur concurrently or sequentially. As connectivity develops, finite clusters merge at the gel point into a system-spanning network that enables stress transfer and preserves a three-dimensional porous architecture. This transition can be rationalized using percolation-based concepts, although real aerogel systems often deviate from ideal percolation behavior because of structural heterogeneity, concentration effects, interaction potentials, and processing history [1,2].

Kinetic arrest is central to aerogel formation. During gelation, the progressive reduction in molecular, colloidal, or particulate mobility limits structural relaxation and traps transient configurations before they can evolve toward equilibrium. Aerogel architecture should therefore be regarded as a kinetically encoded state rather than as a uniquely defined equilibrium morphology, as illustrated by colloidal systems in which arrested phase separation yields stress-free gels [3]. Similar precursor compositions can generate distinct network topologies when aggregation rate, diffusion, quench depth, catalyst concentration, solvent quality, or drying route is varied, highlighting the role of processing history in morphology selection [4]. This pathway-dependent interpretation is further supported by polymer networks in which percolation induces gel–gel phase separation [5] and by hydrogels in which arrested phase separation enhances strength while reducing hysteresis [6].

This pathway-dependent view is especially important for sustainable polymer aerogels because renewable and recycled precursors often introduce additional heterogeneity into gel-network formation [7]. In biomass-derived aerogels, pathway dependence is shaped by biopolymer interactions, crystallinity, feedstock variability, and moisture-sensitive network formation, whereas in thermoset, hybrid, and recycled aerogels, it is expressed through curing kinetics, crosslink fixation, phase compatibility, filler–binder interactions, solvent management, and drying-induced pore preservation [8]. Waste-derived aerogels—from recycled tire rubber and tire textile fibers to magnesium, aluminum, and fly-ash-based inorganic systems—provide a representative illustration of this principle, demonstrating that waste-feedstock morphology, network formation, and drying-induced structural retention jointly determine porous architecture and multifunctional performance [9,10].

Porous structure formation is further governed by the dynamic coupling between gelation and phase separation. Gelation progressively fixes connectivity through physical association or covalent bonding, while phase separation generates compositional or density fluctuations that template the emerging pore morphology [10,11]. The final architecture is therefore governed by the balance between demixing and network fixation, which can be approximated by the relative timescales of reaction and diffusion [3,4]. When network formation outpaces diffusive demixing, phase separation may be suppressed or arrested at an early stage, yielding finer and more homogeneous porous structures. Conversely, when demixing precedes gelation, phase-separation kinetics dominate before connectivity is fixed, leading to larger domains and more heterogeneous pore networks. Depending on interaction strength and quench conditions, demixing may proceed through spinodal decomposition, favoring bicontinuous morphologies, or through nucleation–growth, producing more discrete domains [4]. Many aerogel-forming systems therefore occupy intermediate regimes in which gelation interrupts ongoing phase separation and preserves a metastable porous architecture rather than allowing full equilibration, as reflected in colloidal arrested phase separation and percolation-induced gel–gel phase separation in dilute polymer networks [3,5].

Network topology governs how local structural features translate into macroscopic aerogel properties. Above the connectivity threshold, a continuous network supports load transfer, mass transport, and thermal pathways, whereas isolated clusters below this threshold cannot sustain long-range mechanical or transport functions [12]. Although aerogels are often described as fractal-like or hierarchically connected networks, their topology is seldom idealized in practice: ligament thickness, pore-size distribution, interparticle necking, and connectivity vary across multiple length scales. Density-based scaling laws are therefore useful for comparative analysis but should not be treated as universal predictors of aerogel performance [12,13]. Mechanical response, thermal transport, dimensional stability, acoustic damping, and sorption behavior depend not only on density and porosity but also on network connectivity, spatial heterogeneity, tortuosity, and load-bearing pathways.

After initial network formation, wet gels continue to evolve through aging and coarsening. These processes are driven by the reduction in interfacial energy but are constrained by network elasticity, connectivity, and viscoelastic relaxation. Ostwald-ripening-type mechanisms, in which smaller domains dissolve and redeposit onto larger ones, provide a classical reference for curvature-driven coarsening [14]. In heterogeneous porous systems, such coarsening can be represented at the continuum scale, but gel networks often deviate from classical diffusion-limited behavior because the elastic framework restricts mass transport and redistributes stress [15,16]. In viscoelastic gels, phase separation, ripening, and mechanical relaxation are further coupled, making coarsening dependent on network mechanics and relaxation dynamics [17]. Aging can strengthen interparticle junctions and improve structural integrity, but excessive coarsening may reduce fine porosity, enlarge the characteristic pore size, weaken hierarchical organization, and alter transport or insulation performance.

The conversion of a wet gel into a dry aerogel introduces another critical design constraint: capillary-driven deformation. During solvent removal, curved liquid–vapor interfaces generate Laplace pressure, with smaller pores experiencing higher capillary stress [18]. If this stress remains below the effective mechanical resistance of the network, shrinkage can be limited and connectivity preserved. If it exceeds the gel’s elastocapillary resistance, however, irreversible shrinkage, pore collapse, cracking, or loss of network integrity may occur, particularly when the network cannot redistribute stress during solvent removal [16,18]. Supercritical drying minimizes capillary stress but requires specialized equipment and high energy input; freeze-drying can preserve anisotropic or fibrous architectures but introduces ice-templating effects; and ambient-pressure drying is more scalable but requires careful control of solvent exchange, surface chemistry, and network stiffness. Drying is therefore not merely a post-processing step; it is a structure-defining and sustainability-relevant stage in aerogel design. This drying-centered view provides a mechanistic bridge to the material-specific discussions that follow.

**Figure 1:**
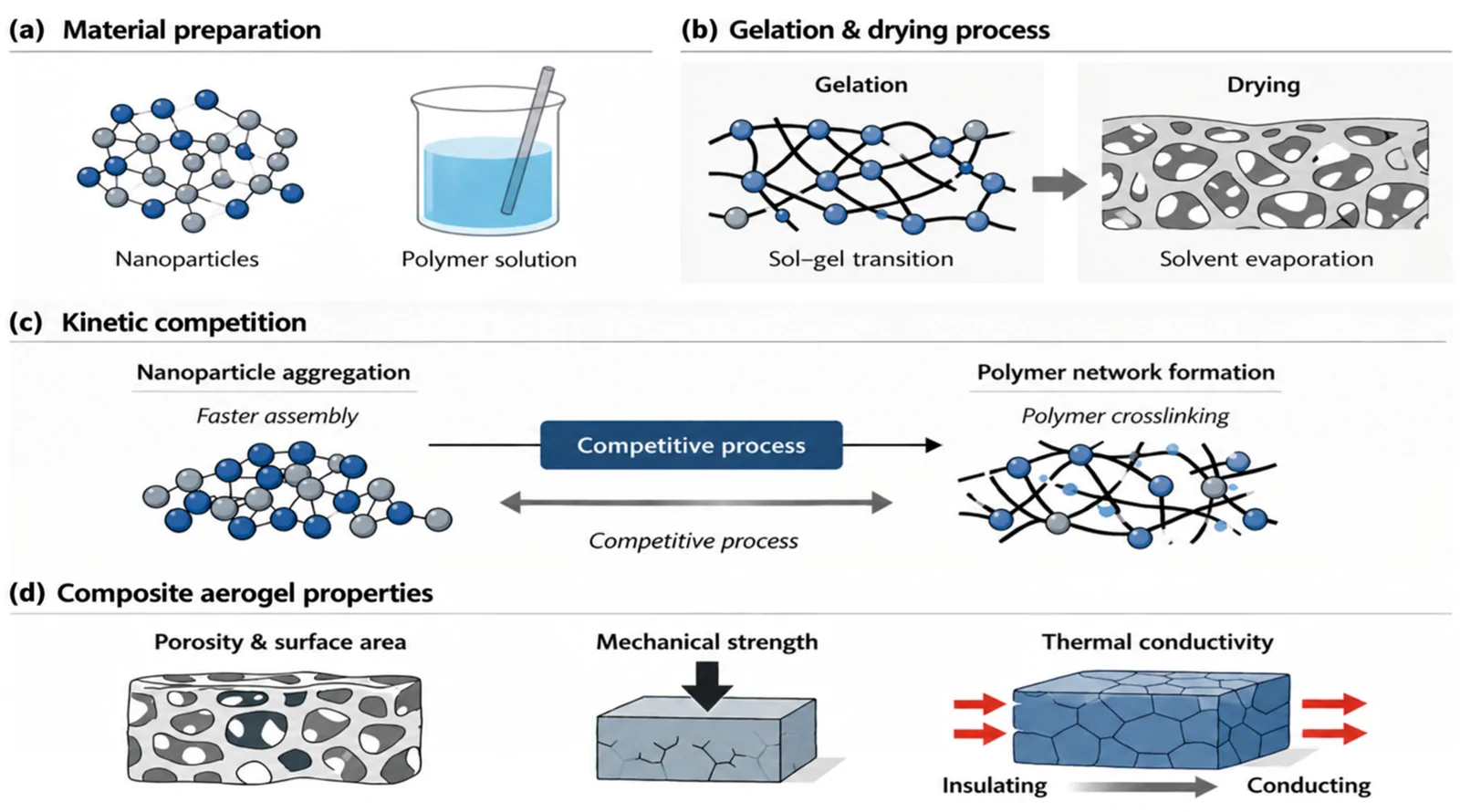
Universal mechanistic pathway of polymer aerogel formation.

Overall, polymer aerogel architecture emerges from the coupled sequence of nonequilibrium gelation, phase separation, kinetic arrest, topology development, aging, and drying. This framework explains why aerogel morphology should be treated as a pathway-dependent architecture rather than as a direct consequence of precursor chemistry alone. In biomass-derived aerogels, these processes are modulated by biopolymer functionality, supramolecular interactions, crystallinity, moisture sensitivity, and feedstock variability. In thermoset, hybrid, and recycled aerogels, they are governed by curing kinetics, crosslink density, phase compatibility, filler–binder interactions, dimensional stability, and value retention after processing. These distinctions establish the general structure-forming principles needed to compare renewable, thermoset, dynamic, hybrid, and recycled aerogel systems through a common cleaner-production lens. Figure 1 synthesizes this mechanistic framework by illustrating how precursor assembly, gelation–phase separation coupling, kinetic arrest, topology evolution, aging, and drying collectively encode polymer aerogel architecture and govern structural preservation across biomass-derived, thermoset, hybrid, and recycled systems.

Schematic representation of the coupled nonequilibrium processes that govern polymer aerogel formation and structural preservation. Molecular precursors, colloidal particles, fibers, or waste-derived building blocks first undergo sol–gel transition to form a percolating network. In parallel, reaction–diffusion competition regulates the relative rates of network formation and phase separation, leading to spinodal decomposition, nucleation–growth, or intermediate morphology-selection pathways. Kinetic arrest fixes transient structures before equilibrium is reached, while aging and coarsening further modify ligament thickness, pore size, and network connectivity. Solvent exchange and drying then determine the extent of capillary stress, structural preservation, shrinkage, or collapse. This unified framework links gelation, phase separation, topology development, aging, and drying, providing the mechanistic foundation for all polymer aerogel systems. Arrows indicate the direction of structural evolution and the coupling between sequential formation processes.

### 2.2. Multiscale Design Principles for Structural Control

Multiscale structural control is a central design requirement for polymer aerogels because macroscopic performance emerges from the coordinated organization of structural features across molecular, nanoscale, mesoscale, and macroscopic levels. This hierarchy is a core principle in architected and hierarchical materials, where local interactions and structural motifs propagate across length scales to determine mechanical and transport behavior [19]. At the molecular scale, precursor geometry, functional groups, chain rigidity, interaction anisotropy, and bonding chemistry define the initial assembly landscape. These molecular attributes guide the formation of nanoscale ligaments, particles, fibers, or domains, as demonstrated in hierarchical structures generated from nanocluster mesophases and compositionally anisotropic molecular building blocks [20,21]. These nanoscale features subsequently organize into mesoscale pore networks characterized by pore-size distribution, connectivity, tortuosity, anisotropy, and spatial heterogeneity. At the macroscopic scale, hierarchical organization governs mechanical response, thermal transport, acoustic damping, sorption behavior, and multifunctional performance; therefore, aerogel properties arise from interacting structural descriptors rather than from a single metric such as density or porosity [22].

This multiscale perspective explains why density and porosity, although useful first-order descriptors, are insufficient to predict aerogel performance. Density-based scaling remains valuable for comparing porous and architected materials, but its predictive capacity decreases when network topology, pore connectivity, ligament morphology, and anisotropy vary across systems [23,24]. Mechanical properties are governed by load-bearing pathways and deformation mechanisms, whereas thermal conductivity reflects the combined contributions of solid conduction, gas-phase transport, and radiative transfer. Thermal-transport studies further show that heat flow can be redirected, suppressed, or enhanced through phonon engineering, structural anisotropy, and thermal metamaterial design, underscoring the importance of architecture beyond density alone [25,26]. Similarly, sorption and separation performance depend on pore accessibility, tortuosity, surface chemistry, and transport resistance. Generalized structure–property scaling should therefore be treated as an interpretive framework rather than as a universal predictive law, particularly for heterogeneous aerogels whose processing histories, characterization methods, and testing conditions differ across studies [24].

Processing provides the practical route through which multiscale architecture is encoded. As established in Section 2.1, aerogel formation proceeds through nonequilibrium pathways involving aggregation, phase separation, gelation, kinetic arrest, aging, solvent exchange, and drying. In this context, processing parameters do not merely tune a pre-existing structure; they select trajectories through a kinetic landscape. Reaction rate, diffusion, solvent quality, quench depth, crosslinking density, freezing direction, drying route, and post-treatment conditions determine whether fine homogeneous networks, coarser phase-separated structures, anisotropic channels, or collapsed architectures are obtained [4]. This processing–structure coupling is also a major source of variability in reported aerogel properties, underscoring the need for complete reporting of synthesis history, drying conditions, shrinkage, density, testing geometry, and environmental conditions [24].

Architectural design strategies provide additional routes for directing hierarchical porosity and functional response. Templating can impose spatial constraints or exploit internally generated patterns to regulate pore size and connectivity, while directional structuring introduces anisotropy through freezing, gradients, alignment, or spatially biased assembly [27]. Geometry-driven design and additive manufacturing further expand the accessible design space by defining pore topology and connectivity more explicitly, as demonstrated in architected cellular materials and hydrogel-mediated micro-architected structures [28,29]. More broadly, recent developments in three-dimensional architected materials show how structural control across length and time scales can tune mechanical and transport properties [30]. However, these approaches do not operate independently of material chemistry or formation kinetics. Their effectiveness depends on whether the intended architecture is retained during gelation, aging, solvent exchange, drying, and post-processing.

Hybridization adds another level of structural and functional complexity by integrating components with different compositions, morphologies, or roles within a single porous network. In hybrid aerogels, mechanical reinforcement, thermal insulation, electrical conductivity, adsorption capacity, flame resistance, catalytic activity, or sensing capability can be combined through the spatial organization of polymers, biopolymers, inorganic particles, carbonaceous phases, fibers, or recycled constituents. Such multifunctionality is rarely additive: improving one property can alter others through changes in pore architecture, interfacial compatibility, network stiffness, or transport pathways. Mechanically strengthened and multifunctional aerogels therefore require multiscale, multicompositional, and multidimensional design strategies that balance component distribution, interfacial interactions, and network connectivity [31,32].

**Figure 2:**
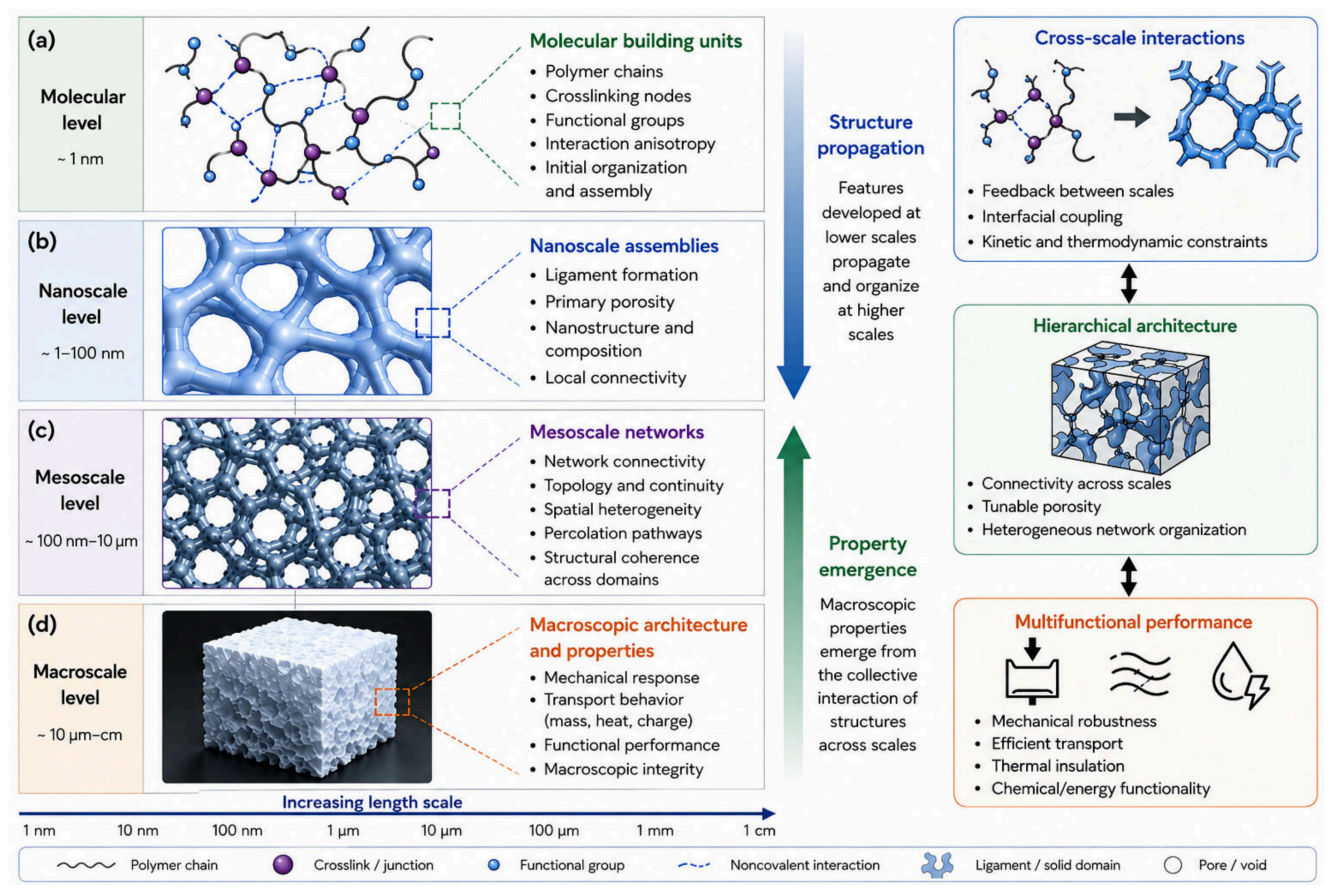
Multiscale structural descriptors governing polymer aerogel performance.

Overall, multiscale structural control bridges universal formation mechanisms with material-specific aerogel families. For biomass-derived aerogels, multiscale design must account for biopolymer functionality, crystallinity, fibrillar assembly, moisture sensitivity, and feedstock variability. For thermoset, dynamic, hybrid, and recycled aerogels, it must address curing kinetics, crosslink density, phase compatibility, filler distribution, reprocessability, and structural retention during drying or reuse. In both cases, the central design challenge is not simply to maximize porosity or minimize density, but to engineer hierarchical architectures that deliver functional performance per unit material input, processing energy, and environmental burden. Figure 2 summarizes this multiscale design framework by showing how molecular interactions, nanoscale structural motifs, mesoscale pore-network descriptors, and macroscopic architecture collectively govern polymer aerogel performance.

Schematic illustration of how molecular precursors, nanoscale ligaments or domains, mesoscale pore networks, and macroscopic architectures collectively determine polymer aerogel performance. Molecular interactions define assembly pathways; nanoscale structures form pores and load-bearing elements; mesoscale connectivity regulates transport, tortuosity, and spatial heterogeneity; and macroscopic organization governs mechanical, thermal, acoustic, and sorption behavior. Arrows represent the hierarchical progression of structural information across different length scales and the relationship between structural features and resulting properties.

### 2.3. Fundamental Design Constraints and Multidimensional Trade-Offs

The mechanistic framework established in Section 2.1 and the multiscale design principles discussed in Section 2.2 indicate that polymer aerogel design should not be framed as an unconstrained pursuit of isolated property maximization. Rather, aerogel performance emerges within a bounded, multidimensional design space in which structure formation, hierarchical organization, processing history, durability, and sustainability are intrinsically coupled. Density, porosity, mechanical integrity, thermal insulation, adsorption capacity, dimensional stability, scalability, and circularity cannot be optimized independently. Improving one attribute often imposes penalties on another. Sustainable polymer aerogels should therefore be assessed not only by peak performance metrics but also by their ability to balance functional output with material input, processing intensity, service lifetime, and end-of-life value retention.

A key constraint arises from the coupling among density, mechanical response, and thermal transport. Lowering density can reduce solid-phase heat conduction and improve thermal insulation, but heat transport remains coupled to solid conduction, gas-phase transport, radiative transfer, and structural pathways [25]. At the same time, reduced density decreases the number of load-bearing pathways and can increase susceptibility to shrinkage, buckling, cracking, or pore collapse. Conversely, increasing the solid fraction, crosslink density, or reinforcement content can enhance stiffness and dimensional stability, but may also increase thermal conductivity, restrict pore accessibility, or reduce mass-normalized or function-specific performance. Reinforcement, hybridization, and multiscale strengthening can shift this balance by improving mechanical robustness; however, these strategies also modify pore connectivity, ligament morphology, and transport pathways [31,32].

A second design constraint concerns the balance between structural stability and network adaptability. Stable aerogel networks are necessary for preserving pore connectivity during aging, solvent exchange, drying, handling, regeneration, and use. However, highly constrained networks may have limited capacity for stress relaxation, damage repair, deformation recovery, or reprocessing. Viscoelastic relaxation and phase separation can redistribute stress and alter coarsening pathways, illustrating why stability and adaptability are coupled rather than independent variables [17]. Responsive or dynamically architected materials provide routes toward recoverability and adaptability, although such adaptability may introduce trade-offs in dimensional persistence or long-term stability [27]. Arrested phase separation in hydrogels further illustrates how kinetic structure fixation can enhance strength while reducing hysteresis, but these benefits remain pathway-dependent [4,6]. This stability–adaptability continuum provides an important bridge to the material-specific discussions in Section 3 and Section 4: biomass-derived aerogels are shaped by renewable feedstocks, abundant functional groups, hydrogen bonding, moisture response, and feedstock variability, whereas thermoset, hybrid, and dynamic covalent aerogels are governed by crosslinking chemistry, network permanence, reprocessability, structural retention, solvent demand, and processing burden.

Structural complexity introduces a further trade-off. Hierarchical porosity, anisotropic channels, multicomponent reinforcement, and multifunctional architectures can enhance mechanical resilience, thermal insulation, acoustic damping, oil uptake, adsorption, electrical conductivity, or catalytic performance. In energy-related hybrid systems, integrating ion-conducting or solid-electrolyte-like components may enhance ionic transport and chemical stability, but such functionality must be balanced against pore preservation, interfacial compatibility, and mechanical robustness [33]. Greater structural and compositional complexity also increases the number of variables that must be controlled during synthesis and processing, including precursor quality, solvent composition, reaction kinetics, phase separation, freezing conditions, binder distribution, solvent exchange, drying stress, and post-treatment [30,34]. Consequently, highly engineered aerogels may exhibit excellent laboratory-scale performance but face challenges in reproducibility, scale-up, and process robustness. Complete reporting of synthesis history, processing conditions, and testing methods is therefore essential for comparing heterogeneous aerogel systems and translating laboratory performance into reproducible material platforms [24,35].

Taken together, these considerations indicate that sustainable polymer aerogels occupy a bounded design space defined by coupled constraints rather than by a single limiting factor. Ultralow density, high stiffness, low thermal conductivity, high adsorption capacity, long-term stability, scalability, and recyclability are all desirable, but they are rarely maximized simultaneously. A cleaner-production perspective therefore requires service-normalized optimization. The central question is not whether an aerogel exhibits the lowest density, highest surface area, or best single-property value, but whether it delivers the required function with acceptable material consumption, processing intensity, durability, and end-of-life value. This framing enables biomass-derived, thermoset, dynamic, hybrid, and recycled aerogels to be compared through a common design logic rather than through isolated performance records.

Overall, Section 2 establishes that polymer aerogel design is governed by nonequilibrium formation, multiscale structural control, and multidimensional trade-offs. The following sections apply this framework to specific families of materials. Section 3 examines biomass-derived aerogels, where renewable origin, biopolymer functionality, moisture response, and feedstock variability define the main opportunities and constraints. Section 4 considers thermoset, dynamic, hybrid, and recycled polymer aerogels, where crosslinking chemistry, network permanence, reprocessability, and structural retention become central. Across both sections, the key challenge is to design aerogels that occupy favorable regions of the bounded design space, delivering service-relevant performance while reducing environmental burden across the full material life cycle.

Literature search strategy. The evidence base for this review was assembled from Web of Science, Scopus and Google Scholar using combinations of the terms “aerogel”, “polymer aerogel”, “biopolymer/cellulose/lignin/chitosan aerogel”, “thermoset aerogel”, “vitrimer”, “dynamic covalent network”, “life-cycle assessment”, “circular economy”, “waste valorization”, “machine learning” and “inverse design”, with emphasis on publications from 2018 onward and with foundational earlier work retained where it defines a mechanism or method. Records were screened first by title and abstract and then by full text. Studies were included when they reported a defined aerogel or aerogel-derived architecture; at least one quantitatively characterized structural or functional property; and sufficient synthesis or processing detail to permit interpretation. Studies were excluded when the material was better described as a foam, sponge, or xerogel without an aerogel-type network, when properties were reported without processing context, or when sustainability claims were made without supporting data. Because the field is broad and heterogeneous, this review is intended as a critical and integrative synthesis rather than a PRISMA-type systematic review; no meta-analysis was performed, and the property maps in Section 5 are conceptual rather than statistically derived.

## 3. Fundamental Design Framework of Biomass-Derived Aerogels

**Figure 3:**
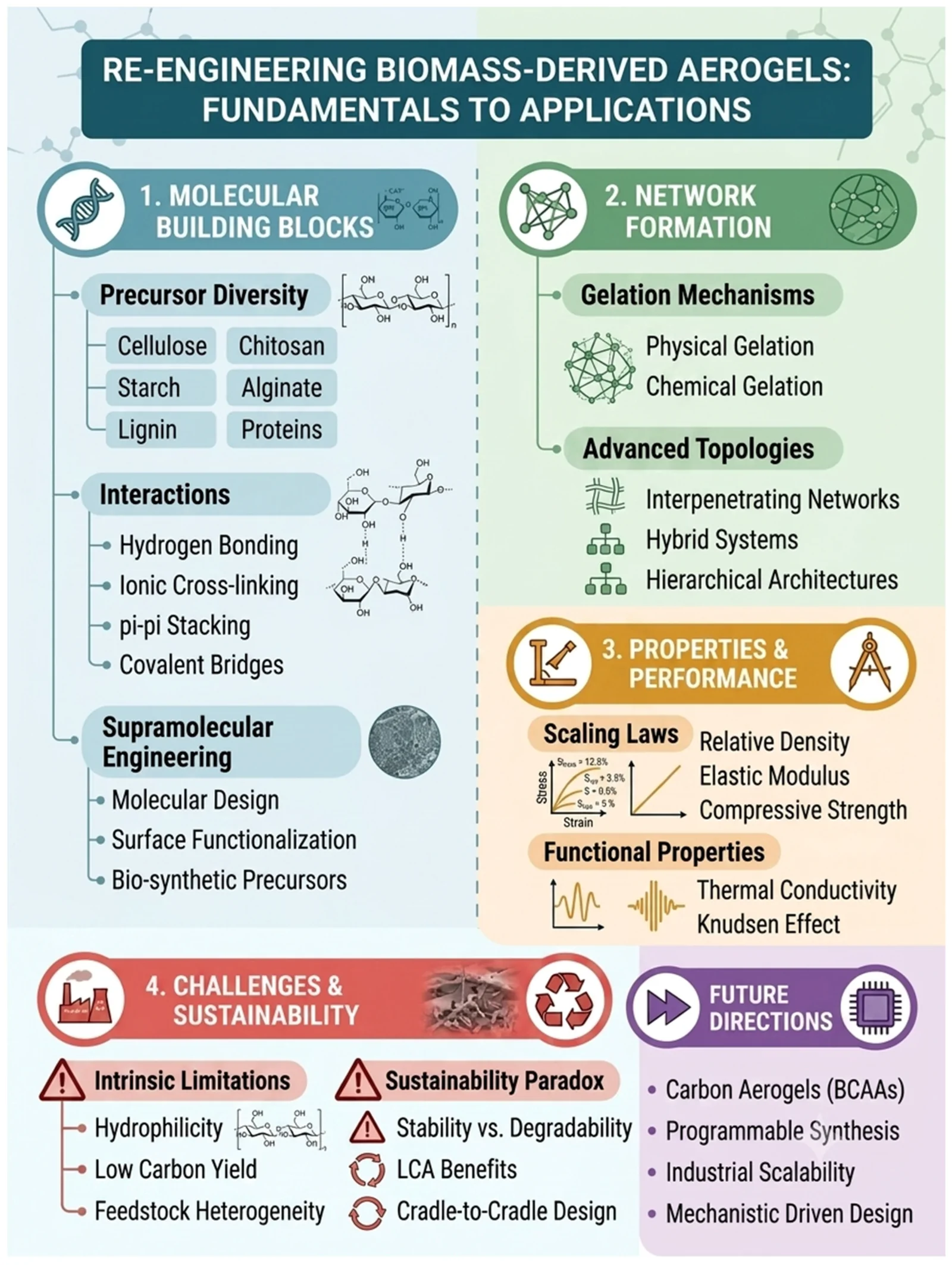
Comprehensive framework for re-engineering biomass-derived aerogels. Schematic illustration of the integrated pathway from molecular building blocks and network formation to properties engineering and future industrial perspectives.

This section is structured according to the multiscale design framework illustrated in Figure 3, spanning molecular building blocks, network formation, properties and performance, and sustainability-related challenges.

### 3.1. Precursor Chemistry and Molecular Interactions

Molecular building blocks represent the fundamental design layer of biomass-derived aerogels, governing the intrinsic structure–property–function relationships [24,36]. In contrast to synthetic polymers with well-defined and uniform architectures, biomass-derived precursors exhibit inherent heterogeneity, hierarchical organization, and multifunctional chemical functionalities [37,38]. While these characteristics provide considerable structural versatility, they also introduce challenges in achieving precise molecular-level control and ensuring batch-to-batch reproducibility [4].

#### 3.1.1. Precursor Diversity

Biomass-derived aerogels are constructed from a diverse spectrum of renewable molecular building blocks, whose chemical structures and network-forming mechanisms determine the resulting architecture and functionality [24,36,39]. Among these, cellulose is the most extensively studied precursor [40,41,42]. Its linear chains of D-glucose units, linked via β-1,4-glycosidic bonds, enable the formation of robust hydrogen-bonded networks [40]. While plant-derived cellulose typically requires purification to remove hemicellulose and lignin, bacterial cellulose (BC) is inherently ultrapure and self-assembles into highly entangled nanofibrillar networks [43], allowing the fabrication of aerogels with ultrahigh porosity (up to 99%) and ultralow densities (0.005–0.2 g cm^−3^) [44,45].

Building on hydrogen-bond-dominated systems, chitin and its deacetylated derivative, chitosan, introduce additional chemical functionality [46]. The presence of amino (–NH_2_) and hydroxyl groups enables versatile intermolecular interactions and provides active sites for nitrogen self-doping during carbonization, pH-responsive behavior, and metal-ion coordination [46]. In contrast, starch follows a different structural pathway governed by the interplay between linear amylose and branched amylopectin [47]. Its gelation behavior is dominated by a gelatinization–retrogradation process, in which thermal disruption and subsequent reorganization of hydrogen bonds result in semicrystalline and mechanically compliant networks [48].

A more controllable gelation mechanism is observed in sodium alginate, a linear anionic polysaccharide that forms networks through ionic crosslinking [49,50]. Specifically, guluronic (G) acid residues coordinate with multivalent cations (e.g., Ca^2+^, Cu^2+^) to form the characteristic “egg box” structure, enabling rapid and tunable network formation under mild conditions [38]. In contrast to these well-defined gel-forming systems, lignin exhibits an irregular aromatic macromolecular structure and limited solubility, which generally restrict its role to that of a reinforcing phase or a high-carbon-yield precursor, thereby improving thermal stability and mechanical robustness in composite systems [51,52,53].

Protein-based precursors, such as gelatin and silk fibroin [54], introduce a different assembly mechanism associated with hierarchical secondary structures (e.g., α-helices and β-sheets) [55,56]. These structures enable gelation via temperature-induced self-assembly and provide advantages in biocompatibility as well as tunable mechanical behavior [57,58].

The choice of these molecular building blocks influences both the sustainability and multiscale organization of biomass-derived aerogels. However, the intrinsic heterogeneity of natural biomass remains a key limitation for reproducibility [4,36]. To address this issue, recent efforts have focused on biosynthetic precursors, such as artificial starch synthesized directly from CO_2_, which enable improved control over molecular weight and purity. This approach provides a promising pathway toward scalable production with more consistent and predictable performance [36].

#### 3.1.2. Molecular and Supramolecular Interactions

The abundance, chemical nature, and spatial distribution of functional groups, primarily hydroxyl (–OH), amino (–NH_2_), carboxyl (–COOH), and aromatic moieties, govern the multiscale structural evolution of biomass-derived aerogels through hydrogen bonding, ionic crosslinking, π–π stacking, and covalent bridging interactions [24,59]. These interaction modes define the pathways of network formation, from physically assembled structures to chemically stabilized frameworks.

In polysaccharide-based systems such as cellulose and starch, hydrogen bonding is the dominant interaction [47,60]. In cellulose, extensive intermolecular hydrogen bonds drive fibrillar aggregation and network percolation, stabilizing nanofibrillar assemblies [60]. In starch, network formation proceeds through a gelatinization–retrogradation process, where thermal disruption and subsequent reassociation of hydrogen bonds lead to semicrystalline structures that are sensitive to water content and thermal history [48]. Chitosan extends this interaction framework through the presence of amino groups, which enable additional electrostatic interactions, particularly with negatively charged species such as graphene oxide, while also serving as active sites for nitrogen incorporation during carbonization [61].

Alginate-based systems, in contrast, are governed by ionic crosslinking [38]. The coordination between carboxylate groups in guluronic acid (G) blocks and multivalent cations (e.g., Ca^2+^, Zn^2+^) leads to the formation of “egg box” junctions, enabling rapid gelation and tunable network connectivity under mild conditions [49,62]. This mechanism provides more direct control over network formation compared to hydrogen-bonded systems.

Lignin-rich systems are primarily dominated by π–π stacking and van der Waals interactions arising from their aromatic, branched structures [63]. While hydroxyl groups still contribute to hydrogen bonding, interactions between aromatic rings enhance network rigidity and thermal stability [63,64]. Due to limited solubility, lignin is typically incorporated into hybrid systems as a reinforcing or carbon-rich phase rather than forming a continuous network [65].

In addition to these physical interactions, covalent bridges can be introduced through chemical crosslinking agents such as epichlorohydrin and glutaraldehyde, forming ether or imine linkages [66]. These covalent bonds are critical for improving mechanical strength and preventing capillary-induced collapse during ambient pressure drying (APD) [67]. In practice, material performance depends on the balance between reversible physical interactions, which allow structural rearrangement, and irreversible covalent crosslinks, which provide mechanical stability [66].

At a fundamental level, these interaction modes determine the kinetics of bond formation and structural arrest during gelation [68]. The selection and tuning of functional groups therefore act as a key design parameter, controlling non-equilibrium assembly pathways and ultimately dictating whether the resulting structure evolves into particulate or bicontinuous networks, as well as how hierarchical porosity is preserved [24,69].

#### 3.1.3. Supramolecular Engineering

Recent advances in supramolecular chemistry have driven a shift in biomass-derived aerogels from empirically optimized systems toward interaction-guided molecular design [59,66]. By tailoring noncovalent interactions such as hydrogen bonding, electrostatic interactions, and ionic coordination together with covalent crosslinking pathways, it is possible to regulate gelation kinetics, network connectivity, and the resulting framework topology [59].

Building on the alginate “egg box” mechanism described in Section 3.1.2, supramolecular engineering extends to other polysaccharides. In starch-based systems, supramolecular engineering relies on the gelatinization–retrogradation process to direct polymer rearrangement into semicrystalline, physically crosslinked networks, with the final structure strongly dependent on thermal history and water-mediated interactions [70].

Surface functionalization further expands the design space. For example, TEMPO-mediated oxidation introduces carboxyl groups onto cellulose nanofibers, enhancing electrostatic repulsion, improving colloidal stability, and enabling controlled self-assembly and crosslinking [59]. Similarly, chitosan provides reactive amino groups (–NH_2_) that can be tailored to promote intermolecular interactions and chemical modification, while also serving as intrinsic nitrogen sources during carbonization, leading to in situ heteroatom doping and improved electrochemical performance [61].

In parallel, the development of biosynthetic precursors introduces an additional level of control in supramolecular engineering. Artificial polysaccharides, such as starch synthesized from CO_2_, enable precise tuning of molecular weight, composition, and functional group distribution, thereby reducing the variability associated with natural biomass [36]. This improved control over precursor structure allows more predictable regulation of intermolecular interactions and network formation, which is critical for achieving reproducible aerogel architectures.

Collectively, these strategies establish a framework for linking molecular design to supramolecular assembly, enabling the rational transformation of biopolymer networks into biomass-derived carbon aerogels (BCAs) and other functional systems [24,36]. From a fundamental perspective, such approaches regulate interaction potentials and kinetic pathways governing bond formation and restructuring, thereby enabling control over non-equilibrium gelation and the emergence of distinct network morphologies [69].

### 3.2. Network Formation

#### 3.2.1. Gelation Mechanisms

Biomass-derived aerogels form through a kinetically governed sol–gel transition that can proceed via physical or chemical gelation mechanisms, where network topology is determined by nonequilibrium assembly pathways rather than thermodynamic equilibrium [71,72]. In this context, the competition between phase separation and network formation plays a central role in defining the final porous architecture.

Physical gelation in most biopolymer systems originates from a kinetically arrested phase separation process, in which polymer-rich domains undergo percolation and become “frozen” before macroscopic phase separation occurs [69]. This mechanism underlies the formation of the highly porous, bicontinuous structures characteristic of aerogels [34]. Such networks are typically stabilized by noncovalent interactions, including hydrogen bonding in cellulose, ionic coordination in alginate via the “egg box” model, and gelatinization–retrogradation in starch [48,49]. As a result, physically gelled systems are generally reversible and sensitive to environmental conditions such as temperature, pH, and solvent composition.

In contrast, chemical gelation involves the formation of permanent covalent cross-links, such as imine or ether bonds, leading to mechanically robust and structurally stable networks [66,73]. These covalent frameworks are more resistant to shrinkage and structural collapse during subsequent processing steps, including drying and carbonization, but exhibit limited reversibility compared to physically crosslinked systems. In practice, many aerogel systems combine both mechanisms, where physical interactions guide initial network formation, followed by covalent crosslinking to stabilize the structure.

The resulting pore structure is governed by the interplay between gelation kinetics and phase separation dynamics [68,74]. Rapid gelation suppresses domain coarsening, leading to finely interconnected nanoporous networks with high specific surface areas [68]. Conversely, slower gelation allows structural rearrangement and phase segregation, resulting in larger pores or particulate morphologies [68]. The aerogel structure can therefore be viewed as a kinetically arrested state, in which transient configurations are preserved during the sol–gel transition [72].

These observations highlight the importance of controlling processing parameters such as temperature, pH, and precursor concentration to regulate gelation kinetics and direct structural evolution [75]. From a design perspective, tuning the balance between physical and chemical gelation provides a practical route to tailor network connectivity, pore architecture, and ultimately the macroscopic performance of biomass-derived aerogels.

#### 3.2.2. Advanced Topologies

To address the inherent fragility of single-network aerogels, particularly at ultralow densities, advanced network architectures have been developed to enhance mechanical robustness and functional performance [76,77]. These strategies can be broadly categorized into interpenetrating networks, hybrid systems, and hierarchical architectures, each targeting different aspects of structural and functional optimization. Interpenetrating polymer networks (IPNs) represent a key approach, in which two or more independent networks are interwoven without covalent bonding between them, thereby improving stress distribution and mechanical toughness [76]. For example, bacterial cellulose hydrogels can act as preexisting scaffolds for the in situ polymerization of secondary networks, such as phenolic resins, resulting in synergistic reinforcement effects [40].

Hybrid network systems, in contrast, focus on compositional integration by combining organic polymer matrices with inorganic or conductive components. Representative examples include lignin integrated with nickel foam or graphene oxide, which introduce multifunctional properties such as enhanced electrical conductivity and electromagnetic interference (EMI) shielding [78]. These systems illustrate how compositional heterogeneity can be leveraged to extend functionality beyond that of single-component networks [79]. Hierarchical and architected aerogels further incorporate structural design across multiple length scales to optimize performance. By introducing bioinspired architectures such as gradient or lamellar structures via freeze casting or combining multiple phases (e.g., cellulose-chitosan and lignin-reinforced systems), these materials enable partial decoupling of mechanical, thermal, and transport properties [80]. In such systems, performance is governed not only by chemical composition but also by mesoscale geometry and network connectivity [34].

Despite these advantages, increasing structural complexity introduces challenges in interfacial compatibility, process control, and scalability, reinforcing the trade-off between performance optimization and manufacturability [78,79].

### 3.3. Properties and Performance

The molecular characteristics of biomass-derived building blocks exert a profound influence on mesoscale organization and macroscopic performance, reflecting a tightly coupled structure–property relationship across multiple length scales [24,79]. Even hybrid biomass aerogels combining multiple waste feedstocks, such as coffee-cellulose aerogels derived from spent coffee grounds reinforced with cotton fibers, illustrate how molecular building block selection directly controls macroscopic functional behavior, including porosity, hydrophobicity, and oil-absorption capacity [81]. Parameters such as chain rigidity, degree of polymerization, and interaction strength govern gelation kinetics, percolation behavior, and network connectivity, which collectively define the hierarchical pore architecture spanning nano, meso, and macroscales [34]. These structural features, in turn, dictate key functional properties, including mechanical strength, thermal conductivity, and mass transport [34,39]. However, such relationships are inherently constrained by trade-offs; for example, the incorporation of flame-retardant additives or functional modifiers may disrupt polymer chain continuity and weaken intermolecular interactions, thereby compromising mechanical integrity despite enhancing fire resistance [82,83]. Furthermore, the intrinsic heterogeneity of natural biomass manifested in variations in lignin content, crystallinity, and impurity levels introduces significant variability in gelation behavior and final material properties, often leading to broad dispersion in reported performance metrics [4,84]. This variability underscores the need for standardized characterization protocols and highlights the importance of developing biosynthetic or structurally controlled precursors, such as artificial starch synthesized from CO_2_ [85].

To enable predictive design, these qualitative structure–property relationships can be formalized through quantitative scaling laws, which link network topology and relative density to macroscopic performance [4,79]. In analogy to cellular solids, the elastic modulus (E) and compressive strength (σ) scale with relative density (ρ) according to power-law relationships, E ∝ ρ^n^ and σ ∝ ρ^m^, where n and m typically range from 2 to 4 depending on deformation mechanisms and network connectivity [79]. Deviations from classical scaling are observed in highly crystalline systems such as bacterial cellulose, where enhanced load transfer enables superior mechanical performance at high porosity [40].

Thermal transport is governed by pore size and connectivity, with thermal conductivity of biopolymer-derived aerogels typically reported in the range of 30–40 mW m^−1^ K^−1^ [34,39]. As reviewed for nanoporous media generally [86], the Knudsen effect suppresses gas-phase conduction at the nanoscale, while solid conduction and radiative contributions become increasingly important depending on density and temperature. In carbonized systems, radiative heat transfer dominates at elevated temperatures (>500 °C), necessitating the incorporation of opacifiers [39].

These scaling relationships define a constrained performance envelope, within which key properties such as density, mechanical strength, and thermal conductivity are intrinsically coupled [24], highlighting the challenge of simultaneously optimizing multiple functional metrics in biomass-derived aerogels.

An important subclass comprises nanocellulose-based hybrid aerogels [45] modified with siloxane polymers, because they help address the moisture sensitivity that otherwise limits bio-based aerogels. Hydrolysis and condensation of alkoxysilanes such as methyltrimethoxysilane or polyvinyltrimethoxysilane generate a siloxane network that is co-assembled with the cellulose skeleton, conferring durable hydrophobicity, improved dimensional stability under humid conditions, and, in several systems, synergistic char formation and flame retardancy. Polyvinyltrimethoxysilane-enhanced TEMPO-oxidized cellulose nanofiber aerogels, for example, combine anisotropic thermal insulation with flame retardancy and oil/water separation capability [87]. Fully siloxane-based analogs show the performance achievable with this chemistry: polyvinylpolymethylsiloxane/polydimethylsiloxane copolymer aerogels reach a water contact angle of 151°, a density of 109 mg cm^−3^, and a thermal conductivity of 29.8 mW m^−1^ K^−1^ while remaining compressible and machinable [88], and triple-network graphene/polyorganosiloxane systems extend the same architecture toward multifunctional sensing [89].

Siloxane modification, however, also introduces a clear cleaner-production trade-off. The introduction of a synthetic, fossil-derived, and non-biodegradable component means that the renewable-carbon fraction must also be considered: moisture resistance and service lifetime are extended, which improves the service-based functional unit, but the end-of-life pathway shifts from compostability toward a hybrid organic–inorganic residue that is neither readily biodegradable nor covered by established polymer-recycling routes. Silane processing also typically requires alcoholic solvents and additional exchange steps, increasing solvent intensity. Its overall benefit therefore depends on the application and service lifetime, and should be assessed using the service-based functional units and reporting descriptors set out in Section 7 and Section 8 rather than assumed from improved laboratory performance alone.

### 3.4. Challenges and Sustainability

Biomass-derived aerogels are intrinsically constrained by physicochemical boundaries that define a limited and interdependent design space [24,36]. At ultralow densities, sparse network connectivity leads to insufficient load-bearing capacity, while the abundance of hydrophilic functional groups promotes moisture uptake, resulting in swelling, plasticization, and long-term structural degradation [37].

Thermal processing further imposes critical limitations, as biomass precursors undergo extensive fragmentation and volatilization during pyrolysis, leading to low carbonization yields, significant mass loss, and network shrinkage [85]. In addition, the intrinsic heterogeneity of biomass feedstocks introduces batch-to-batch variability in gelation behavior and final material properties, limiting reproducibility [24,90].

From a sustainability perspective, strategies such as surface hydrophobization improve environmental stability but introduce a sustainability paradox, wherein extended degradation timescales compromise material circularity [91]. At the same time, life-cycle assessment (LCA) studies have demonstrated that biomass-derived aerogels can offer substantial reductions in carbon emissions compared to conventional inorganic counterparts, particularly when derived from agricultural residues [39,92].

This duality highlights a fundamental trade-off between performance and environmental impact. In this context, the concept of cradle-to-cradle design becomes increasingly relevant, emphasizing not only renewable sourcing but also end-of-life recyclability and material circularity [92]. Achieving this balance requires integrating molecular design, processing strategies, and life-cycle considerations to minimize environmental burden while maintaining functional performance.

Collectively, these constraints highlight that performance trade-offs arise from fundamental material characteristics rather than solely from processing conditions [37].

### 3.5. Future Directions and Design Gaps

The limitations outlined above define a bounded performance envelope that cannot be overcome by incremental optimization alone and therefore require more advanced design strategies [4,36]. Key design gaps include the absence of standardized precursor systems, intrinsic feedstock heterogeneity, and low carbonization yields, all of which hinder reproducibility and limit industrial scalability [85].

To address these challenges, various structural and material innovations have been proposed. Hybrid and interpenetrating polymer networks (IPNs) have been developed to enhance load transfer and mechanical robustness; however, these approaches often introduce increased processing complexity and energy consumption, particularly during drying [67]. More fundamentally, the transition toward biomass-derived carbon aerogels (BCAs) represents a transformative pathway in which organic precursor architectures are converted into conductive, high-surface-area carbon frameworks. This evolution enables advanced functionalities such as electrochemical energy storage and electromagnetic interference shielding that are not accessible in purely organic systems [39,93].

In parallel, the development of biosynthetic precursors, such as artificial starch derived from CO_2_, offers a promising route toward molecular-level precision, improved reproducibility, and scalable manufacturing [85]. These advances open opportunities for programmable synthesis, where precursor chemistry, intermolecular interactions, and processing parameters can be systematically tuned to direct gelation pathways and control network formation across multiple length scales.

Collectively, these developments signal a shift from empirical optimization toward mechanism-driven and multiscale design, in which the underlying physics of gelation, phase separation, and structural arrest are explicitly leveraged to engineer aerogel architectures. Such an approach enables predictive control over structure–property relationships, rather than relying on trial-and-error optimization.

From a sustainability perspective, biomass-derived building blocks provide inherent advantages, including renewability, low toxicity, and potential carbon neutrality, making them attractive alternatives to conventional inorganic aerogel precursors [24,92]. Life-cycle assessments of bio-based insulation materials indicate that renewable and residue-derived feedstocks can lower cradle-to-gate climate impact relative to conventional petrochemical and mineral alternatives, although the magnitude of the benefit is strongly dependent on the declared system boundary, functional unit, and drying route, and direct comparisons with silica aerogels remain scarce [59,73,94]. Nevertheless, challenges related to long-term stability, structural durability, and energy-intensive processing such as solvent exchange and drying remain significant barriers [95].

Importantly, molecular design plays a critical role in determining sustainability outcomes. Strategies such as solvent-free or low-solvent surface modification can reduce secondary environmental impacts, while the use of inherently reactive or self-assembling precursors may eliminate the need for external crosslinking agents [66,92]. These considerations highlight the importance of an integrated design framework that simultaneously accounts for molecular structure, supramolecular interactions, processing conditions, and life-cycle performance. In this context, the concept of “cradle-to-cradle” design becomes increasingly relevant, emphasizing both resource origin and end-of-life recyclability.

Overall, future progress in biomass-derived aerogels will depend on the convergence of molecular engineering, advanced network design, and sustainability-driven strategies to achieve scalable, high-performance, and environmentally responsible material systems [85].

## 4. Thermoset-Based and Recycled Thermoset Aerogels

Following the discussion of biomass-derived aerogels in Section 3, thermoset-based aerogels provide a complementary case in which structural stability is governed by chemically defined covalent networks rather than naturally heterogeneous biopolymer assemblies. Biomass-derived systems offer renewable carbon sources, abundant functional groups, and diverse assembly pathways, but their performance may be constrained by precursor variability, moisture sensitivity, limited thermal stability, and batch-to-batch differences in gelation behavior [24,36,39]. In contrast, thermoset aerogels, including phenolic, resorcinol–formaldehyde, polyimide, polyurethane, polyurea, and epoxy-based networks, are valued for their thermal resistance, chemical stability, dimensional integrity, and mechanical robustness, making them attractive for structural insulation, aerospace thermal protection, flame-resistant components, and high-temperature thermal management [61,71,72].

However, the same permanent covalent crosslinks that provide thermoset aerogels with high durability also create their central sustainability limitation. Conventional thermosets cannot be remelted, reshaped, welded, or efficiently reprocessed, which restricts repair, recycling, and closed-loop material recovery [51,79,80]. This limitation is particularly important for aerogels because their environmental benefit depends not only on use-phase performance, such as thermal insulation or long service lifetime, but also on production-stage burdens associated with curing, solvent exchange, drying, shrinkage control, and possible end-of-life losses [53,59,94]. Thermoset aerogels therefore represent a critical performance–circularity paradox: high crosslink density improves structural stability and service durability, but it also promotes linear material flows and limits end-of-life value retention.

Recent advances in dynamic covalent networks, vitrimer-like systems, chemically recyclable thermosets, and waste-derived polymer aerogels provide emerging routes to address this conflict. Associative or reversible bond-exchange chemistries, including transesterification, imine exchange, boronic ester exchange, disulfide exchange, vinylogous urethane exchange, and siloxane exchange, can introduce stress relaxation, self-healing, reprocessability, and network regeneration while retaining thermoset-like connectivity during service [69,76,96,97]. In parallel, recycled PET aerogels, polypropylene-fiber aerogels, hybrid PET–silica aerogels, fly-ash/PET composite aerogels, carbon nanotube aerogel composites, and multifunctional aerogel-inspired materials demonstrate that post-consumer or secondary resources can be converted into lightweight porous materials for insulation, oil/water separation, oil-spill remediation, acoustic absorption, radiative cooling, electromagnetic wave absorption, and construction applications [98,99,100]. These strategies are promising, but they also introduce new trade-offs involving bond-exchange kinetics, creep resistance, reprocessing energy, solvent demand, property retention, feedstock variability, and scalability. This section therefore examines thermoset-based and recycled thermoset aerogels through a concise framework linking network chemistry, porous architecture, performance–recyclability trade-offs, recycling pathways, and circular design limits.

### 4.1. Thermoset Aerogels as High-Performance but Non-Circular Networks

Thermoset aerogels occupy a performance-rich region of the polymer aerogel design space because their porous structures are stabilized by covalently crosslinked networks. Compared with biomass-derived aerogels, whose structures often rely on hydrogen bonding, ionic interactions, crystallinity, and other reversible or semi-reversible associations, thermoset aerogels provide stronger molecular-level fixation and improved resistance to collapse, shrinkage, thermal degradation, chemical attack, and mechanical deformation. In the multiscale framework introduced in Section 2, this means that thermoset chemistry controls not only molecular connectivity but also network topology, gelation kinetics, pore preservation during drying, and macroscopic thermal or mechanical performance. This makes thermoset aerogels important benchmarks for evaluating whether more sustainable aerogels can retain high service performance while reducing environmental burden.

Conventional thermoset aerogels, including phenolic, resorcinol–formaldehyde, polyimide, polyurethane, polyurea, and epoxy-based systems, have been widely investigated because they combine low density and hierarchical porosity with mechanical robustness, thermal resistance, and chemical stability [61,71,72,101]. Phenolic and resorcinol–formaldehyde aerogels are typically formed through polycondensation reactions that generate rigid aromatic crosslinked frameworks with good thermal stability and char-forming ability. Polyimide aerogels are commonly prepared from dianhydride and diamine monomers through polyamic acid formation followed by imidization, producing thermally stable networks whose toughness can be improved through backbone flexibility, crosslinker design, and nanofibrous or lamellar reinforcement [66,102,103,104,105]. Epoxy, polyurethane, and polyurea aerogels further illustrate how curing chemistry, segment rigidity, and hard–soft phase organization can be tuned to control stiffness, toughness, and dimensional stability.

Despite these advantages, conventional thermoset aerogels remain intrinsically difficult to place within a circular material economy. Once gelation and curing are completed, the network topology is fixed by irreversible covalent bonds. This prevents remelting, reshaping, and efficient reprocessing, so end-of-life options are often limited to disposal, incineration, mechanical downcycling, thermochemical conversion, or chemically intensive recovery routes [51,79,80]. The same design feature is therefore both an advantage and a limitation for thermoset aerogels: permanent crosslinking improves structural fixation, but it eliminates intrinsic pathways for repair, reconfiguration, and closed-loop recovery. For this reason, conventional thermoset aerogels should be viewed as high-performance but non-circular reference systems rather than inherently sustainable materials.

This limitation becomes more significant when thermoset aerogels are evaluated through a cleaner-production lens. Their low thermal conductivity and long service lifetime may reduce use-phase energy demand in insulation or thermal protection applications, but these benefits must be balanced against solvent use, drying energy, curing conditions, processing losses, and end-of-life value loss [53,59,94]. Aerogel production commonly involves sol–gel polymerization, aging, solvent exchange, and drying, all of which influence pore architecture and final properties while also contributing to environmental burden. Therefore, the sustainability of thermoset aerogels cannot be assessed solely by record properties such as low density, low thermal conductivity, or high compressive strength. It must be evaluated according to functional performance delivered per unit environmental burden over the full life cycle.

The comparison with biomass-derived aerogels clarifies the role of thermoset systems in this review. Biomass aerogels address the feedstock side of sustainability by introducing renewable or waste-derived carbon, but their advantages may be weakened by chemical modification, hydrophobization, solvent exchange, freeze-drying, moisture sensitivity, and limited durability under demanding conditions. Thermoset aerogels face the opposite challenge: they provide high durability and service performance, but their fossil-derived precursors, permanent crosslinking, and limited recyclability constrain circular material flows. These two material families therefore define complementary sustainability challenges. Biomass-derived aerogels must improve durability and processing efficiency, whereas thermoset aerogels must improve repairability, recyclability, and end-of-life recovery without sacrificing performance.

Recent scale-up fabrication of recycled PET aerogels has shown that this waste-derived platform can be translated beyond laboratory-scale preparation while retaining relevant thermal, acoustic, mechanical, and oil-absorption performance [106]. Sub-ambient radiative-cooling PET aerogels further extend recycled PET aerogels toward passive thermal-management applications [107]. In parallel, lightweight carbon nanotube aerogel composites and multifunctional PET aerogel-inspired materials broaden the application space toward electromagnetic wave absorption and construction-related uses [100,108]. However, recycled feedstock use should not be confused with closed-loop network circularity. rPET aerogels, PET–silica hybrid aerogels, polypropylene-fiber aerogels, and related waste-derived systems demonstrate valuable upcycling routes, but they do not automatically guarantee that the porous network can be repaired, reprocessed, regenerated, or recovered with high value retention. This distinction is particularly important for thermoset-based materials, where permanent covalent networks restrict molecular reconfiguration after curing. Sustainable end-of-life strategies for thermoset-based composites therefore increasingly emphasize chemical recycling, upcycling, fiber or matrix recovery, and value-retaining reuse pathways rather than simple disposal or low-value mechanical recycling [109].

Accordingly, thermoset aerogels should be understood as high-performance but largely non-circular networks that establish a baseline for next-generation sustainable aerogels. Their mechanical strength, thermal stability, and chemical resistance define the performance targets that dynamic and recycled thermoset aerogels should retain. At the same time, their poor reprocessability and limited end-of-life recovery define the circularity gaps that must be addressed through adaptive network chemistry, dynamic covalent bonds, chemically recyclable thermosets, waste-derived precursors, and aerogel-to-sol-to-aerogel regeneration. This transition from static permanent networks to adaptive, repairable, and recyclable porous systems forms the basis of the following subsections.

### 4.2. Network Chemistry: From Permanent Crosslinks to Dynamic Bonds

Network chemistry is the central design variable that distinguishes thermoset aerogels from biomass-derived aerogels. In biomass-derived systems, structural formation is often governed by hydrogen bonding, ionic coordination, electrostatic interactions, crystallinity, π–π stacking, and limited covalent stabilization. These interactions provide renewable-carbon pathways and structural versatility, but they can also introduce variability in gelation behavior, moisture sensitivity, and limited long-term dimensional stability. In thermoset aerogels, by contrast, covalent crosslinking fixes the polymer framework and provides high thermal resistance, chemical stability, and mechanical robustness. This transition from reversible or semi-reversible interactions to covalent networks links Section 4 directly to the multiscale framework in Section 2, because molecular connectivity controls gelation, network topology, pore preservation, and macroscopic performance [24,36,39].

At the molecular level, thermoset aerogel performance is governed by crosslink type, crosslink density, and network topology. Higher crosslink density generally improves stiffness, solvent resistance, thermal stability, and resistance to drying-induced collapse, but it also suppresses chain mobility and limits fracture tolerance. Lower crosslink density or more flexible segments may improve deformation resistance and stress dissipation, but often at the expense of dimensional stability and thermal robustness. Thus, the chemistry of crosslinking defines a bounded design space in which mechanical strength, thermal insulation, processability, and circularity cannot be optimized independently [24,96]. In cleaner-production terms, network chemistry is important not only because it controls performance, but also because it determines whether the aerogel can be repaired, reprocessed, recycled, or regenerated after use.

Conventional thermoset aerogels are based on irreversible covalent networks formed through condensation, imidization, ring-opening, addition, or step-growth reactions. Representative examples include phenolic and resorcinol–formaldehyde aerogels, polyimide aerogels, polyurethane and polyurea aerogels, and epoxy-based aerogels. These systems differ in precursor chemistry and processing route, but they share a common feature: once gelation and curing are completed, the network topology becomes permanently fixed. This fixed connectivity explains their high thermal, chemical, and mechanical stability, but it also prevents remelting, reshaping, welding, and efficient reprocessing [51,61,71,72,79,80,101]. Conventional thermoset networks can therefore serve as performance benchmarks, but not as circular material solutions.

This limitation motivates the development of dynamic covalent and vitrimer-like thermoset aerogels. In these systems, exchangeable covalent bonds allow network topology to rearrange under defined stimuli while retaining thermoset-like connectivity during service. Associative or reversible bond-exchange chemistries, including transesterification, imine exchange, boronic ester exchange, disulfide exchange, vinylogous urethane exchange, and siloxane exchange, can introduce stress relaxation, self-healing, repairability, reshaping, and reprocessing [69,76,96,110]. In aerogels, such dynamic chemistry is particularly attractive because local bond exchange may help dissipate internal stress during gelation, aging, solvent exchange, and drying, thereby reducing shrinkage, cracking, and pore collapse. At end-of-life, the same dynamic bonds may enable depolymerization, re-gelation, or aerogel-to-sol-to-aerogel regeneration.

Among dynamic systems, polyimine and vitrimer-like aerogels illustrate how network chemistry can move thermoset aerogels toward circularity. Polyimine aerogels have demonstrated closed-loop recyclability while retaining high-performance characteristics, showing that reversible imine chemistry can support network regeneration under controlled conditions [92]. Similarly, aerogel-to-sol-to-aerogel strategies show that organic aerogels can be recycled, repaired, and reprogrammed through a sol-state intermediate rather than simply downcycled [111]. Their significance lies in preserving or reconstructing the porous architecture, rather than merely recovering polymer mass. Broader validation is still needed across larger samples, repeated cycles, mixed waste streams, and realistic service environments.

Dynamic covalent chemistry also introduces a stability–adaptability trade-off. Faster bond exchange can improve stress relaxation, self-healing, and reprocessing, but may increase creep, reduce modulus, or compromise dimensional stability under long-term loading. Slower exchange preserves service stability but may require higher temperature, longer treatment time, catalysts, or solvent exposure during recycling. These conditions can increase energy demand and reduce the net sustainability benefit of dynamic networks, especially because aerogel fabrication already involves solvent exchange and drying steps that can dominate environmental impacts [53,76,94,97]. Therefore, dynamic thermoset aerogels should not be considered automatically sustainable; their circularity must be quantified through recovery yield, property retention, solvent recovery, reprocessing energy, cycle durability, and end-of-life value retention.

For thermoset-based materials, this distinction is particularly important. A recycled feedstock can reduce virgin-resource demand, but if the resulting network is permanently crosslinked and cannot be repaired, reprocessed, or recovered with high value retention, the material may still follow a largely linear end-of-life pathway. Sustainable end-of-life strategies for thermoset-based composites therefore increasingly emphasize chemical recycling, matrix recovery, fiber recovery, upcycling, and value-retaining reuse routes rather than simple disposal or low-value mechanical recycling [109]. Accordingly, the most promising thermoset and recycled thermoset aerogels are not those that optimize only one sustainability dimension, but those in which precursor origin, crosslink chemistry, pore architecture, processing intensity, service durability, and end-of-life pathway are designed together.

**Figure 4:**
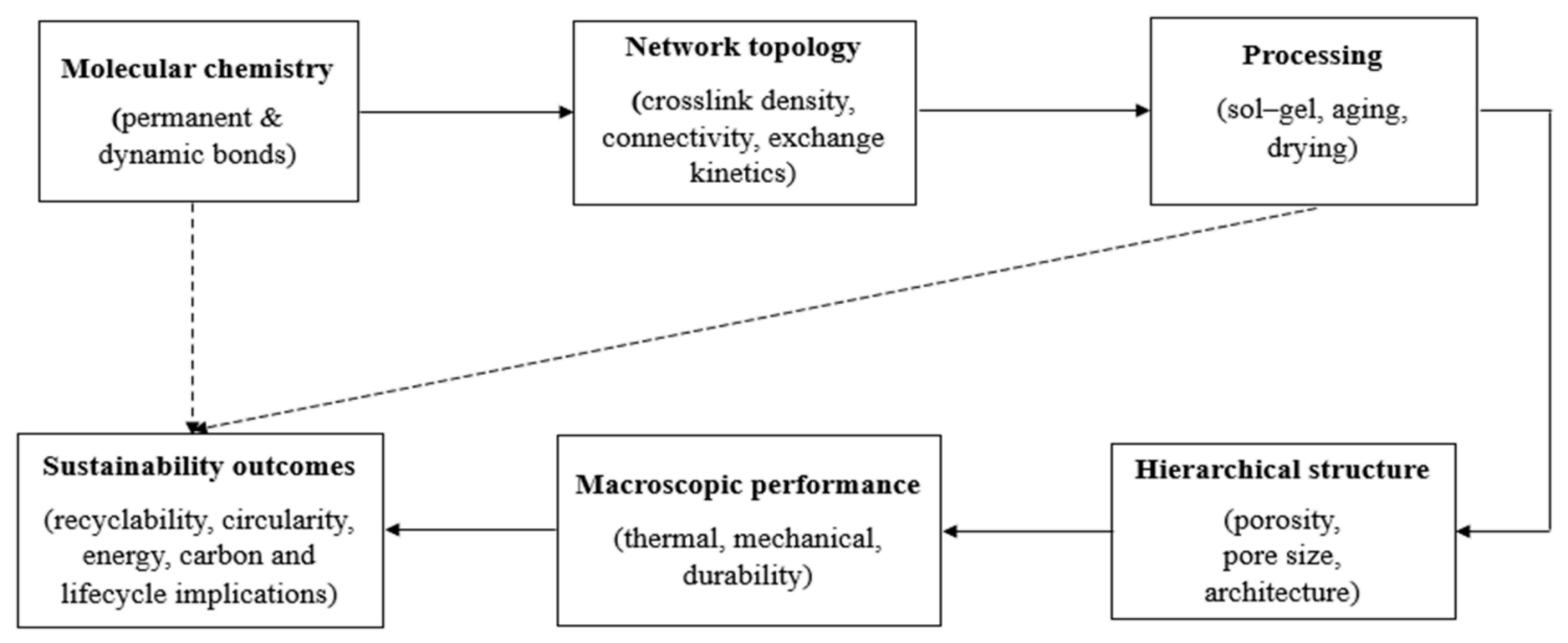
Multiscale design framework linking molecular chemistry, network topology, processing, hierarchical structure, macroscopic performance, and sustainability outcomes in thermoset-based and recyclable thermoset aerogels. Solid arrows indicate the primary structure–processing–property relationships, whereas dashed arrows represent direct influences of molecular chemistry and processing parameters on sustainability outcomes.

Figure 4 summarizes this multiscale relationship between molecular chemistry, network topology, processing, hierarchical structure, macroscopic performance, and sustainability outcomes. Permanent and dynamic covalent bonds define crosslink density, connectivity, and exchange kinetics, the same design levers that give unconventional polymer networks their extreme property combinations [112]; these molecular features interact with sol–gel formation, aging, solvent exchange, and drying to determine pore architecture. The resulting structure controls thermal insulation, mechanical robustness, and durability, while sustainability outcomes depend on recyclability, circularity, energy demand, carbon implications, and life-cycle performance. This framework leads into the following sections, which examine how formation pathways, reinforced architectures, and performance–recyclability trade-offs shape the practical sustainability of thermoset-based and recycled thermoset aerogels.

### 4.3. Formation and Architecture of Thermoset Aerogels

While Section 4.2 emphasizes how permanent and dynamic bonds define network chemistry, the final properties of thermoset aerogels are determined equally by how these networks form and how their hierarchical architectures are preserved during processing. Similar to biomass-derived aerogels discussed in Section 3, thermoset aerogels are not equilibrium materials. Their porous structures are kinetically encoded through sol–gel polymerization, curing, phase separation, aging, solvent exchange, and drying. However, thermoset systems differ from biomass-derived networks because covalent reactions progressively fix the network topology during formation. This fixation improves dimensional stability and mechanical robustness, but it also increases sensitivity to reaction rate, curing sequence, internal stress, and capillary-induced shrinkage.

In thermoset aerogels, architecture is therefore not a passive consequence of chemistry. It results from the coupling between molecular reactions, mesoscale phase evolution, and processing history. Parameters such as precursor functionality, catalyst concentration, stoichiometry, solvent quality, gelation temperature, aging time, and drying route determine pore size, pore connectivity, shrinkage, and load-bearing pathways [24,53,61]. This processing–structure relationship directly links Section 4 to the universal formation framework developed in Section 2, where aerogel morphology is governed by nonequilibrium gelation, phase separation, kinetic arrest, coarsening, and drying. It also supports the cleaner-production focus of this review because the same processing steps that preserve porosity, especially solvent exchange and drying, can contribute substantially to solvent use, energy demand, and scale-up difficulty [53,94].

Conventional thermoset aerogels illustrate this coupling clearly. Phenolic and resorcinol–formaldehyde aerogels are commonly formed through polycondensation reactions that generate aromatic crosslinked clusters, which then aggregate into a space-spanning porous network before aging and drying [61,101]. Their final microstructure depends strongly on precursor concentration, catalyst ratio, pH, solvent composition, and aging conditions. Insufficient neck growth between clusters can produce weak and brittle networks, whereas excessive coarsening or densification can increase thermal conductivity and reduce porosity. Recent phenolic aerogel designs have therefore focused on controlling gelation kinetics, shrinkage, and interparticle connectivity to improve compressive strength while maintaining low thermal conductivity [95,101,113,114].

Polyimide aerogels follow a more molecularly programmable formation pathway. They are usually prepared through polyamic acid formation followed by chemical or thermal imidization, with network continuity controlled by chain growth, crosslinker functionality, backbone rigidity, and segmental flexibility [72,103]. Compared with cluster-based phenolic or resorcinol–formaldehyde systems, polyimide aerogels can provide more continuous load-transfer pathways and improved mechanical resilience, especially when reinforced by nanofibers, aramid networks, lamellar architectures, or inorganic phases [66,105,115]. Nevertheless, they remain sensitive to drying-induced shrinkage and pore collapse, reinforcing the need to control polymerization, gelation, phase separation, and drying as a coupled process rather than as isolated synthesis steps.

Drying is a critical stage for all thermoset aerogels because the wet gel must be converted into a dry porous solid without destroying the network. Supercritical drying can minimize capillary stress but requires specialized equipment and high energy input. Freeze-drying can preserve certain anisotropic or lamellar structures but may introduce ice-templated morphology and processing constraints. Ambient pressure drying is more scalable but requires sufficient network stiffness or surface modification to resist capillary collapse [53,72,94]. Dynamic covalent thermoset aerogels add another dimension to this problem because bond exchange may relax internal stress during gelation, aging, solvent exchange, and drying. However, if exchange kinetics are too fast, the same mobility may promote creep, shrinkage, or loss of structural fidelity. Formation pathways for recyclable thermoset aerogels must therefore balance curing kinetics, phase separation, bond-exchange dynamics, and drying stress.

Hybridization and reinforcement provide additional routes to preserve architecture and expand functionality. In thermoset aerogels, reinforcing phases can redistribute load, bridge cracks, reduce shrinkage, improve thermal stability, and introduce functions such as flame resistance, electromagnetic absorption, or radiative cooling. Phenolic, polyimide, epoxy, polyurethane, and polyurea aerogels can be reinforced with inorganic fillers, ceramic phases, aramid nanofibers, nanocellulose, silica, alumina, graphene-based materials, carbon nanotubes, or other carbonaceous networks; double-layered polyimide/alumina composite aerogels illustrate how such hybridization can be organized spatially rather than uniformly [115]. Comparable optimization of silicon-based aerogel insulation illustrates the same trade-offs at the materials-selection level [116]. These strategies improve stiffness, toughness, fatigue resistance, and multifunctionality, but they also introduce new challenges related to filler dispersion, interfacial compatibility, processing reproducibility, and end-of-life separation.

Conductive and carbonaceous aerogel composites further broaden the architectural design space. Lightweight carbon nanotube aerogel composites, for example, demonstrate how nanoscale conductive networks can be integrated into porous frameworks to achieve electromagnetic wave absorption while retaining low density [108]. Such systems show that aerogel architectures can be designed for multifunctionality beyond thermal insulation, but they also complicate sustainability assessment because multicomponent structures may increase processing intensity and make end-of-life separation more difficult.

Overall, the formation and architecture of thermoset-based and recycled thermoset aerogels should be evaluated through the same service-based cleaner-production lens used throughout this review. Processing routes must preserve hierarchical porosity and mechanical integrity while reducing solvent exchange, drying energy, shrinkage, and material loss. Reinforcement and hybridization can improve durability and multifunctionality, but they should not be treated as inherently sustainable unless recyclability, property retention, and end-of-life value recovery are demonstrated. For thermoset-based reinforced systems, this is particularly important because chemically fixed matrices and embedded reinforcement phases often require tailored recycling or upcycling pathways [109]. The most promising architectures are therefore those in which precursor origin, pore hierarchy, reinforcement strategy, processing route, and end-of-life pathway are co-designed rather than optimized separately.

### 4.4. Performance–Recyclability Trade-Offs

The preceding sections show that thermoset aerogels achieve high performance through chemically fixed networks and carefully preserved hierarchical porosity. However, the same molecular and architectural features that improve thermal stability, mechanical robustness, and long-term durability often reduce reprocessability, recyclability, and end-of-life value retention. This trade-off differs from that of biomass-derived aerogels discussed in Section 3. Biomass-derived systems are often limited by moisture sensitivity, feedstock heterogeneity, and weak long-term dimensional stability, whereas thermoset aerogels face the opposite challenge: strong covalent fixation improves service performance but restricts circularity. Therefore, thermoset-based and recycled thermoset aerogels should be evaluated not only by density, thermal conductivity, compressive strength, or modulus, but also by whether these properties can be retained after repair, reuse, reprocessing, or recycling.

Thermal and mechanical stability remain the main technological advantages of thermoset aerogels. Their low thermal conductivity arises from high porosity, nanoscale pore confinement, reduced gas-phase conduction, and limited solid-phase heat transfer through the sparse polymer skeleton. Their mechanical stability depends on crosslink density, pore connectivity, strut thickness, interparticle necking, and reinforcement. Phenolic and resorcinol–formaldehyde aerogels provide thermal stability and char-forming ability, but their cluster-based structures can be brittle if interparticle junctions are weak or shrinkage is not controlled [78,95,101,113,114]. Polyimide aerogels offer higher thermal and mechanical resilience, especially when backbone flexibility, urea-containing linkages, nanofibrous reinforcement, or lamellar architectures improve load transfer and fatigue resistance [66,102,103,104,105]. Epoxy, polyurethane, and polyurea aerogels further show that curing chemistry and hard–soft segment organization can tune stiffness and toughness, although their irreversible covalent networks still prevent remelting and efficient reshaping [51,79,80].

From a cleaner-production perspective, high service performance is beneficial only when it offsets production and end-of-life burdens. A thermoset aerogel may reduce operational energy use in insulation or thermal protection applications, but its overall sustainability can be weakened by intensive solvent exchange, drying energy, poor damage tolerance, or lack of high-value recovery routes [53,59,94]. This means that thermal conductivity, compressive modulus, and durability should be interpreted together with processing energy, solvent recovery, use lifetime, repairability, recyclability, and property retention. In this sense, performance and recyclability are not separate criteria; they are coupled outcomes of network chemistry, pore architecture, processing intensity, and life-cycle pathway.

Dynamic covalent and vitrimer-like thermoset aerogels partially address this limitation by introducing exchangeable bonds that allow topology rearrangement under controlled conditions. These networks can enable stress relaxation, self-healing, repair, welding, reshaping, or chemical regeneration while maintaining thermoset-like connectivity during use [69,76,96,109]. However, reprocessability introduces a new stability–adaptability trade-off. Fast bond exchange can improve repair and recycling but may increase creep, pore deformation, or dimensional instability under long-term loading. Slower exchange can preserve service stability but may require higher temperature, longer processing time, catalysts, or solvent exposure during recycling, thereby increasing environmental burden [76,96,97]. Therefore, recyclable thermoset aerogels should not be judged only by whether they can be reprocessed once, but by how well they retain density, pore architecture, mechanical integrity, and thermal insulation across multiple cycles.

Property retention is therefore more informative than recyclability alone as a circularity metric. The polyimine and aerogel-to-sol-to-aerogel systems introduced in Section 4.2 illustrate the point: what distinguishes them is that they preserve or rebuild the porous architecture rather than merely recovering polymer mass. Broader validation is still needed across larger sample sizes, more recycling cycles, mixed waste streams, and realistic humidity, loading, and thermal environments.

Multifunctional aerogels introduce another aspect of this trade-off. As noted in Section 4.3, such systems may deliver higher service value per unit material, but conductive fillers, carbon nanomaterials, hybrid interfaces and multicomponent structures also complicate recycling and end-of-life separation. For thermoset-based reinforced systems, similar challenges arise because chemically fixed matrices and embedded fibers or fillers often require tailored recycling, matrix recovery, fiber recovery, or upcycling pathways [109].

Accordingly, performance–recyclability trade-offs in thermoset-based and recycled thermoset aerogels should be assessed using service-based and life-cycle-relevant metrics rather than isolated material properties. Key metrics should include thermal resistance over lifetime, mechanical property retention after cycling, creep resistance, recovery yield, solvent recovery, reprocessing energy, number of reuse cycles, regenerated aerogel quality, and end-of-life value retention. The most promising systems will be those that combine high thermal and mechanical performance with scalable processing, reproducible architecture, low environmental burden, and validated recovery pathways. This perspective provides the basis for linking Section 4 to the later discussion of structure–property–performance maps, life-cycle assessment, and cleaner-production roadmaps.

### 4.5. Recycling Strategies and Circular Design Limits

The performance–recyclability trade-offs discussed in Section 4.4 lead directly to the question of how thermoset-based aerogels can be recovered at the end-of-life. Unlike biomass-derived aerogels, whose end-of-life pathways may involve biodegradation, composting, carbonization, or renewable-carbon valorization depending on composition and processing history, thermoset aerogels face a more fundamental circularity barrier: their covalently fixed networks cannot be remelted or reshaped through conventional polymer recycling routes. Recycling strategies for thermoset aerogels therefore differ not only in processing method, but also in the degree to which they preserve molecular value, porous architecture, and functional performance.

Broadly, current pathways can be grouped into open-loop recycling, carbonaceous upcycling, dynamic network reprocessing, chemical recycling, and aerogel regeneration. Open-loop recycling converts thermoset or polymer aerogel waste into fillers, secondary composites, or lower-value porous products, but usually sacrifices the original aerogel architecture. Carbonaceous upcycling through pyrolysis or carbonization can generate conductive, thermally stable, or adsorption-active porous materials, but it destroys the original polymer network and may require high energy input. Dynamic covalent and vitrimer-like networks provide a more value-retaining route because reshaping, welding, self-healing, and reprocessing may extend service lifetime before final recycling [69,76,96]. Closed-loop chemical recycling and aerogel-to-sol-to-aerogel regeneration are the most circular options because they aim to recover molecular, structural, or functional value rather than simply divert waste from disposal [4,92].

The feedstock-versus-product distinction discussed in Section 4.2 is especially relevant to reinforced and hybrid systems, in which silica, fly ash, fibers, carbon nanotubes, inorganic fillers or chemically fixed matrices improve service performance but complicate separation and recovery after use [109].

The circular life-cycle of thermoset polymer aerogels can be viewed as a sequence of linked stages: synthesis, application, repair or reprocessing, recycling, and regeneration. Dynamic covalent or vitrimer-like networks can extend the use phase through reshaping, welding, and self-healing, while closed-loop recycling and aerogel-to-sol-to-aerogel regeneration can return materials to a renewed porous form. In contrast, open-loop recycling and carbonaceous upcycling may still be useful but generally involve value loss or structural transformation. Circularity is therefore not determined by a single material label such as “bio-based,” “recycled,” or “recyclable,” but by measurable retention of function and value across the life-cycle.

Circular design limits should accordingly be defined quantitatively, using the service-based and life-cycle-relevant metric set already specified in Section 4.4 rather than a separate list. This framing links Section 4 to the structure–property–performance maps, life-cycle assessment and cleaner-production roadmap developed in the sections that follow.

**Figure 5:**
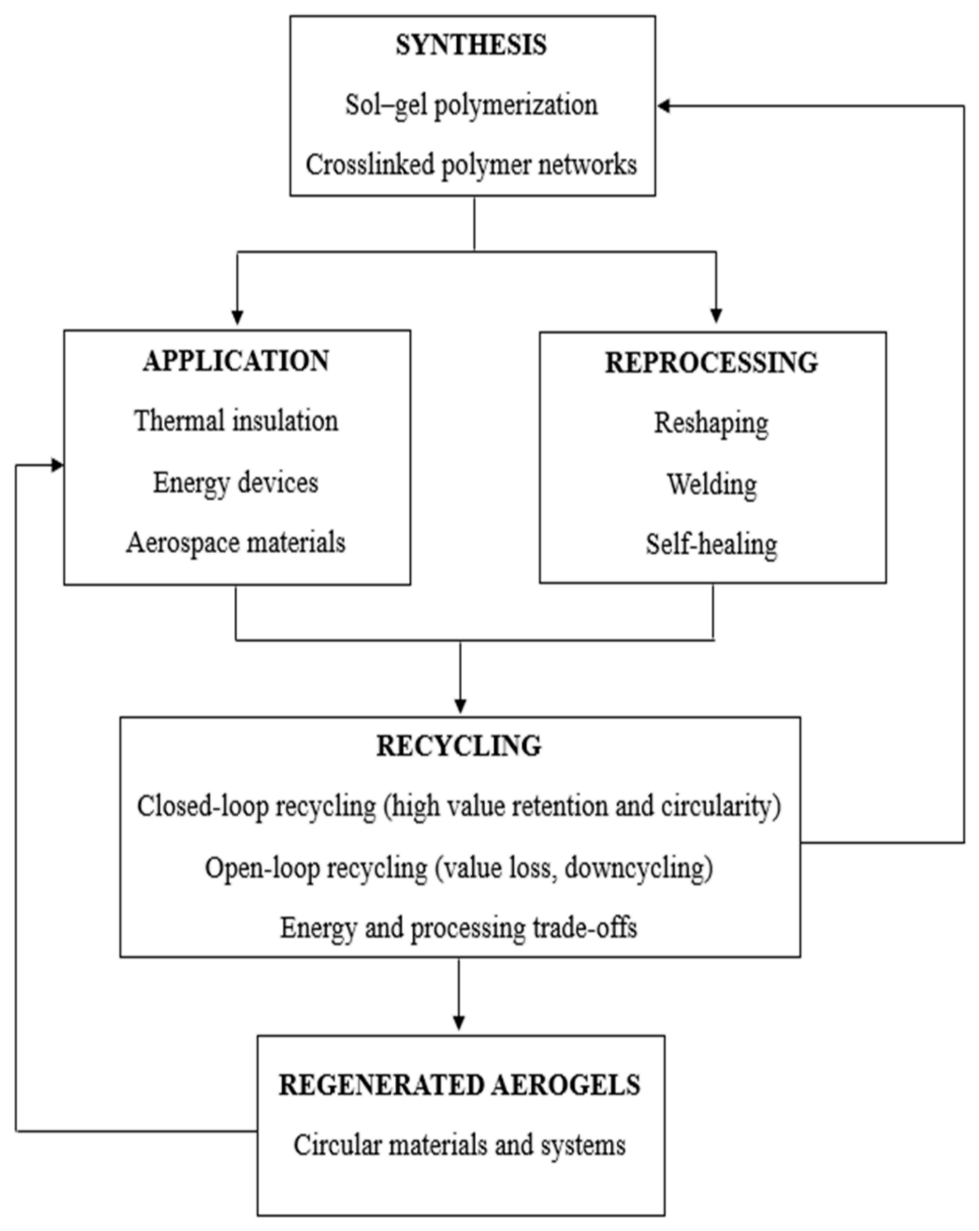
Circular life-cycle of thermoset polymer aerogels.

The circular life-cycle framework of thermoset polymer aerogels is illustrated in Figure 5, highlighting the relationship between material design, use phase, recovery strategies, and end-of-life pathways.

The figure maps the stages described above onto a closed loop, distinguishing value-retaining closed-loop routes from value-losing open-loop routes.

### 4.6. Design Guidelines and Outlook for Circular Thermoset Aerogels

The discussion above shows that thermoset-based and recycled thermoset aerogels should not be designed only for high thermal or mechanical performance. Their sustainability depends on the co-design of precursor origin, network chemistry, pore architecture, processing intensity, service durability, and end-of-life recovery. Conventional thermoset aerogels provide important performance benchmarks, but their permanent covalent networks restrict repair, reprocessing, and closed-loop recovery. Dynamic covalent and vitrimer-like systems address this limitation by introducing network adaptability, while waste-derived polymer aerogels improve feedstock circularity by converting post-consumer or secondary resources into value-added porous materials.

Several practical design guidelines follow. First, feedstock circularity and network circularity should be separated clearly. Recycled PET aerogels, functionalized PET aerogels, PET–silica hybrids, PET aerogel composites, polypropylene-fiber aerogels, and fly-ash/PET composite aerogels demonstrate effective waste valorization for thermal insulation, acoustic absorption, oil/water separation, oil-spill remediation, radiative cooling, and multifunctional building applications [100,106,107]. However, these systems should not automatically be considered closed-loop materials unless repairability, regeneration, recovery yield, and property retention are verified.

Second, dynamic network chemistry should be introduced only when it improves life-cycle performance and value retention. Exchangeable bonds can enable stress relaxation, self-healing, reshaping, reprocessing, and aerogel regeneration, but they may also reduce creep resistance, dimensional stability, or long-term durability if bond exchange is too fast. Therefore, dynamic thermoset aerogels should be evaluated by both recyclability and service stability, including thermal-conductivity retention, pore-structure retention, mechanical-property retention, and performance after repeated cycles [4,69,76,92,96].

Third, reinforcement and hybridization should be designed for both performance and recoverability. Fibers, silica, fly ash, carbon nanotubes, inorganic fillers, and carbonaceous networks can improve toughness, dimensional stability, radiative cooling, electromagnetic wave absorption, or multifunctionality, as shown by recycled PET composites and carbon nanotube aerogel composites [99,100,106,107,108]. However, multicomponent architectures may complicate separation and end-of-life recovery. For thermoset-based reinforced systems, future designs should consider matrix recovery, fiber recovery, chemical recycling, and upcycling from the beginning rather than treating end-of-life management as an afterthought [109].

Fourth, processing intensity should be treated as a sustainability variable rather than merely as a technical detail. Sol–gel formation, curing, aging, solvent exchange, drying, reprocessing, and regeneration determine pore architecture and final performance, but they also contribute to embodied energy, solvent use, emissions, safety concerns, and scale-up difficulty. Low-energy drying, solvent recovery, ambient-pressure processing, water-compatible chemistry, and scalable fabrication should therefore become core design criteria for thermoset and recycled thermoset aerogels, especially when circularity claims depend on repeated regeneration or reprocessing.

Overall, Section 4 establishes thermoset-based and recycled thermoset aerogels as a bridge between high-performance porous polymers and circular material design. Conventional thermoset aerogels define the performance targets: low density, thermal insulation, mechanical robustness, chemical stability, and dimensional integrity. Dynamic and recyclable thermoset aerogels define the network-circularity targets: repairability, reprocessability, closed-loop recovery, and aerogel regeneration. Waste-derived polymer aerogels define the feedstock-circularity target: converting plastic and composite waste into value-added porous architectures. The next challenge is to integrate these objectives into a unified design strategy in which performance and sustainability are optimized together from the molecular stage.

These conclusions provide the transition to the following sections of the review. Section 5 translates the qualitative principles discussed here into structure–property–performance maps, enabling comparison across biomass-derived, thermoset, recycled, dynamic, and hybrid aerogels. Section 6 extends this framework through multiscale modeling and AI-guided design, where network chemistry, pore architecture, processing metadata, and environmental descriptors can be treated as coupled design variables. Section 7 and Section 8 then evaluate application performance and life-cycle outcomes through functional units, circularity metrics, and cleaner-production criteria. In this broader context, thermoset and recycled thermoset aerogels should be evaluated not only as high-performance materials, but as integrated life-cycle systems whose value depends on molecular design, porous architecture, processing route, service lifetime, and end-of-life recovery.

## 5. Structure–Property–Performance Maps

The preceding sections show that sustainable polymer aerogels cover a broad range of chemical compositions and architectures, ranging from biomass-derived networks such as cellulose, chitosan, lignin, alginate, and protein aerogels to high-performance thermoset, vitrimer, and hybrid systems. However, qualitative classification alone is insufficient for rational materials selection. Because aerogel performance is governed by the coupled effects of density, porosity, pore size distribution, connectivity, surface chemistry, and network topology, a structured mapping framework is required to translate structural descriptors into application-relevant performance metrics. Structure–property–performance maps therefore provide a link between materials chemistry and predictive design. They organize representative values reported for biopolymer-derived, thermoset-based, recycled thermoset, and hybrid aerogels within a common conceptual design space, supporting qualitative comparison of performance trade-offs and highlighting sparsely populated regions that represent future design targets.

Ashby-type maps are particularly useful for aerogels because many key performance metrics are intrinsically coupled. Lower density generally benefits lightweight design and thermal insulation, but excessive porosity can compromise mechanical integrity. Higher surface area favors adsorption, catalysis, and electrochemical storage, but may not guarantee fast mass transport if pore accessibility and connectivity are poor. Similarly, increasing crosslink density can enhance stiffness and dimensional stability, but may reduce toughness, flexibility, or recyclability. Thus, rather than treating thermal, mechanical, transport, and sustainability metrics independently, structure–property–performance maps provide a multidimensional framework for identifying Pareto-optimal aerogel designs.

### 5.1. Building a Unified Polymer Aerogel Database

A prerequisite for meaningful structure–property mapping is the construction of a curated polymer aerogel database. Such a database should integrate data from both conventional experimental reports and emerging machine-readable sources, with explicit attention to composition, processing history, structural descriptors, and measured properties. At minimum, each entry should include: precursor type, renewable or recycled content, crosslinking chemistry, solvent system, gelation route, aging conditions, solvent exchange protocol, drying method, bulk density, porosity, BET surface area, pore size distribution, thermal conductivity, compressive modulus, compressive strength, strain recovery, electrical conductivity when relevant, adsorption capacity, and cyclic stability. For sustainable polymer aerogels, additional descriptors should be incorporated, including biomass source, degree of functionalization, recyclability mechanism, degradation pathway, and estimated processing intensity.

The importance of database construction has already been demonstrated in the biopolymer aerogel field. Zhao et al. [117] compiled a database containing more than 3800 reported properties of biopolymer aerogels and foams, showing that the field is rich in experimental data but still dominated by empirical discovery rather than theory-guided design. More recently, data-driven reviews of aerogels have emphasized that the rapidly expanding literature contains both valuable structure–property information and substantial data-quality challenges, including inconsistent reporting, missing processing parameters, and unreliable or non-comparable property values. These challenges are especially severe for polymer aerogels because nominally similar materials may display very different performance depending on solvent exchange, drying, shrinkage, aging, testing direction, humidity, and sample geometry.

To reduce ambiguity, the database should distinguish between primary structural descriptors and derived performance indices. Primary descriptors include density, porosity, mean pore size, pore size distribution, surface area, ligament diameter, anisotropy, crosslink density, and functional group density. Derived indices can then be calculated to compare performance across material classes. Examples include specific modulus, specific compressive strength, thermal insulation index, adsorption efficiency per unit mass, electrical conductivity per density, and recyclability-adjusted performance. This distinction is important because a material with moderate absolute strength may become highly competitive when normalized by density, while a material with high adsorption capacity may be less attractive if it requires energy-intensive drying or toxic solvent processing.

Automated text-mining methods can accelerate this database construction. ChatExtract, a conversational large-language-model-based workflow for materials data extraction, has been shown to achieve close to 90% precision and recall for materials data extraction tasks, while reducing the need for extensive rule-based parser development. In the context of aerogels, similar NLP-assisted workflows could extract synthesis–structure–property triples such as “cellulose concentration–freeze-drying direction–thermal conductivity,” “polyimide crosslinker ratio–density–compressive modulus,” or “amine functionalization–CO_2_ uptake–regeneration stability.” Nevertheless, automated extraction should not replace expert curation. Aerogel datasets require manual validation of units, testing conditions, sample anisotropy, and whether reported values correspond to dry aerogels, wet gels, cryogels, xerogels, or composites. Without this normalization, Ashby maps may reflect reporting artifacts rather than true materials performance (Table 2).

### 5.2. Ashby-Type Property Maps for Polymer Aerogels

Ashby-type property maps provide a visual basis for comparing aerogels across composition, architecture, and function. For sustainable polymer aerogels, at least four maps are especially informative: thermal conductivity versus density, compressive modulus or strength versus density, BET surface area versus density, and functionality or sustainability-adjusted performance versus material class. These maps should be plotted on logarithmic axes when possible, because aerogel properties often span several orders of magnitude.

The first key map is thermal conductivity versus density. This chart directly evaluates the lightweight insulation performance of aerogels. In highly porous aerogels, thermal conductivity arises from the sum of solid conduction, gas conduction, convection, and radiative transfer. Reducing density decreases the solid conduction pathway, while reducing pore size below or near the mean free path of air suppresses gas-phase conduction through the Knudsen effect. However, extremely low density may lead to fragile, poorly connected networks, and large macropores can increase gas transport. Thus, the most attractive region of this map is not simply the lowest density region, but the zone where low density is combined with nanoscale pores, sufficient structural integrity, and stable morphology under humidity and compression.

Recent polymer aerogel fibers illustrate this coupling between nanoscale architecture and insulation performance. Gradient all-nanostructured aramid aerogel fibers achieved a radial thermal conductivity as low as 0.0228 W·m^−1^·K^−1^ [118], together with tensile strength of 29.5 MPa and fracture strain of 39.2%, showing that controlled radial nanostructure can simultaneously improve thermal resistance and mechanical robustness. Such results occupy a previously inaccessible region of the thermal conductivity–density map and signal a departure from the traditional trade-off between insulation and strength.

The second key map is compressive modulus or strength versus density. Classical cellular-solid theory predicts that mechanical properties scale with relative density according to power-law relationships as shown in Equation (1):

(1) E/Es = C(ρ/ρs)n

where Eis the aerogel modulus, Es is the modulus of the solid skeleton, ρ is the aerogel density, ρsis the solid density, and nis a scaling exponent that reflects deformation mode and network architecture. For ideal open-cell foams, n is often close to 2 for bending-dominated architectures, whereas stretching-dominated or highly aligned networks may show more favorable scaling. Real aerogels often deviate from ideal cellular-solid models because their networks are fractal, heterogeneous, anisotropic, and strongly influenced by drying-induced shrinkage and nanoscale connectivity. Therefore, plotting modulus or strength against density provides not only a performance comparison but also a diagnostic tool for identifying whether a given aerogel behaves as a bending-dominated, stretching-dominated, or fiber-reinforced network.

Hybrid fiber aerogels demonstrate the value of this map. A dual-scale micro-/nanofiber hybrid aerogel reported in Nature Communications showed high specific tensile modulus of approximately 1961.3 MPa cm^3^ g^−1^ and fracture energy of approximately 7448.8 J m^−2^, while retaining super-elastic recovery. Such systems occupy a different region from conventional brittle aerogels because stress is redistributed through physically entangled multiscale fiber networks rather than through sparse particulate necks. A useful classification therefore separates aerogels not only by chemistry but also by architecture—particulate, nanofibrillar, lamellar, aligned, gradient, or dual-scale fiber network.

The third key map is BET surface area versus density. This map is particularly relevant for adsorption, catalysis, carbon capture, and electrochemical applications. High surface area is often desirable, but its usefulness depends on accessibility, pore connectivity, and chemical functionality. For example, microporosity may increase surface area but limit diffusion of large molecules, whereas hierarchical meso/macroporosity can improve transport while maintaining sufficient active surface. Therefore, surface area–density maps should be interpreted alongside pore size distribution and functional group density. Biopolymer aerogels often provide abundant functional groups such as hydroxyl, carboxyl, amino, or phenolic moieties, whereas carbonized thermoset aerogels and graphene-based aerogels may provide electrical conductivity and high surface area but lower biodegradability.

The fourth recommended map is a sustainability-adjusted performance map. Conventional Ashby maps usually compare material properties, but sustainable aerogels require additional axes such as renewable content, recyclability, solvent intensity, embodied energy, or circularity potential. For example, a biopolymer aerogel with moderate mechanical performance may be favored for disposable biomedical or packaging applications because it is biodegradable and water-processable. Conversely, a thermoset aerogel may be justified in aerospace or high-temperature insulation if its lifetime performance offsets higher processing intensity. Recyclable thermoset aerogels are particularly important because they may reduce the historical conflict between covalent network stability and circularity. Recent work on polyhexahydrotriazine aerogels demonstrates that chemically robust organic aerogels can be designed for recyclability through dynamic network cleavage and monomer recovery.

### 5.3. Cross-Class Comparison: Biopolymer, Thermoset, Recycled Thermoset, and Hybrid Aerogels

When biopolymer, thermoset, recycled thermoset, and hybrid aerogels are plotted in the same property space, their complementary strengths become apparent. Biopolymer aerogels generally occupy the region associated with low density, high renewable content, rich surface functionality, and environmentally benign processing. Their advantages are most evident in adsorption, water treatment, biomedical scaffolds, wound dressings, packaging, and low-to-moderate temperature insulation. However, many biopolymer aerogels suffer from moisture sensitivity, limited thermal stability, and weaker mechanical performance unless reinforced by crosslinking, mineralization, carbonization, or hybridization.

Thermoset aerogels, including polyimide, polyurethane, polyurea, phenolic, epoxy, and related networks, typically occupy regions of higher thermal stability, stronger mechanical integrity, and better chemical resistance. Their dense covalent networks are advantageous for aerospace insulation, structural lightweight components, flame-resistant materials, and carbon aerogel precursors. However, the same covalent permanence that provides stability also creates sustainability limitations, particularly poor recyclability and difficult end-of-life management.

Recycled thermoset and dynamic covalent aerogels represent an emerging class that may occupy a previously underdeveloped region of the design space: mechanically robust, chemically stable, and recyclable porous networks. Vitrimer–graphene aerogel composites, for example, demonstrate how dynamic covalent polymer matrices can be integrated with conductive porous scaffolds to produce malleable and recyclable aerogel composites with electrical functionality. More recent recyclable polyhexahydrotriazine aerogels further suggest that circular thermoset aerogels are becoming experimentally feasible rather than merely conceptual [119].

Hybrid aerogels occupy the broadest and most tunable design region. By combining biopolymer backbones with thermoset, ceramic, carbonaceous, or nanofiber reinforcements, hybrid systems can decouple properties that are otherwise difficult to optimize simultaneously. For example, a cellulose or chitosan matrix may provide renewable functionality, while aramid nanofibers, silica, graphene, carbon nanotubes, or polyimide segments enhance mechanical stability, thermal resistance, or conductivity. In property maps, hybrid aerogels often appear as transition materials between the sustainability-rich but mechanically limited biopolymer region and the performance-rich but circularity-limited thermoset region. Table 3 summarizes this qualitative cross-class comparison, highlighting the complementary strengths, intrinsic limitations, and characteristic application domains of biopolymer, thermoset, recycled thermoset, and hybrid aerogel families.

### 5.4. Identifying Gaps and Design Targets

The main value of structure–property–performance maps lies not only in classifying existing aerogels but also in revealing unoccupied or sparsely populated regions of the design space. Several high-value gaps can be identified.

First, there remains a limited number of polymer aerogels that simultaneously achieve ultralow thermal conductivity, mechanical robustness, and circularity. Many high-performance thermoset aerogels are mechanically and thermally attractive but not recyclable. Conversely, many bio-based aerogels are sustainable in origin but do not yet match the mechanical or environmental durability of thermoset systems. A major design target is therefore a recyclable or bio-derived aerogel with thermal conductivity below approximately 0.025 W m^−1^ K^−1^, compressive strength in the MPa range, stable performance under humidity, and a validated end-of-life pathway.

Second, the low-density/high-strength region remains underexplored for fully sustainable aerogels. Fiber alignment, dual-scale networks, and gradient structures show that architecture can significantly shift mechanical scaling behavior. The next generation of biopolymer aerogels should therefore move beyond random freeze-dried networks toward programmed anisotropy, interpenetrating networks, and sacrificial bonding mechanisms. The goal is not merely to increase crosslink density, but to design energy-dissipating architectures that preserve elasticity and toughness.

Third, the high-surface-area/high-accessibility region remains challenging. Many aerogels report high BET surface area, but application performance depends on whether active sites are accessible under realistic conditions. For adsorption and catalysis, future maps should include not only BET surface area but also uptake kinetics, regeneration efficiency, selectivity, and performance retention over cycles. This is particularly important for CO_2_ capture and water remediation, where equilibrium capacity alone can overestimate practical performance.

Fourth, the conductive yet biodegradable or recyclable aerogel region remains largely open. Carbonized and graphene-based aerogels can provide excellent electrical conductivity for sensors, EMI shielding, supercapacitors, and batteries, but they are often difficult to recycle or biodegrade. Dynamic covalent matrices, bio-derived carbon precursors, and reversible polymer–carbon interfaces may provide routes toward circular conductive aerogels.

Finally, the maps reveal a methodological gap: most reported aerogel properties are not yet sufficiently standardized for reliable machine learning. Future reports should include complete processing metadata, replicate measurements, uncertainty ranges, testing direction, humidity, sample dimensions, and post-processing shrinkage. Without such information, ML models trained on literature data may learn reporting bias rather than true structure–property relationships. Structure–property–performance maps therefore function as both a design tool and a reporting framework.

Overall, this section establishes the conceptual foundation for the computational and AI-guided approaches discussed in the following sections. By organizing heterogeneous literature data into shared, literature-informed property spaces, these maps help identify performance trade-offs, cross-class synergies, and candidate regions for inverse design, while the quantitative benchmarking of those regions must await a curated database of the kind described above. In this sense, Ashby-type maps are no longer only visual tools for materials selection; they are also interpretable feature spaces for multiscale modeling, Bayesian optimization, active learning, and generative design of sustainable polymer aerogels.

**Figure 6:**
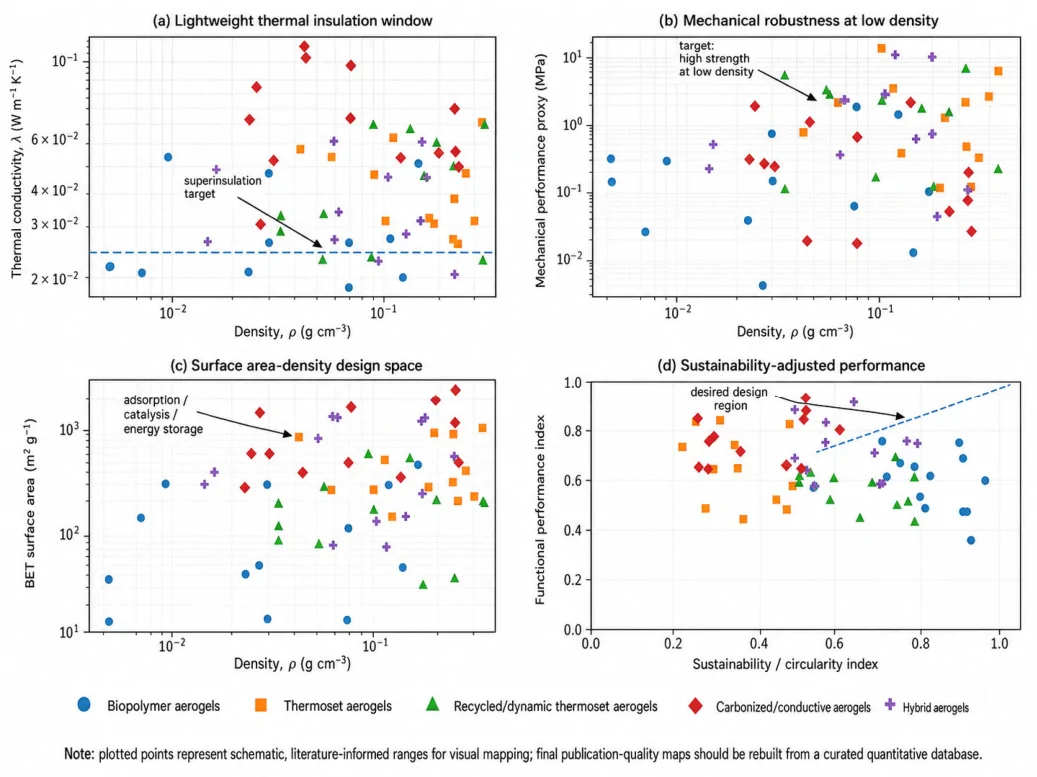
Structure–property–performance maps for sustainable polymer aerogels.

Figure 6 presents schematic Ashby-type maps comparing major sustainable polymer aerogel classes, including biopolymer aerogels, conventional thermoset aerogels, recycled/dynamic thermoset aerogels, carbonized/conductive aerogels, and hybrid aerogels. The panels illustrate representative design spaces for: (a) thermal conductivity versus density, (b) mechanical performance versus density, (c) BET surface area versus density, and (d) sustainability-adjusted functional performance.. The plotted regions are conceptual by design: they are literature-informed schematic ranges used to visualize qualitative trends and design gaps, not statistically validated property distributions. They were assembled from representative values reported in the cited primary literature without systematic inclusion criteria, normalization, or uncertainty quantification, and therefore must not be used for quantitative materials selection or benchmarking. A curated, FAIR-compliant quantitative database with explicit data sources, inclusion criteria, normalization procedures, and uncertainty estimates would be needed before such maps could support quantitative inverse design.

**Figure 7:**
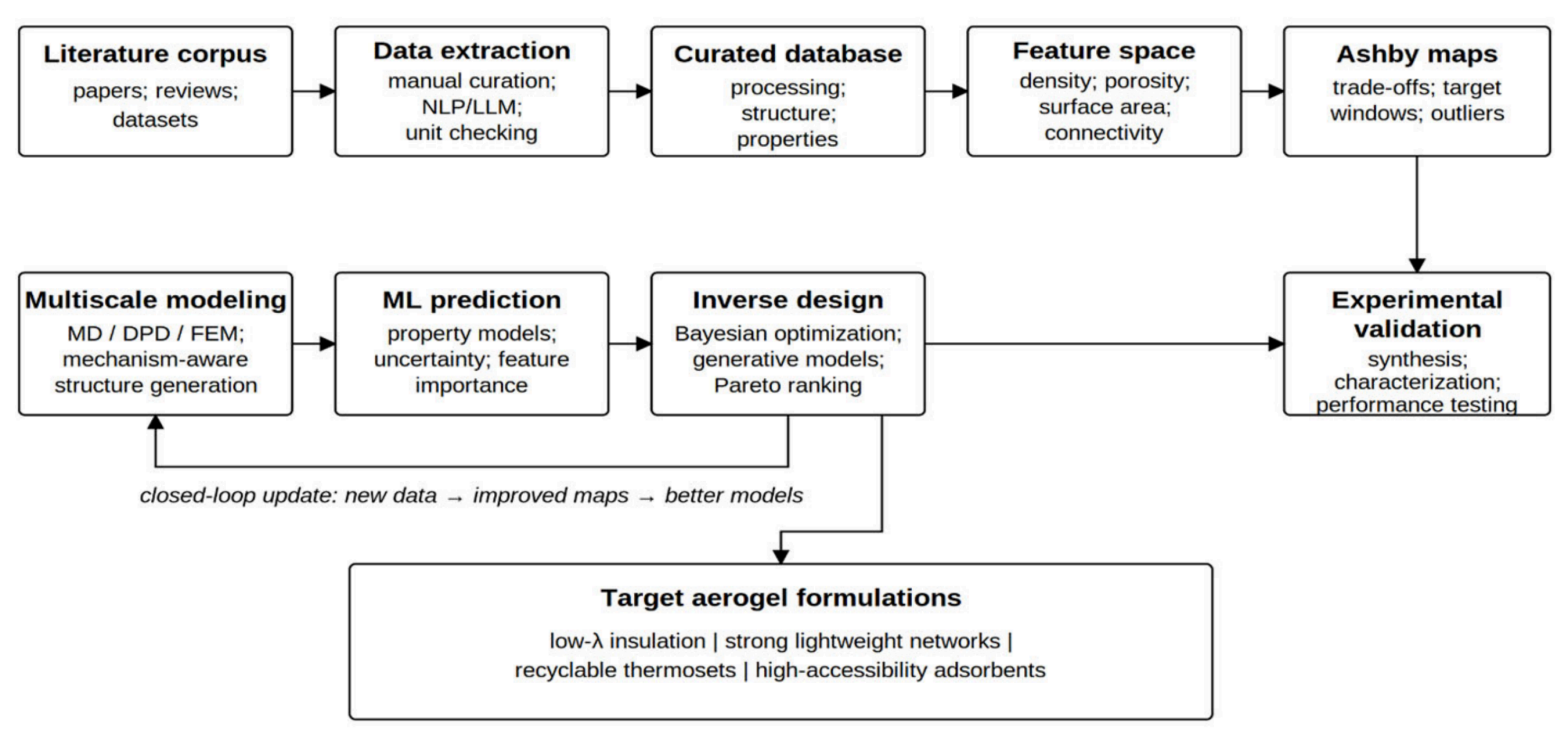
Workflow from structure–property maps to AI-guided aerogel design.

Figure 7 AI-guided framework for sustainable polymer aerogel design integrating literature mining, curated databases, structure–property feature spaces, Ashby-type maps, multiscale modeling, machine-learning prediction, inverse design, and experimental validation. The closed-loop framework enables newly measured aerogel data to update property maps and improve model accuracy, supporting rational discovery of low-thermal-conductivity insulation materials, mechanically resilient lightweight networks, recyclable thermoset aerogels, and high-accessibility adsorbents. Arrows indicate the sequential workflow, information transfer pathways, and the closed-loop feedback between data generation, model improvement, and experimental validation.

Although these structure–property–performance maps reveal where current aerogels cluster and which design regions remain unoccupied, they do not in themselves explain how molecular and processing levers translate into those macroscopic locations. Closing this gap requires predictive tools capable of connecting precursor chemistry, gelation kinetics, drying trajectory, and pore architecture to mapped performance and sustainability descriptors. Section 6 therefore introduces multiscale computational modeling and AI-guided design as tools for moving the maps in Section 5 from descriptive comparisons toward inverse-design targets, particularly when environmental descriptors are included as explicit objectives rather than post hoc justifications.

## 6. Multiscale Modeling and AI-Guided Sustainable Design

The sustainable design of polymer aerogels confronts what may be termed a sustainability paradox: the molecular and processing levers that historically delivered record thermal, mechanical, and adsorption performance—fossil-derived monomers, toxic solvents, supercritical drying, and permanent covalent crosslinks—are precisely those that compromise life-cycle sustainability, while greener alternatives based on biomass, mild aqueous processing, and dynamic networks frequently underperform on durability and load-bearing metrics. Conventional Edisonian trial-and-error optimization cannot resolve this paradox; on the contrary, it actively reinforces it, because each empirical iteration consumes solvents, drying energy, precursors, and time, and because the high-dimensional, multiscale coupling among precursor chemistry, gelation kinetics, drying route, and pore architecture produces a design space far too large for one-factor-at-a-time exploration. Digital transformation—encompassing multiscale physics-based modeling, machine learning, generative design, knowledge mining, and self-driving laboratories—offers a coherent pathway to reframe this paradox as a co-optimization problem in which functional performance and environmental burden are pursued simultaneously rather than sequentially. This section therefore consolidates computational and AI-guided strategies for polymer aerogels around a single question: how can digital approaches deliver Pareto-optimal aerogels in which sustainability is built in from the start, rather than retrofitted after performance has been maximized?

### 6.1. Multiscale Modeling as In Silico Replacement for Material-Intensive Screening

Multiscale computational modeling provides the physics-based foundation that allows synthesis–structure–property relationships to be probed without consuming experimental resources. A useful hierarchy can be considered at three levels: (i) atomistic molecular dynamics, which extracts intrinsic descriptors of binding, packing, interfacial affinity, fractal dimension, thermomechanical limits, and pyrolysis kinetics, and which has been applied directly to reinforced silica aerogel networks [120]; (ii) mesoscale microstructure reconstruction, which translates these descriptors into representative volume elements through aggregation algorithms such as diffusion-limited cluster–cluster aggregation, discrete element methods, or Voronoi tessellations [121,122,123]; and (iii) reduced-order network and continuum models, which upscale the resolved geometry to predict macroscopic mechanical, thermal, and transport behavior [12]. Across this pipeline, network models in particular abstract complex 3D microstructures into thermal-resistor or load-bearing graphs with embedded nanoscale corrections such as Knudsen-regime conductance, capturing percolation behavior at orders-of-magnitude lower computational cost than direct numerical simulation while remaining benchmarkable against finite-element and Lattice Boltzmann references [124,125].

For cleaner production, a key advantage of this hierarchy is that some material-intensive trial synthesis can be replaced by computational screening: solvent volumes, drying conditions, and crosslinker fractions can be screened across thousands of virtual configurations before any precursor is consumed. The same pipeline also enables digital twins for aerogel manufacturing—virtual replicas that ingest real-time temperature, pressure, and solvent-gradient signals during supercritical or freeze drying. To our knowledge, no peer-reviewed digital twin has yet been reported for aerogel production, but adjacent work in cellulose gelation modeling [68] and reconstruction-based heat-transfer prediction [126,127], together with image-based computation of effective thermal and mechanical properties of fibrous porous media [128,129], shows that the individual components are already well established. Combining them in a closed-loop process model could help reduce batch failures and the associated material and energy losses at pilot and industrial scales.

Beyond optimization, this multiscale hierarchy also delivers something machine learning alone cannot: causal interpretability. Because each tier is anchored in physical law, virtual experiments expose why specific solvent–crosslinker–drying combinations yield superior performance, providing the mechanistic basis on which the data-driven strategies discussed in Section 6.2 and Section 6.3 can be built without becoming black boxes.

### 6.2. Machine Learning for Sustainability-Constrained Inverse Design

Building on the physics-based foundation of Section 6.1, supervised machine learning has expanded from single-property prediction toward multi-objective inverse design that can incorporate environmental descriptors alongside performance targets. Forward models—spanning artificial neural networks, tree-based ensembles, Gaussian processes, and convolutional networks—now map synthesis and processing inputs to BET surface area, density, compressive modulus, and effective thermal conductivity for silica, polyimide, and MXene/cellulose aerogel systems [130,131,132]. Reported accuracies are, however, heterogeneous and not directly comparable across studies. Walker et al. [130] trained a neural-network regression on a curated silica-aerogel database of 103 entries and obtained an average error of 109 ± 84 m^2^ g^−1^ for BET surface area; an error below 5% was recovered not as a test-set statistic but in a single subsequent validation synthesis under new processing conditions. Tafreshi et al. [131] reported mean squared errors and Pearson correlation coefficients, rather than relative errors, for the compressive modulus, density and porosity of polyimide aerogels. Rong et al. [132] compared neural networks, support vector machines, and random forests across 34 Ti_3_C_2_ MXene/nanocellulose datasets and found neural networks most accurate, with the relative MXene content the dominant descriptor. No common error metric, validation protocol, or applicability domain is reported across these studies. For cleaner production, a main value of these forward models is their use as inexpensive surrogates within outer optimization loops.

These figures require careful interpretation. In particular, the sub-5% value should not be read as a general accuracy level for aerogel property prediction. It derives from agreement between prediction and measurement in one confirmatory synthesis, whereas the corresponding model error across the database was an order of magnitude larger in relative terms. More broadly, the accuracies reported in this literature describe interpolation within narrow, internally consistent design spaces built from small single-laboratory datasets, not extrapolative predictive power, and errors for thermal conductivity and mechanical properties are typically larger than for surface area or density. Given the dataset sizes discussed in Section 6.5, cross-validation folds are small, uncertainty is rarely quantified, and the reported accuracy is conditioned on the same reporting biases that affect the source literature. Accordingly, accuracy claims should be reported together with dataset size, feature space, validation protocol, and applicability domain before they are used to justify substituting model predictions for experiments.

Sustainability objectives can then be introduced in the outer optimization loop. Bayesian optimization, genetic algorithms (NSGA-II and its preference-vector variants), and deep reinforcement learning have each been deployed to navigate the conflict between minimizing thermal conductivity, maximizing compressive strength, controlling density, and—increasingly—minimizing aerogel dosage and embedded resource intensity. A representative 2025 Pareto-driven framework for aerogel-cement-EPS panels reported a Pareto front on which thermal conductivity was reduced to 0.0374 W m^−1^ K^−1^ (about 12% below comparable materials), while a separate Pareto-optimal point achieved an aerogel dosage of only 6.8 kg m^−3^ at a thermal conductivity of 0.0397 W m^−1^ K^−1^ [133], showing that performance and material use can be optimized together. Multi-information Bayesian frameworks have, in parallel, been shown to identify Pareto-optimal compositions substantially more efficiently than brute-force search, first for multi-objective design under active constraint learning [134] and subsequently using an entropy-based formulation that recovered 21 Pareto-optimal alloys satisfying all imposed constraints [135]. Because these demonstrations concern alloy systems, any corresponding reduction in solvent, precursor, and energy use for aerogel discovery remains to be demonstrated.

Crucially, none of these methodological gains delivers genuine sustainability benefits unless environmental descriptors—solvent intensity, drying energy, renewable-carbon fraction, regeneration cycles, and end-of-life pathway—are entered as explicit objectives or constraints. Where such descriptors are absent, machine learning simply rediscovers the existing paradox at greater speed. This limitation is not algorithmic but informational and motivates the knowledge-mining and generative strategies discussed in Section 6.3, which seek to expand both the diversity and the sustainability metadata of the underlying training corpus.

A practical four-objective workflow that treats sustainability as an optimization target rather than a separate assessment could proceed as follows. Design variables would comprise precursor concentration, crosslinker fraction, solvent-exchange schedule (number of steps, solvent identity, volume ratio), aging time and temperature, and drying route with its associated set-point profile. Objectives would be minimized thermal conductivity, maximized compressive modulus, minimized solvent intensity in kilograms of solvent per kilogram of dry aerogel, and minimized drying energy in kilowatt-hours per kilogram of dry aerogel, with shrinkage and yield entered as constraints so that low-burden but structurally failed samples cannot dominate the Pareto front. An initial design of experiments would train Gaussian-process surrogates for each objective; a multi-objective Bayesian or NSGA-II loop would then propose candidates on the estimated Pareto front, and only these would be synthesized and characterized, with solvent and energy recorded for each batch rather than estimated afterward. The output would be a Pareto set making the performance–burden exchange rate explicit, for example, the additional drying energy required per unit reduction in thermal conductivity. We note explicitly that, to our knowledge, no published study has yet implemented such a four-objective loop for polymer aerogels: existing multi-objective work optimizes performance and material dosage but does not carry solvent and drying burdens as objectives. Realizing this workflow therefore depends on the processing metadata mandated in Section 6.5, and we present it here as a concrete near-term target rather than as an established result.

### 6.3. Knowledge Mining and Generative Design: Design Space Without Laboratory Consumption

A complementary route to dissolving the sustainability paradox is to extract design knowledge from the literature already published rather than to generate new experimental data. Natural-language-processing tools have evolved rapidly in this space: from rule-based pipelines such as ChemDataExtractor [136], through domain-adapted transformer models including MatBERT [137], MatSciBERT [138], and MaterialsBERT [139], to large-language-model-based pipelines such as ChatExtract [140] and continually pretrained foundation models such as LLaMat [141]. Collectively, these tools now extract chemical entities, processing conditions, and property values from full-text articles with precision and recall well above earlier rule-based systems. In a directly aerogel-relevant demonstration, Takeshita et al. [24] mined 17,429 aerogel papers and 16,559 polymer aerogel samples to quantify density-dependent porosity, surface area, and thermal-conductivity trends, and—importantly—to flag systemic reporting bias in thermal-conductivity values. This finding underscores a key opportunity for cleaner production: NLP can recover not only performance metadata but also solvent volumes, drying routes, and yield indicators on which life-cycle assessment depends, transforming dispersed literature into a sustainability-relevant corpus without a single new synthesis.

Generative models extend this principle from extraction to creation, producing architectures that have not yet been synthesized. Generative adversarial networks, variational autoencoders, graph neural networks, and—most recently—diffusion models have generated 3D porous microstructures, zeolite topologies, and amorphous oxide networks with validity rates orders of magnitude higher than earlier methods [142,143,144]. To date, no published generative model has been trained de novo on polymer aerogel structures. Nevertheless, the methodological precedent in amorphous and porous systems—together with the natural fit of graph representations to crosslinked polymer networks—establishes a clear pathway toward generating biomass-derived or vitrimer-based architectures conditioned simultaneously on hierarchical-porosity targets and recyclability constraints. Such conditional generative pipelines would, in principle, allow polymer aerogels to be designed directly against cleaner-production objectives—for example, maximizing renewable-carbon fraction at fixed thermal-insulation performance—closing the gap between literature mining and experimental synthesis that Section 6.4 addresses next.

### 6.4. Self-Driving Laboratories: Closing the Loop on Sustainable Aerogel Discovery

Self-driving laboratories (SDLs) close the digital loop by integrating algorithmic decision-making with automated synthesis and characterization, and they constitute arguably the most direct experimental platform for resolving the sustainability paradox in materials discovery. Reported SDLs have autonomously synthesized 41 new inorganic compounds in 17 days [145], discovered photocatalysts six times more efficient than baseline in eight days [146], and identified high-cycle-life Li-ion charging protocols in 16 days versus over 500 days for exhaustive search [147]. Each of these numbers represents not only a discovery acceleration but also a cleaner-production gain: closed-loop search consumes far fewer reagents, less drying energy, and less analytical time per validated outcome.

Although no SDL has yet targeted polymer aerogel synthesis specifically, several features of the aerogel design space make it among the most promising candidates for SDL deployment. First, the parameter space—spanning precursor selection, sol–gel stoichiometry, catalyst type and concentration, aging time and temperature, solvent-exchange gradients, and drying route—is high-dimensional yet experimentally bounded by practical reagent and equipment limits. Second, characterization can be automated through inline rheometry during gelation, thermogravimetric analysis post-drying, and image-based porosity quantification, providing multiple property channels per autonomous cycle. Third, and most importantly for sustainable aerogel design, the optimization objective itself can be defined to include solvent recovery ratio, drying energy, and end-of-life recyclability rather than performance metrics alone, turning every closed-loop iteration into a step toward born-sustainable materials.

Realizing an aerogel SDL nevertheless faces three practical obstacles. Drying—particularly supercritical drying—currently has cycle times of hours to days that limit autonomous throughput; solvent-exchange protocols often require multi-stage handling that is difficult to automate without dedicated fluidic infrastructure; and standardized characterization of nanoscale porosity and cyclic durability has not yet been integrated into commercially available SDL platforms. Hybrid strategies—for example, autonomous synthesis combined with batched human-in-the-loop drying and characterization, or surrogate-model-mediated screening followed by selective experimental validation—provide credible near-term pathways toward aerogel-relevant SDLs. The data-infrastructure prerequisites for any of these strategies, however, are not yet in place, as the next subsection details.

### 6.5. Limitations and the Data Infrastructure Required for Sustainability-Aware AI

The sophistication of computational and AI tools now far exceeds the quality of the data on which they are trained. Aerogel datasets typically contain 30–200 entries, well below the thresholds of approximately 100 samples for classical ML and 500 samples for deep learning identified by Zhang and Ling [148]. The rigorously curated silica-aerogel database reported by Walker et al. [130] contained only 103 reliable entries despite extensive automated mining. Vishnyakov [149] also observed that most aerogel ML models are trained on a single laboratory’s local dataset, which limits generalization. The aerogel literature also shows documented “academic fever” in thermal-conductivity reporting and concentrated reporting biases in freeze-dried organic systems [24]. For sustainability-oriented research, an additional concern is that the processing metadata required for life-cycle assessment—solvent volumes, recovery ratios, drying energy, yields, and end-of-life pathways—are reported even less consistently than performance properties. Addressing this gap will require FAIR (Findable, Accessible, Interoperable, Reusable) data infrastructure on the scale already established for crystalline materials by the Materials Project [150] and AFLOW [151], together with rigorous uncertainty quantification so that AI predictions can be used with calibrated confidence. Building on prior community recommendations, we propose five reporting mandates tailored specifically to cleaner-production-aware aerogel research, summarized in Table 4.

Because several of the strategies discussed above rest on achievements in adjacent materials systems rather than on direct demonstrations in polymer aerogels, Table 5 classifies them explicitly into three levels of evidence: (i) established aerogel-specific evidence; (ii) methodologies partially demonstrated for aerogels but not yet integrated with sustainability objectives; and (iii) methodologies transferable from other material classes that remain future opportunities. Statements in this section should be read against this classification, and Level 3 entries should be understood as prospective rather than as established technologies for polymer aerogel design.

## 7. Applications of Sustainable Polymer Aerogels

The application landscape of polymer aerogels is governed by the coupling of pore architecture, interfacial chemistry, density, and network mechanics. In practice, the same structural features that suppress heat transfer can also improve sorption kinetics, facilitate electrolyte diffusion, expose catalytic sites, or emulate the transport-rich microenvironment of biological tissues. This cross-functionality explains why polymer aerogels are increasingly discussed not as niche insulators but as a broader platform for sustainable multifunctional materials [24]. Section 7.1, Section 7.2, Section 7.3, Section 7.4, Section 7.5 and Section 7.6 examine this platform across six application families—thermal insulation, environmental remediation, carbon capture, energy storage and catalysis, biomedical use, and emerging multifunctional domains—through the lens of service-based performance rather than capacity in isolation.

### 7.1. Thermal Insulation and Thermal Safety

Thermal management remains the most mature application domain for polymer aerogels. Their insulating performance derives from the suppression of gas-phase heat transport in nanoscale pores, the extension of tortuous heat paths through sparse solid networks, and, in advanced formulations, the attenuation of radiative transport. Compared with brittle conventional inorganic aerogels, polymeric systems such as polyimide, aramid, polyurethane, and hybrid fiber-reinforced aerogels offer a more practical balance of low thermal conductivity, flexibility, recoverability, and mechanical integrity under service conditions [91,152]. Recent 2025 advances illustrate the direction of travel: gradient all-nanostructured aramid aerogel fibers have been reported with radial thermal conductivity as low as 0.0228 W m^−1^ K^−1^ alongside tensile strengths up to 29.5 MPa and fracture strains of 39.2% [118]. At the application level, flexible thermal-insulation reviews now explicitly identify aerospace, buildings, and battery safety as leading deployment spaces [152]. Future sustainable aerogels for insulation should therefore not be optimized for low κ alone, but engineered for low κ under compression, high humidity tolerance, flame resistance, and manufacturable form factors such as fibers, films, felts, or modular monoliths.

### 7.2. Environmental Remediation and Separations

Polymer aerogels are particularly attractive for water and wastewater treatment because their accessible macropore–mesopore networks enable rapid transport while surface groups provide pollutant-specific affinity. In cellulose-, chitosan-, alginate-, and composite aerogels, pollutant removal can proceed through a combination of physical sorption, electrostatic attraction, coordination or ion exchange, interfacial wettability control, catalytic degradation, and photothermal enhancement [153]. The same material family is therefore relevant to oil–water separation, dye removal, heavy-metal capture, microplastic removal, and even solar-driven desalination [153]. For sustainable polymer aerogels, the decisive issue is not just uptake capacity but reusability under realistic matrices: salt, surfactants, natural organic matter, pH swings, and repeated regeneration often determine translational value more than single-cycle batch performance [153]. Next-generation remediation aerogels are therefore best understood as selective, regenerable interfacial reactors rather than passive sponges.

### 7.3. Carbon Capture and CO_2_ Adsorption

Carbon capture places stricter demands on aerogel design because performance depends simultaneously on pore structure, active-site chemistry, moisture response, and desorption energy. Carbon aerogels and amine-functionalized biopolymer aerogels are promising because their 3D open networks minimize diffusion limitations while micropores and basic sites increase affinity for CO_2_ at low partial pressure [91]. Recent work on functionalized cellulose aerogels for moisture-swing direct air capture (DAC) illustrates the broader trend that equilibrium capacity alone is no longer a sufficient descriptor: maleic acid–sodium hypophosphite-crosslinked quaternized cellulose aerogels reached a CO_2_ capacity of 2.37 mmol g^−1^ while maintaining structural stability over 30 dry–wet cycles, and composite formulations with cation exchange resins achieved 3.71 mmol g^−1^ with rapid kinetics [154]. Candidate sorbents must therefore be evaluated by working capacity, adsorption rate, regeneration conditions, long-term cyclic stability, and resistance to deactivation under humid air [91]. This is also the direction emerging from recent NIST-led characterization work, which emphasizes that DAC sorbents should be benchmarked under appropriate low-concentration conditions using harmonized test procedures and a broad characterization suite encompassing isotherms at sub-ambient CO_2_ partial pressures, humidity-resolved kinetics, and oxidative stability under cycling [155]. Sustainable polymer aerogels are therefore especially promising for DAC when they combine renewable feedstocks with application-relevant cyclic metrics rather than high static capacities measured only at 1 bar dry CO_2_.

### 7.4. Energy Storage and Catalytic Platforms

In electrochemical systems, polymer-derived carbon aerogels and conductive hybrid aerogels provide a continuous 3D electron-conduction backbone, hierarchical ion-transport pathways, and abundant accessible surface area. These features support use as binder-free electrodes, structured current collectors, catalytic scaffolds, and porous hosts for active phases in supercapacitors, batteries, and fuel-cell-related electrocatalysis [156]. The contemporary design logic is to exploit macropores as ion-buffering reservoirs, mesopores as transport bridges, and micropores as charge-storage or adsorption sites, while heteroatom doping and interfacial hybridization introduce additional faradaic activity or catalytic functionality [156]. Across recent reviews, carbon aerogels are repeatedly highlighted as multifunctional platforms spanning energy storage, catalysis, gas separation, and remediation [156], reinforcing the value of explicit structure–property mapping for this material class. From the catalysis perspective, polymer aerogels function either as active catalysts or as robust supports that maximize dispersion and accessibility of catalytic centers. Their advantages are most obvious in photocatalysis and electrocatalysis, where high surface area, low diffusion resistance, and self-supported 3D architectures mitigate nanoparticle agglomeration and improve reactant access [156]. For sustainable design, however, a more meaningful benchmark is not peak activity in ideal electrolytes or model contaminants but the retention of activity, resistance to leaching, recyclability, and fabrication from low-toxicity precursors or biomass-derived carbons [91,156]. This distinction aligns application performance with genuine sustainability rather than performance isolated from life-cycle context.

### 7.5. Biomedical Applications

Biopolymer aerogels are especially compelling for biomedical use because their low density, high porosity, wettable surfaces, and tunable degradation allow them to emulate several functional aspects of the extracellular matrix. Current literature supports their use as scaffolds for tissue regeneration, wound dressings, drug-delivery carriers, and biosensor interfaces, particularly when based on cellulose, chitosan, alginate, gelatin, or silk fibroin [157]. Their principal advantages are not merely biocompatibility, but the combination of interconnected pores for nutrient transport, accessible loading volume for therapeutics, and mechanically compliant architectures that can be chemically or biologically functionalized [157]. At the same time, translational maturity remains limited. Recent reviews indicate that, despite strong preclinical activity, aerogel formulations for wound care and regenerative medicine have not yet moved into registered human trials [157], underscoring the gap between elegant laboratory materials and clinically qualified products. Biomedical aerogels therefore remain a high-potential but still preclinical domain in which sterilization, endotoxin control, long-term biosafety, wet-state mechanics, and batch reproducibility are as important as scaffold porosity or drug-loading efficiency.

### 7.6. Emerging Applications

Beyond the established domains above, polymer aerogels are increasingly entering EMI shielding, acoustic attenuation, wearable sensing, and multifunctional protective textiles. Here, ultralow density alone is not sufficient; useful materials must integrate conductivity, impedance matching, mechanical resilience, and, ideally, absorption-dominant shielding to minimize secondary reflection [158]. Recent studies on anisotropic composite aerogels and conductive cellulose-based aerogels show that high shielding effectiveness can be combined with flexibility and wearability, while low-density polyimide aerogels with tailored solid content, a parameter that governs microstructure and mechanical response [159], have also shown meaningful sound-absorption behavior [158]. These trends mark a strategic shift toward multifunctionality per unit mass: the most valuable future aerogels may be those that simultaneously provide thermal insulation, acoustic damping, EMI attenuation, and sensing with minimal added material burden.

Across Section 7.1, Section 7.2, Section 7.3, Section 7.4, Section 7.5 and Section 7.6, the application map indicates that sustainable polymer aerogels are best categorized not by chemistry alone, but by performance under application-relevant boundary conditions: thermal insulation under load and humidity, contaminant removal in complex fluids, CO_2_ capture at low partial pressure with cyclic regeneration, electrochemical operation at practical current or mass loading, and biomedical function after sterilization and storage. Table 6 summarizes this service-based view across the six domains, contrasting laboratory metrics with the application-relevant performance, durability, and metadata needed for credible deployment claims. This perspective connects directly to the structure–property–performance maps developed in Section 5 and sets the stage for Section 8, where the same service-based logic is extended into a quantitative life-cycle assessment and circular-economy framework: an aerogel that excels at “performance under realistic conditions” must also be evaluated for the resource and environmental costs of delivering that performance over its useful life.

## 8. Sustainability, Life Cycle Assessment, and Circular Economy

The sustainability of polymer aerogels cannot be inferred from feedstock origin alone. A bio-based precursor does not automatically produce a low-impact material, and a high-performance thermoset aerogel is not automatically unsustainable if long service life, high use-phase savings, or credible chemical recycling can offset its production burden. The correct analytical framework is therefore life-cycle-based and system-dependent, spanning precursor sourcing, solvent and energy demand, drying route, product durability, occupational safety, and end-of-life recovery [91,94]. Section 8.1, Section 8.2, Section 8.3, Section 8.4, Section 8.5, Section 8.6 and Section 8.7 develop this framework across green synthesis, comparative life-cycle assessment, end-of-life pathways, circularity, scalability, open challenges, and the reporting standards required to make all preceding claims auditable.

### 8.1. Green Synthesis and Renewable Precursors

A sustainable synthesis pathway begins with benign or lower-impact feedstocks and then depends heavily on process intensification. Recent sustainability reviews identify the same recurring priorities: water-based or low-toxicity sol–gel routes, replacement of problematic solvents where possible, valorization of biorefinery outputs and waste-derived polymers, solvent minimization during exchange, and drying strategies that preserve porosity without imposing a disproportionate energy penalty [91,94]. Waste and byproduct streams are especially attractive because they connect aerogel fabrication to resource valorization rather than virgin extraction, and recent literature shows viable routes from food waste, textile waste, paper waste, recycled PET, and other secondary resources [91]. Demonstrated examples span end-of-life tire rubber [160], recycled textile fibers [161], and wool waste fibers for oil-spill remediation [162]. At the same time, drying remains a pivotal processing step bottleneck. Freeze-drying is widely used for biopolymer aerogels but is repeatedly identified as energy-intensive; supercritical drying preserves structure well and may be improved through solvent/CO_2_ recovery and process integration; ambient-pressure strategies offer scale-up advantages but require careful control of shrinkage and network chemistry [94]. Recent sustainability reviews further note that continuous-mode solvent exchange can reduce solvent consumption to roughly one-third of batch processing, and that process integration can combine steps such as exchange, drying, sterilization, or heat recovery to reduce total burden [91].

### 8.2. Comparative Life-Cycle Assessment

The strongest conclusion from recent aerogel LCA literature is methodological heterogeneity. Existing studies differ in functional units, system boundaries, electricity mixes, database choices, allocation procedures, and scale assumptions, making direct comparison hazardous unless service equivalence is respected [94]. This is fully consistent with ISO 14044, which requires explicit definition of goal and scope, functional unit, system boundary, data quality requirements, assumptions, impact method, and interpretation procedure [163]. For aerogels, this means that comparisons should move away from “impact per kilogram of material” whenever the function is insulation, adsorption, or controlled release. A fair comparison is usually service-based: thermal resistance delivered over a defined lifetime, pollutant removed over a specified number of regeneration cycles, or CO_2_ captured per lifetime sorbent mass under stated operating conditions [91,94]. The literature also makes clear that solvent exchange and drying frequently dominate environmental burden, while freeze-drying, supercritical drying, and ambient drying each shift impacts differently between energy use, solvent use, and structural yield [94]. LCA should therefore be used not as a decorative sustainability claim, but as a design tool that identifies hotspots early and aligns material performance with delivered service.

A qualitative comparison of the primary assessments currently available illustrates why this heterogeneity matters in practice. Turhan Kara et al. [94] reviewed aerogel-specific life-cycle studies and identified solvent exchange and drying as the dominant production burdens, while also showing that the underlying studies adopt incompatible functional units and system boundaries. Lu et al. [59] categorized 174 bio-based insulation materials and reported that the embodied carbon of most is comparable to that of glass wool, indicating that renewable origin alone does not confer a climate advantage at the material level. Cascione et al. [73] extended the methodology further, combining scenario-based and dynamic assessment of eleven commercially available bio-based insulations over a 60-year reference period on a cradle-to-grave basis, and demonstrated that conclusions shift appreciably with assumptions on manufacturing, transport and end-of-life treatment. Read together, these studies differ in scope (aerogel-specific versus insulation-wide), in boundary (cradle-to-gate versus cradle-to-grave), and in their treatment of biogenic carbon and service life. None reports an aerogel system on a basis directly comparable with the others, which is precisely why a quantitative cross-study table cannot yet be assembled without misrepresenting the individual sources.

Because drying dominates both cost and environmental burden, the choice of drying route should follow an explicit decision sequence rather than laboratory convention. We suggest four sequential questions. (i) Does the application require a monolith with preserved mesoporosity and thermal conductivity below approximately 25 mW m^−1^ K^−1^? If so, supercritical drying remains the reference route, and the decision then turns on whether CO_2_ recovery above 90% can be demonstrated at the intended scale. (ii) If moderate shrinkage and a somewhat higher thermal conductivity are acceptable, can the network be stiffened or surface-modified sufficiently for ambient-pressure drying? Ambient-pressure drying is normally the lowest-energy option and should be the default whenever the performance target permits it. (iii) Is an anisotropic or aligned macroporous architecture functionally required, as in directional transport, adsorption, or acoustic applications? Freeze-drying is then justified by structure rather than by convenience, and its high specific energy demand must be reported explicitly. (iv) Finally, does the selected route remain preferable when normalized to the service-based functional unit and to the durability of the resulting material rather than to a kilogram of dry aerogel? Applying this sequence, and reporting the answer to each question, would make drying selection auditable and would prevent the common situation in which the most energy-intensive route is adopted without functional justification.

### 8.3. End-of-Life Pathways, Dynamic Networks, and Vitrimer Logic

End-of-life remains the most decisive distinction between different aerogel families. Biopolymer aerogels may offer easier biodegradation or compostability in principle, but realistic disposal conditions, additives, crosslinkers, and contamination history must still be considered before such claims are made [91]. Conventional petroleum-derived thermoset aerogels are more problematic because they combine long-lived covalent networks with limited reprocessability. This is precisely where dynamic covalent chemistry has changed the field. Dynamic covalent polymer aerogels (DCPAs) based on imine chemistry have already demonstrated weldability, repairability, degradability, and closed-loop recyclability under mild or catalyst-free processing [164], while subsequent work has introduced the aerogel-to-sol-to-aerogel concept as a practical route for recycling, repairing, and reprogramming high-performance organic aerogels [91]. More recently, polyhexahydrotriazine-derived aerogels were shown to preserve nanoscale porosity and insulation performance across recycling cycles while also enabling property retuning in subsequent generations [119]. These advances support a stronger position than feedstock-based circularity arguments alone: circularity should be designed into sustainable polymer aerogels at the molecular-network level, not postponed as a downstream waste-management problem.

### 8.4. Circular Economy Framework

A rigorous circular framework for polymer aerogels can be articulated as a six-step loop: waste or renewable precursor sourcing, green sol–gel manufacture, long-lived service in an insulation or separation role, controlled end-of-life activation, depolymerization or network disassembly, and reprogramming or upcycling into a new aerogel generation. Recent sustainability reviews emphasize that current circular practice in aerogel manufacturing still focuses more on recycling solvents and supercritical CO_2_ than on recycling the aerogel bodies themselves [91]. However, the literature is clearly expanding toward both feedstock circularity and product circularity, with examples spanning recycled PET aerogels, waste-derived cellulose aerogels, reusable remediation sorbents, and dynamic polymer networks that can be chemically disassembled and rebuilt [119,164]. The most credible circular aerogel platform will therefore combine renewable or waste-derived inputs with repairable or chemically recyclable outputs, addressing both the front end and the back end of the life-cycle.

### 8.5. Scalability and Techno-Economic Analysis

Scale-up remains constrained less by materials discovery than by manufacturing reality. The principal cost drivers are still precursor purification, solvent handling, long aging times, drying throughput, and the mismatch between laboratory batch processing and industrial continuous production [91,94]. Sustainability reviews highlight an additional structural problem: although bio-based aerogels are widely studied, no large-scale production facilities have yet been established that can deliver true industrial volumes for most biopolymer aerogel classes [91]. This means techno-economic analysis (TEA) must be integrated much earlier in research programs. Promising directions include continuous flow processing, modular solvent-exchange trains, ambient-pressure or hybrid drying where property retention permits, use of technical solvent mixtures, integration of waste-heat recovery, and direct conversion of low-value waste streams into application-ready formats [91]. Recycled-polymer aerogels offer another attractive path because they can turn waste-management liabilities into products with useful insulation and acoustic performance, thereby improving both material circularity and economic justification. Future aerogel scale-up studies should therefore report not only laboratory performance, but also mass balance, solvent recovery ratio, drying time, throughput, yield loss, shaping route, and projected cost sensitivity to electricity price and solvent recycle efficiency.

### 8.6. Open Challenges and Future Directions

Three challenges now define the field. First, sustainability claims remain fragile without harmonized testing and service-based LCA. Second, circularity is far more advanced for dynamic covalent organic aerogels than for many high-performance hybrid or carbonized systems [91,164]. Third, safety data remain incomplete, particularly for dust generation, inhalation exposure, chronic biocompatibility, and leachables from functional additives [91]. The corresponding research priorities, and the scientific and industrial barriers that accompany them, are set out in Section 9.4 rather than duplicated here. Special emphasis should be placed on durability-aware LCA, because highly efficient but short-lived aerogels can underperform less spectacular materials at the system level [94]. For biomedical and direct-air-capture applications, standardization is especially urgent, because device-level translation depends on reliable characterization under realistic humidity, concentration, sterilization, and storage conditions rather than idealized single-point metrics [155].

### 8.7. Recommended Reporting Standards and Metadata for Reproducibility

For the topics covered in these sections, a minimum reporting package should align with IUPAC recommendations for physisorption-based surface-area and pore-size analysis [165], emerging test-method standardization priorities from NIST for DAC sorbents [155], and the methodological requirements of ISO 14044 for life-cycle studies [163]. In practice, this means reporting not only “what the aerogel did,” but also precisely “how it was made, how it was tested, under what boundary conditions, and how long the performance was retained.” Without that metadata, cross-study comparisons and AI-ready data extraction remain unreliable [24]. Adoption of these reporting practices would make performance claims reproducible, sustainability claims auditable, and the resulting dataset suitable for cross-study comparison and data-driven design [24,91].

Section 8.1, Section 8.2, Section 8.3, Section 8.4, Section 8.5, Section 8.6 and Section 8.7 move from feedstock origin to life-cycle-based assessment, end-of-life and circularity, and the manufacturing and reporting conditions needed to support these claims. The next step is to translate this analysis into a design and deployment roadmap. Section 9 therefore brings together the findings of Section 2, Section 3, Section 4, Section 5, Section 6, Section 7 and Section 8 as short-, medium-, and long-term priorities for moving polymer aerogels toward deployable, sustainable systems in which performance, manufacturability, life-cycle outcomes, and circular endpoints are considered together.

## 9. Outlook: From High-Performance Porous Materials to Deployable Sustainable Systems

The field of polymer aerogels is currently at a critical inflection point, transitioning from lab-scale demonstrations of extreme properties to the realization of functional, sustainable, and scalable materials. As established in Section 8, the environmental viability of these highly porous networks is heavily dictated by solvent intensity, processing energy, and end-of-life (EoL) scenarios. This section has a narrower role than the preceding sections: whereas Section 2, Section 3, Section 4, Section 5 and Section 6 establish the current state of knowledge and Section 7 and Section 8 evaluate performance and life-cycle outcomes, Section 9 sets out our recommendations and the research agenda we consider necessary. To avoid duplication, the standardization requirements are not restated here but are summarized later in Table 7; the discussion here focuses on the remaining priorities, organized by time horizon.

To support reproducible evaluation and sustainability assessment, Table 7 summarizes the minimum reporting standards and metadata requirements recommended for sustainable polymer aerogel research.

### 9.1. Short-Term Priorities: Standardization and LCA-Ready Data

In the short term, the most urgent priority is community adoption of the reporting requirements already specified in Table 4 (data infrastructure) and Table 7 (application-level metadata). The main challenge is no longer defining what should be reported, but achieving wider uptake: adoption should be driven through journal and funder requirements rather than left to individual practice.

### 9.2. Medium-Term Priorities: Process Intensification and Pilot-Relevant Optimization

In the medium term, the field must shift from laboratory recipes to pilot-relevant process windows. Many aerogel studies still depend on batch gelation, multiple solvent exchanges, long aging, and freeze- or supercritical drying. These routes are useful for discovery but difficult to justify as cleaner production unless they are redesigned for efficiency, recovery, and scale.

Promising directions include ambient-pressure drying with network strengthening, water-based sol–gel systems, continuous or semi-continuous solvent exchange, reactive or low-solvent processing, waste-heat integration, renewable electricity for drying, and modular manufacturing. AI and digital twins can support this transition by predicting shrinkage, cracking, drying time, and quality variability before scale-up. Importantly, optimization should include yield, cycle time, energy use, solvent recovery, and defect rate, not only final material properties.

### 9.3. Long-Term Priorities: Born-Sustainable Aerogels

The long-term vision is the development of born-sustainable aerogels: materials designed from the outset to satisfy performance, processing, life-cycle, and circularity criteria. In such systems, renewable or recycled feedstocks, safer solvents, dynamic or degradable networks, durable service behavior, and end-of-life recovery are integrated at the molecular-design stage rather than added after performance optimization.

This will require combining the digital tools assessed in Section 6—in particular the Level 3 methods of Table 5, whose transfer to aerogels remains unproven—with in-line metrology and embedded life-cycle and techno-economic modules, so that energy and solvent inputs are recorded as experimental outputs rather than reconstructed afterward.

### 9.4. Remaining Scientific and Industrial Barriers

Several barriers must still be addressed. Scientifically, predictive control over non-equilibrium gelation, phase separation, drying shrinkage, and multiscale structure formation remains incomplete. Dynamically recyclable networks must also demonstrate long-term dimensional stability, creep resistance, and property retention under realistic service conditions. Biopolymer aerogels must overcome moisture sensitivity, feedstock variability, and processing reproducibility.

Industrially, barriers include cost, throughput, quality control, safety, certification, and market-specific requirements. Building insulation requires fire, durability, moisture, and installation standards. Sorbents require regeneration performance and contaminant handling. Biomedical aerogels require sterilization, biocompatibility, and regulatory validation. Conductive aerogels require device-level integration and end-of-life management of nanofillers. These constraints should be treated as design inputs rather than late-stage obstacles.

### 9.5. Concluding Perspective for Cleaner Production

The key question for the field is not whether an aerogel achieves low density, high porosity or strong mechanics, but whether it delivers a useful service at lower life-cycle burden and with a more credible circular pathway than the available alternatives.

Table 8 assigns units to the sustainability descriptors mandated in Table 4 (row iii); the two tables are intended to be read together, with Table 4 specifying what must be reported and Table 8 specifying how it should be quantified.

**Table 8:** Recommended sustainability metrics for polymer aerogel studies.

Metric	Recommended Unit	Design Relevance
Cradle-to-gate GWP	kg CO_2_-eq per kg and per functional unit	Quantifies production-stage climate burden
Cumulative energy demand	MJ per kg and per functional unit	Identifies energy hotspots, especially drying
Solvent intensity	kg solvent per kg dry aerogel	Captures solvent exchange burden
Solvent recovery ratio	% recovered	Distinguishes laboratory feasibility from scalable production
Drying energy	kWh per kg dry aerogel	Links processing route to environmental burden
Product yield and shrinkage	% yield; % volume shrinkage	Reflects material loss and structural fidelity
Renewable/recycled content	wt%; carbon fraction	Separates feedstock circularity from product circularity
Durability retention	% property retained after cycles or aging	Prevents overclaiming short-lived performance
Regeneration/recycling burden	MJ, solvent mass, time per cycle	Evaluates whether circularity creates new impacts
Service-based metric	Application-specific	Connects material performance to delivered function

A roadmap summarizing the short-, medium-, and long-term priorities for transitioning polymer aerogels toward cleaner production and deployable sustainable systems is provided in Table 9.

**Table 9:** Roadmap for cleaner-production-oriented polymer aerogel development.

Time Horizon	Priority	Key Actions	Expected Outcome
Short term (1–3 years)	LCA-ready standardization	Report complete processing metadata, uncertainty, durability, and functional units	Comparable datasets for AI, LCA, and review-level benchmarking
Medium term (3–7 years)	Pilot-relevant process intensification	Reduce solvent exchange, shorten drying, improve yield, and integrate process monitoring	Lower embodied energy and stronger scale-up credibility
Medium term (3–7 years)	AI/LCA/TEA integration	Use multi-objective optimization with performance and sustainability constraints	Pareto-optimal aerogel designs rather than single-property maxima
Long term (>7 years)	Born-sustainable aerogels	Combine renewable/recycled feedstocks, dynamic networks, digital twins, and circular end-of-life	Deployable aerogel systems for cleaner production and circular material flows

## 10. Conclusions

Polymer aerogels are fundamentally nonequilibrium materials whose macroscopic performance emerges from the coupled kinetics of gelation, phase separation, kinetic arrest, and drying across multiple length and time scales. Biopolymer-derived and thermoset aerogels occupy complementary regions of the property space, with hybrid and dynamic covalent systems now beginning to bridge the long-standing trade-off between high performance and circularity. In particular, the aerogel-to-sol-to-aerogel paradigm enabled by vitrimer and dynamic covalent chemistry demonstrates that circularity can be embedded directly into network design rather than imposed as an end-of-life afterthought, while Ashby-type property maps reveal that the most promising sustainability-rich and high-performance design regions remain underexplored. At the same time, four persistent research gaps continue to limit translation toward cleaner-production technologies: sustainability is still often treated qualitatively and discussed separately from the material-class analysis; processing histories, shrinkage, density, and durability protocols are inconsistently reported, weakening both life-cycle assessment and machine-learning model development; cleaner-processing strategies remain less developed than chemical innovations, with solvent volumes, drying energy, recovery ratios, and yields rarely quantified; and circularity claims frequently conflate bio-based content, biodegradability, recyclability, and reusability, which in fact represent distinct end-of-life pathways.

To make these four categories measurable and comparable, we propose a distinct parameter for each. Bio-based content should be reported as renewable carbon fraction (wt% or ^14^C-based biogenic carbon content), not as a qualitative claim about feedstock origin. Biodegradability should be reported as mineralization degree under a named standard test, environment, and duration, since degradation in industrial composting does not imply degradation in soil or seawater. Recyclability should be reported as recovery yield together with property retention after a stated number of closed-loop cycles, specifying whether recycling is mechanical, dissolution-based or chemical. Reusability should be reported as the number of regeneration cycles achieved in the actual service matrix, together with the energy and solvent cost of each regeneration step. As a working threshold for claims of genuine circularity, we suggest that a material retain at least 80% of the property that defines its function—thermal conductivity, adsorption capacity or compressive modulus, as appropriate—over at least five recycling or regeneration cycles, with the regeneration burden itself included in the assessment. This threshold is proposed as a reporting convention to enable comparison rather than as a physically derived limit, and the community should refine it as cycle-resolved datasets accumulate; what matters is that circularity claims specify which of the four pathways is meant, over how many cycles, and at what environmental cost.

To address these gaps, future research should pursue several integrated directions. First, environmental descriptors—including solvent intensity, drying energy, renewable or recycled content, hazard profile, regeneration cycles, and end-of-life pathway—must be embedded as explicit design variables from the molecular stage rather than appended post hoc as sustainability justifications. Second, FAIR-compliant aerogel databases with standardized processing metadata, uncertainty quantification, and service-based functional units are required to enable both rigorous life-cycle and techno-economic assessment and reliable machine-learning model development. Third, multiscale physics-based modeling should be tightly coupled with experimental validation, Bayesian optimization, generative inverse design, and autonomous self-driving laboratories so that synthesis conditions can be steered toward target structures and properties with explicit environmental objectives rather than purely performance-driven ones. Finally, future work should move toward a “born-sustainable” design philosophy in which renewable or recycled feedstocks, dynamic covalent networks, validated end-of-life pathways, and deployable manufacturing routes are co-designed at the molecular stage. An integrated, service-normalized, and life-cycle-aware approach is therefore needed to move sustainable polymer aerogels from laboratory-scale demonstrations toward deployable cleaner-production technologies with verifiable environmental benefits across their full life cycle.

## Figures and Tables

**Table 2:** Recommended descriptors for a sustainable polymer aerogel database.

Data Category	Key Descriptors	Design Relevance
Composition	Polymer/biopolymer type, renewable fraction, recycled fraction, crosslinker, filler, functional groups	Links chemistry to sustainability and functionality
Processing	Gelation route, solvent, aging, solvent exchange, drying method, shrinkage	Captures path-dependent structural formation
Structure	Density, porosity, BET surface area, pore size distribution, ligament size, anisotropy, tortuosity	Defines the architectural basis of performance
Mechanical properties	Compressive modulus, compressive strength, strain recovery, fatigue resistance, toughness	Determines load-bearing, resilience, and durability
Thermal properties	Thermal conductivity, temperature stability, radiative contribution, humidity sensitivity	Determines insulation and thermal protection suitability
Transport and functional properties	Adsorption capacity, diffusion rate, permeability, electrical conductivity, ion transport	Determines performance in remediation, catalysis, energy, and sensing
Sustainability metrics	Bio-based content, recyclability, degradability, solvent toxicity, drying energy, life-cycle indicators	Enables comparison beyond performance alone

**Table 3:** Qualitative cross-class comparison of sustainable polymer aerogel families.

Aerogel Class	Typical Structural Feature	Dominant Strengths	Main Limitations	Most Suitable Applications
Cellulose/chitin/chitosan aerogels	Nanofibrillar or polysaccharide networks	Renewable, functional groups, low density, biocompatibility	Moisture sensitivity, limited thermal stability	Adsorption, filtration, biomedical scaffolds, packaging
Lignin/protein/starch/alginate aerogels	Aromatic or physically/ionically crosslinked networks	Bio-based content, chemical functionality, low cost potential	Batch variability, weaker mechanical stability	Water treatment, carbon precursors, agriculture, controlled release
Conventional thermoset aerogels	Covalently crosslinked networks	Thermal stability, stiffness, chemical resistance	Poor recyclability, higher processing intensity	Aerospace insulation, structural insulation, flame resistance
Carbonized thermoset aerogels	Conductive carbon networks	Electrical conductivity, high surface area, electrochemical activity	Energy-intensive carbonization, brittleness	Supercapacitors, batteries, EMI shielding, catalysis
Vitrimer/recyclable thermoset aerogels	Dynamic covalent networks	Reprocessability, repairability, circularity potential	Early-stage field, limited datasets	Recyclable insulation, sensors, circular composites
Hybrid aerogels	Multiscale reinforced networks	Balanced mechanics, tunable functionality, multifunctionality	Complex synthesis, difficult standardization	High-performance insulation, wearable electronics, tissue engineering, multifunctional structures

**Table 4:** Proposed reporting mandates for sustainability-aware aerogel data infrastructure.

Mandate	Required Content	Cleaner-Production Rationale
(i) Core performance metrics	Density, porosity, BET surface area, full pore-size distribution, thermal conductivity, compressive modulus	Establishes a reproducible performance baseline for cross-study comparison and ML training
(ii) Full synthesis metadata	Precursor concentrations, solvent ratios, gelation kinetics, aging conditions, drying protocols	Captures the path-dependent processing history that determines both structure and embodied environmental burden
(iii) Sustainability metadata	Solvent volume, solvent recovery ratio, drying energy, renewable or recycled content, regeneration cycles, end-of-life pathway	Provides the explicit descriptors required for life-cycle assessment and sustainability-constrained inverse design
(iv) Raw machine-readable data	Characterization data deposited with persistent DOIs (e.g., Zenodo, Materials Cloud, Figshare)	Enables re-analysis, model retraining, and uncertainty quantification across laboratories
(v) Standardized nomenclature and uncertainty	SI units, IUPAC nomenclature, explicit uncertainty estimates (e.g., standard deviation, sample size, instrument resolution)	Enables calibrated AI prediction and avoids spurious benchmarking from incomparable reporting conventions

**Table 5:** Evidence level and maturity of computational and AI-guided methods for polymer aerogel design.

AI/Computational Method	Demonstrated Status for Polymer Aerogels	Typical Dataset Size	Properties or Outputs Addressed	Evidence Level
Multiscale physics-based modeling (molecular dynamics, mesoscale reconstruction, network/continuum upscaling)	Applied directly to aerogel microstructures and benchmarked against finite-element and Lattice Boltzmann references	Not data-limited (physics-based)	Mechanical, thermal and transport behavior; percolation	Level 1—established aerogel-specific evidence
Supervised forward machine learning (neural networks, tree ensembles, Gaussian processes, convolutional networks)	Demonstrated for silica, polyimide, MXene/cellulose and hybrid aerogel systems	Typically 30–200 entries; 103 curated entries in the most rigorous silica database	BET surface area, density, compressive modulus, effective thermal conductivity	Level 1—established aerogel-specific evidence
Natural-language-processing literature mining	Applied at scale to the aerogel corpus and used to expose systemic reporting bias	17,429 papers; 16,559 polymer aerogel samples	Density-dependent porosity, surface area and thermal-conductivity trends	Level 1—established aerogel-specific evidence
Multi-objective optimization (Bayesian optimization, NSGA-II, reinforcement learning)	Demonstrated for aerogel-containing composite panels; objectives so far exclude solvent and drying burdens	Case-specific	Thermal conductivity, compressive strength, density, aerogel dosage	Level 2—partially demonstrated; environmental objectives not yet integrated
Generative models (generative adversarial networks, variational autoencoders, graph neural networks, diffusion models)	No model trained de novo on polymer aerogel structures; precedent established in porous, zeolitic, and amorphous systems	Not established for aerogels	Proposed: hierarchical-porosity architectures conditioned on recyclability constraints	Level 3—transferable methodology/future opportunity
Self-driving laboratories	No self-driving laboratory has targeted polymer aerogel synthesis; precedent in inorganic synthesis, photocatalysis and battery protocols	Not established for aerogels	Proposed: closed-loop synthesis with solvent recovery and drying energy as explicit objectives	Level 3—transferable methodology/future opportunity
Digital twins for manufacturing	No peer-reviewed digital twin reported for aerogel production; component technologies individually mature	Not established for aerogels	Proposed: real-time control of drying and solvent gradients; batch-failure reduction	Level 3—transferable methodology/future opportunity

**Table 6:** Service-based performance framework for sustainable polymer aerogels across six application domains.

Application Domain	Laboratory Metric Typically Reported	Service-Relevant Metric	Core Sustainability Descriptor
Thermal insulation and thermal safety	Thermal conductivity κ under dry, unloaded conditions; density; compressive modulus on first cycle	Effective κ under compression, humidity, and thermal cycling; flame and thermal-shock response in the relevant form factor (fiber, film, felt, monolith)	m^2^·K·W^−1^ delivered over service lifetime; recyclability of monolith or felt; renewable or recycled feedstock fraction
Environmental remediation and separations	Single-cycle uptake capacity (mg g^−1^); equilibrium selectivity in clean water at fixed pH	Capacity after ≥10 regeneration cycles in real or simulated matrices (salt, NOM, surfactants, pH swings); leaching check; flow-cell stability	Total solvent intensity of synthesis; pollutant mass balance over service lifetime; documented fate of spent sorbent
Carbon capture and CO_2_ adsorption	Equilibrium CO_2_ capacity at 1 bar dry; BET surface area; amine loading	Working capacity at 400 ppm with humidity; adsorption rate; oxidative and thermal stability under multi-cycle operation	Regeneration energy per kg CO_2_; net CO_2_ captured per lifetime sorbent mass; renewable carbon fraction; sorbent end-of-life pathway
Energy storage and catalytic platforms	Specific capacitance, idealized TOF, peak Faradaic efficiency; conductivity in thin sample at low current	Areal and gravimetric performance at practical mass loading; long-cycle retention; rate capability in realistic electrolyte; leaching resistance	Solvent and energy intensity of fabrication; carbonization yield; recyclability of electrode and active phase; biomass-derived precursor fraction
Biomedical applications	In vitro cytocompatibility; drug-loading capacity and release profile; antimicrobial or hemostatic efficacy in model assays	Wet-state mechanics; sterilization stability; endotoxin control; in vivo tolerance where applicable; batch reproducibility	Renewable feedstock fraction; degradation-product safety; residual-solvent profile; explicit translational gap relative to clinical-grade qualification
Emerging applications (EMI, acoustic, sensing)	Total shielding effectiveness (SE_t_, dB); sound absorption coefficient; pressure or strain sensitivity	Multifunction per unit mass; absorption-dominant SE/SE_r_ ratio; flex and cycling stability; performance under humidity and temperature	Material burden per delivered function; integration and disassembly at end-of-life; recyclability of conductive components

**Table 7:** Minimum reporting standards and metadata for sustainability-aware polymer aerogel research.

Application or Claim Area	Minimum Performance Metrics	Essential Metadata to Report	Minimum Durability or Sustainability Evidence
Thermal insulation and fire safety	Thermal conductivity at defined temperature; density; compressive modulus or recovery; flame or thermal-shock response when relevant	Precursor identity and source; solid content; drying route; sample thickness; humidity; anisotropy; test standard or instrument; compression state during testing	Thermal cycling; compression cycling; aging under humidity; service-based functional unit such as m^2^·K/W delivered over lifetime
Water remediation and separations	Uptake capacity, selectivity, flux, removal efficiency, and regeneration performance	Pollutant identity; concentration; pH; ionic strength; water matrix; contact time; flow configuration; surface modification chemistry; leaching check	At least several regeneration cycles; performance in real or simulated complex water; mass balance for recovered pollutant and spent sorbent
CO_2_ capture and DAC	Equilibrium and working capacity; adsorption rate; breakthrough behavior; regeneration energy or temperature; cyclic stability	CO_2_ partial pressure; humidity; gas composition; bed geometry; sorbent loading; amine content; particle or monolith dimensions; test protocol	Multi-cycle retention under humid air; oxidative and thermal stability; lifetime-normalized CO_2_ captured per unit sorbent mass
Energy storage and catalysis	Areal and gravimetric performance; conductivity; rate capability; overpotential or Faradaic efficiency; catalyst utilization	Electrode thickness; mass loading; electrolyte; current density; binder content; carbonization conditions; pore hierarchy; active-phase distribution	Long-cycle stability; post-test structural analysis; catalyst leaching or degradation; energy or solvent intensity of fabrication
Biomedical applications	Cytocompatibility; wet-state mechanics; degradation profile; drug-loading/release behavior; antimicrobial or hemostatic efficacy where relevant	Sterilization route; endotoxin control; cell line or animal model; culture medium; scaffold pore architecture; residual solvent analysis; batch variability	Shelf stability; sterilization stability; in vivo tolerance where applicable; explicit regulatory or translational limitations
LCA and circularity claims	Functional unit; hotspot contribution; sensitivity and uncertainty analysis; end-of-life scenario comparison	Goal and scope; system boundary; geography; electricity mix; allocation method; solvent recovery ratio; yield; foreground data quality	Comparative scenario analysis; critical review if public comparisons are made; explicit discussion of reuse, repair, recyclability, or biodegradation conditions

## Data Availability

No new data were created or analyzed in this study. Data sharing is not applicable to this article.

## References

[1] Rouwhorst J., Ness C., Stoyanov S., Zaccone A., Schall P. (2020). Nonequilibrium continuous phase transition in colloidal gelation with short-range attraction. Nat. Commun..

[2] Rouwhorst J., Schall P., Ness C., Blijdenstein T., Zaccone A. (2020). Nonequilibrium master kinetic equation modeling of colloidal gelation. Phys. Rev. E.

[3] Tsurusawa H., Arai S., Tanaka H. (2020). A unique route of colloidal phase separation yields stress-free gels. Sci. Adv..

[4] Wang Z., Qiu W., Zhang Q. (2024). Constructing phase separation in polymer gels: Strategies, functions and applications. Prog. Polym. Sci..

[5] Ishikawa S., Iwanaga Y., Uneyama T., Li X., Hojo H., Fujinaga I., Katashima T., Saito T., Okada Y., Chung U.-I. (2023). Percolation-induced gel–gel phase separation in a dilute polymer network. Nat. Mater..

[6] Zhang G., Steck J., Kim J., Ahn C.H., Suo Z. (2023). Hydrogels of arrested phase separation simultaneously achieve high strength and low hysteresis. Sci. Adv..

[7] Yam B.J.Y., Le D.K., Do N.H.N., Nguyen P.T.T., Thai Q.B., Phan-Thien N., Duong H.M. (2020). Recycling of magnesium waste into magnesium hydroxide aerogels. J. Environ. Chem. Eng..

[8] Le D.K., Leung R.I., Alan S., Zhang X., Tay X.J., Thai Q.B., Phan-Thien N., Duong H.M. (2019). Applications of functionalized polyethylene terephthalate aerogels from plastic bottle waste. Waste Manag..

[9] Thai Q.B., Siang T.E., Le D.K., Shah W.A., Phan-Thien N., Duong H.M. (2019). Advanced fabrication and multi-properties of rubber aerogels from car tire waste. Colloids Surf. A Physicochem. Eng. Asp..

[10] Duong H.M., Ling N.R.B., Thai Q.B., Le D.K., Nguyen P.T.T., Goh X.Y., Phan-Thien N. (2021). A novel aerogel from thermal power plant waste for thermal and acoustic insulation applications. Waste Manag..

[11] Do N.H., Le T.M., Tran H.Q., Pham N.Q., Le K.A., Nguyen P.T., Duong H.M., Le T.A., Le P.K. (2021). Green recycling of fly ash into heat and sound insulation composite aerogels reinforced by recycled polyethylene terephthalate fibers. J. Clean. Prod..

[12] Rege A., Aegerter M.A., Leventis N., Koebel M.M., Steiner S.A. (2023). Modeling the structural, fractal and mechanical properties of aerogels. Springer Handbook of Aerogels.

[13] Aney S., Pandit P., Ratke L., Milow B., Rege A. (2025). On the origin of power-scaling exponents in silica aerogels. J. Sol.-Gel Sci. Technol..

[14] Voorhees P.W. (1985). The theory of Ostwald ripening. J. Stat. Phys..

[15] Li Y., Garing C., Benson S.M. (2020). A continuum-scale representation of Ostwald ripening in heterogeneous porous media. J. Fluid Mech..

[16] Rosowski K.A., Vidal-Henriquez E., Zwicker D., Style R.W., Dufresne E.R. (2020). Elastic stresses reverse Ostwald ripening. Soft Matter.

[17] Curk T., Luijten E. (2023). Phase separation and ripening in a viscoelastic gel. Proc. Natl. Acad. Sci. USA.

[18] Andreotti B., Snoeijer J.H. (2020). Statics and dynamics of soft wetting. Annu. Rev. Fluid Mech..

[19] Zhang X., Chen Y., Ma Y., Yin Q., Pan F., Cui C., Zhang Z., Liu B. (2021). Advances in mechanics of hierarchical composite materials. Prog. Mater. Sci..

[20] Han H., Kallakuri S., Yao Y., Williamson C.B., Nevers D.R., Savitzky B.H., Skye R.S., Xu M., Voznyy O., Dshemuchadse J. (2022). Multiscale hierarchical structures from a nanocluster mesophase. Nat. Mater..

[21] Liang R., Xue Y., Fu X., Le A.N., Song Q., Qiang Y., Xie Q., Dong R., Sun Z., Osuji C.O. (2022). Hierarchically engineered nanostructures from compositionally anisotropic molecular building blocks. Nat. Mater..

[22] Ferrando-Soria J., Fernández A. (2024). Integrating levels of hierarchical organization in porous organic molecular materials. Nano-Micro Lett..

[23] Greer J.R., Deshpande V.S. (2019). Three-dimensional architected materials and structures: Design, fabrication, and mechanical behavior. MRS Bull..

[24] Takeshita S., Mine S., Ono T. (2025). Data-driven review on aerogels and polymeric aerogels. Angew. Chem. Int. Ed..

[25] Qian X., Zhou J., Chen G. (2021). Phonon-engineered extreme thermal conductivity materials. Nat. Mater..

[26] Li Y., Li W., Han T., Zheng X., Li J., Li B., Fan S., Qiu C.-W. (2021). Transforming heat transfer with thermal metamaterials and devices. Nat. Rev. Mater..

[27] Xia X., Spadaccini C.M., Greer J.R. (2022). Responsive materials architected in space and time. Nat. Rev. Mater..

[28] Benedetti M., Plessis A., Ritchie R.O., Dallago M., Razavi N., Berto F. (2021). Architected cellular materials: A review on their mechanical properties towards fatigue-tolerant design. Mater. Sci. Eng. R Rep..

[29] Saccone M.A., Gallivan R.A., Narita K., Yee D.W., Greer J.R. (2022). Additive manufacturing of micro-architected metals via hydrogel infusion. Nature.

[30] Surjadi J.U., Portela C.M. (2025). Enabling three-dimensional architected materials across length scales and timescales. Nat. Mater..

[31] Su L., Niu M., Lu D., Cai Z., Li M., Wang H. (2021). A review on the emerging resilient and multifunctional ceramic aerogels. J. Mater. Sci. Technol..

[32] Parale V.G., Kim T., Choi H., Phadtare V.D., Dhavale R.P., Kanamori K., Park H.-H. (2024). Mechanically strengthened aerogels through multiscale, multicompositional, and multidimensional approaches: A review. Adv. Mater..

[33] Song S., Lu J., Zheng F., Duong H.M., Lu L. (2015). A facile strategy to achieve high conduction and excellent chemical stability of lithium solid electrolytes. RSC Adv..

[34] Wu L., Li Y., Fu Z., Su B.-L. (2020). Hierarchically structured porous materials: Synthesis strategies and applications in energy storage. Natl. Sci. Rev..

[35] Clayson I.G., Hewitt D., Hutereau M., Pope T., Slater B. (2020). High-throughput methods in the synthesis, characterization, and optimization of porous materials. Adv. Mater..

[36] Lyu J., Li L., Zhang X. (2025). Nanoporous aramid colloidal aerogels: Design, fabrication, and performance. Prog. Polym. Sci..

[37] Chen L., Yu X., Gao M., Xu C., Zhang J., Zhang X., Zhu M., Cheng Y. (2024). Renewable biomass-based aerogels: From structural design to functional regulation. Chem. Soc. Rev..

[38] Abdul Khalil H.P.S., Yahya E.B., Jummaat F., Adnan A.S., Olaiya N.G., Rizal S., Abdullah C.K., Pasquini D., Thomas S. (2023). Biopolymers-based aerogels: A review on revolutionary solutions for smart therapeutics delivery. Prog. Mater. Sci..

[39] Zhao G., Shi L., Yang G., Zhuang X., Cheng B. (2023). 3D fibrous aerogels from 1D polymer nanofibers for energy and environmental applications. J. Mater. Chem. A.

[40] Chen Y., Zhang L., Yang Y., Pang B., Xu W., Duan G., Jiang S., Zhang K. (2021). Recent progress on nanocellulose aerogels: Preparation, modification, composite fabrication, applications. Adv. Mater..

[41] Saha P., Ghosh S.K., Singh P. (2025). A holistic review on synthesis, properties, and multifunctional applications of cellulose aerogels from cellulose waste streams. Int. J. Biol. Macromol..

[42] Ma Y., Hu Y., Yang X., Shang Q., Huang Q., Hu L., Jia P., Zhou Y. (2025). Fabrication, functionalization and applications of cellulose-based aerogels: A review. Int. J. Biol. Macromol..

[43] Militký J., Venkataraman M., Sözcü Ş. (2026). Peculiarities of bacterial cellulose. Polymers.

[44] Panahi-Sarmad M., Alikarami N., Guo T., Haji M., Jiang F., Rojas O.J. (2024). Aerogels based on bacterial nanocellulose and their applications. Small.

[45] Zhou J., Luo Z., Kou Y., Zhou X., Li X., Zhang W., Deng Z. (2025). Long-chain nanocellulose-based aerogels for water treatment: Strategies from fabrication towards modification. Chem. Eng. J..

[46] Vo T.S., Chit P.P., Nguyen V.H., Hoang T., Lwin K.M., Vo T.T.B.C., Jeon B., Han S., Lee J., Park Y. (2024). A comprehensive review of chitosan-based functional materials: From history to specific applications. Int. J. Biol. Macromol..

[47] Friday C., Edgar K. (2026). Recent developments in all-polysaccharide hydrogels. Carbohydr. Polym..

[48] Zheng Q., Tian Y., Ye F., Zhou Y., Zhao G. (2020). Fabrication and application of starch-based aerogel: Technical strategies. Trends Food Sci. Technol..

[49] Grant G.T., Morris E.R., Rees D.A., Smith P.J.C., Thom D. (1973). Biological interactions between polysaccharides and divalent cations: The egg-box model. FEBS Lett..

[50] Guastaferro M., Reverchon E., Baldino L. (2021). Agarose, alginate and chitosan nanostructured aerogels for pharmaceutical applications: A short review. Front. Bioeng. Biotechnol..

[51] Coates G.W., Getzler Y.D.Y.L. (2020). Chemical recycling to monomer for an ideal circular polymer economy. Nat. Rev. Mater..

[52] Abraham E., Cherpak V., Senyuk B., ten Hove J.B., Lee T., Liu Q., Smalyukh I.I. (2023). Highly transparent silanized cellulose aerogels for boosting energy efficiency of glazing in buildings. Nat. Energy.

[53] Gurikov P., Smirnova I. (2018). Aerogel production: Current status, research directions, and future opportunities. J. Supercrit. Fluids.

[54] Cui S., Li R., Song T., Zhang H., Wang J. (2025). Protein-based aerogels: Preparation, formation mechanism, characterizations, and applications. J. Food Process Eng..

[55] Geyer R., Jambeck J.R., Law K.L. (2017). Production, use, and fate of all plastics ever made. Sci. Adv..

[56] Xiang Y., Yan M., Li L., Xiao Y., Sun H., Zhang Z., Yang H., Cheng X., Pan Y. (2025). Low thermal conductivity and self-cleaning silica aerogel coating based on a secondary coating encapsulation strategy. Constr. Build. Mater..

[57] Zhang T., Zhang R., Zhao C., Li Z., Wang L., Zhao H. (2025). Preparation, characterization, multidimensional applications and prospects of protein bio-based hydrogels: A review. Int. J. Biol. Macromol..

[58] Sun F., Zhang Y., Zhang B., Qiao D., Xie F. (2025). Fibrous protein gels: Nanoscale features governing gelation. Adv. Colloid Interface Sci..

[59] Lu Z., Hauschild M., Ottosen L.M., Ambaye T.G., Zerbino P., Aloini D., Lima A.T. (2024). Climate mitigation potential of bio-based insulation materials: A comprehensive review and categorization. J. Clean. Prod..

[60] Chen L., Yu L., Qi L., Eichhorn S.J., Isogai A., Lizundia E., Zhu J.Y., Chen C. (2025). Cellulose nanocomposites by supramolecular chemistry engineering. Nat. Rev. Mater..

[61] Feng J., Su B.-L., Xia H., Zhao S., Gao C., Wang L., Ogbeide O., Feng J., Hasan T. (2021). Printed aerogels: Chemistry, processing, and applications. Chem. Soc. Rev..

[62] Morris E.R., Rees D.A., Thom D., Boyd J. (1978). Chiroptical and stoichiometric evidence of a specific, primary dimerisation process in alginate gelation. Carbohydr. Res..

[63] Yan L., Huertas-Alonso A.J., Liu H., Dai L., Si C., Sipponen M.H. (2025). Lignin polymerization: Towards high performance materials. Chem. Soc. Rev..

[64] Huo L., Lu Y., Ding W.L., Wang Y., Li X., He H. (2025). Lignin-based hydrogels and adhesives: Preparation and properties. Green Chem..

[65] Wong S.S., Shu R., Zhang J., Liu H., Yan N. (2020). Downstream processing of lignin derived feedstock into end products. Chem. Soc. Rev..

[66] Xue T., Zhao X., Yang F., Tian J., Qin Y., Guo X., Fan W., Liu T. (2024). Fatigue-resistant and thermal insulating polyimide nanofibrous aerogels with temperature-invariant flexibility and nanofiber–lamella crosslinking architecture. J. Mater. Chem. A.

[67] Zong S., Feng C., Lei F., Zhu L., Jiang J., Duan J. (2025). Construction of Nanocellulose Aerogels with Environmental Drying Strategy without Organic Solvent Displacement for High-Efficiency Solar Steam Generation. ACS Nano.

[68] Jarms J., Borzęcka N.H., Serrador Goncalves B., Ganesan K., Milow B., Rege A. (2025). Modeling of the Gelation Process in Cellulose Aerogels. Biomacromolecules.

[69] Lei Z., Chen H., Luo C., Rong Y., Hu Y., Jin Y., Long R., Yu K., Zhang W. (2022). Recyclable and malleable thermosets enabled by activating dormant dynamic linkages. Nat. Chem..

[70] Zhao S., Siqueira G., Drdova S., Norris D., Ubert C., Bonnin A., Galmarini S., Ganobjak M., Pan Z., Brunner S. (2020). Additive manufacturing of silica aerogels. Nature.

[71] Gu X., Ling Y. (2024). Research progress of aerogel materials in the field of construction. Alex. Eng. J..

[72] Chen W., Zhou X., Wan M., Tang Y. (2022). Recent progress on polyimide aerogels against shrinkage: A review. J. Mater. Sci..

[73] Cascione V., Roberts M., Allen S., Charbel C., Maskell D., Dams B., Shea A., Walker P., Emmitt S. (2025). Evaluating environmental impacts of bio-based insulation materials through scenario-based and dynamic life cycle assessment. Int. J. Life Cycle Assess..

[74] Burroughs M.C., Schloemer T.H., Congreve D.N., Mai D.J. (2023). Gelation dynamics during photo-cross-linking of polymer nanocomposite hydrogels. ACS Polym. Au.

[75] Malkin A.Y., Derkach S.R. (2024). Gelation of polymer solutions as a rheological phenomenon. Curr. Opin. Colloid Interface Sci..

[76] Sun M., Felsenthal L.M., Kim S., Choi E.Y., Reed L.J., Elling B.R., Dichtel W.R. (2026). Covalent adaptable networks: Reprocessable cross-linked polymers. Chem. Rev..

[77] Qiao M. (2024). Closing the thermoset recycling loop. Nat. Chem. Eng..

[78] Zhan Y., Zhang C., Li L., Huang M., Chen S., Jiang Y., Feng J., Hu Y., Feng J. (2025). Phenolic aerogel modified by SiO_2_–ZrO_2_ for efficient thermal protection and insulation. Gels.

[79] Liu Y., Yu Z., Wang B., Li P., Zhu J., Ma S. (2022). Closed-loop chemical recycling of thermosetting polymers and their applications: A review. Green Chem..

[80] Liu Z., Liu L., Li Y., Yang K., Li S., Li W., Zhu Y., Xie T. (2025). Atom economic closed-loop recycling of thermoset polyurethane foams. Nat. Commun..

[81] Zhang X., Kwek L.P., Le D.K., Tan M.S., Duong H.M. (2019). Fabrication and Properties of Hybrid Coffee-Cellulose Aerogels from Spent Coffee Grounds. Polymers.

[82] Han M., Ren L., Zhao B., Huang Z., Liu Z., Predicala B., Ma Y., Guo L. (2025). Sustainable and mechanically robust bio-based aerogels for multifunctional air filtration. J. Clean. Prod..

[83] Cen Q., Chen S., Yang D., Zheng D., Qiu X. (2023). Full bio-based lignin aerogel for flame retardancy and insulation. ACS Sustain. Chem. Eng..

[84] Zhao J., Wang J., Liu Z., Zhang Y., Mi Y. (2023). Preparation of porous epoxy resin materials by reaction-induced phase separation regulated using fumed silica. ACS Appl. Polym. Mater..

[85] Yang X., Miao W., Sun X., Pan Y.-T. (2025). Recent advances in sustainable biomass-based aerogels: A review. RSC Sustain..

[86] Zhu C.Y., Xu H.B., Zhao X.P., Gong L., Li Z.Y. (2022). A review on heat transfer in nanoporous silica aerogel insulation materials and its modeling. Energy Storage Sav..

[87] Zeng M., Xu Q., Yu J. (2025). Polyvinyltrimethoxysilane-enhanced TEMPO-oxidized cellulose nanofiber aerogels for exceptional anisotropic thermal insulation, flame retardancy and oil/water separation. Ind. Crops Prod..

[88] Wang Y., Xi S., Zhou B., Zu G., Liang X., Zhang X., Shen J., Wang X. (2024). Superhydrophobic highly flexible triple-network polyorganosiloxane-based aerogels for thermal insulation, oil–water separation, and strain/pressure sensing. ACS Appl. Mater. Interfaces.

[89] Zu G., Kanamori K., Nakanishi K., Huang J. (2019). Superhydrophobic ultraflexible triple-network graphene/polyorganosiloxane aerogels for a high-performance multifunctional temperature/strain/pressure sensing array. Chem. Mater..

[90] Nguyen H.S.H., Huynh H.K.P., Nguyen S.T., Nguyen V.T.T., Nguyen T.-A., Phan A.N. (2022). Insights into sustainable aerogels from lignocellulosic materials. J. Mater. Chem. A.

[91] García-González C.A., Blanco-Vales M., Barros J., Boccia A.C., Budtova T., Durães L., Erkey C., Gallo M., Herman P., Kalmár J. (2025). Review and perspectives on the sustainability of organic aerogels. ACS Sustain. Chem. Eng..

[92] Wang C., Eisenreich F., Tomović Ž. (2023). Closed-loop recyclable high-performance polyimine aerogels derived from bio-based resources. Adv. Mater..

[93] Shahriar S.M.S., McCarthy A.D., Andrabi S.M., Su Y., Polavoram N.S., John J.V., Matis M.P., Zhu W., Xie J. (2024). Mechanically resilient hybrid aerogels containing fibers of dual-scale sizes and knotty networks for tissue regeneration. Nat. Commun..

[94] Turhan Kara I., Kiyak B., Colak Gunes N., Yucel S. (2024). Life cycle assessment of aerogels: A critical review. J. Sol.-Gel Sci. Technol..

[95] Wu C., Huang H., Jin X., Yan X., Wang H., Pan Y., Zhang X., Hong C. (2023). Water-assisted synthesis of phenolic aerogel with superior compression and thermal insulation performance enabled by thick-united nanostructure. Chem. Eng. J..

[96] Hayashi M., Ricarte R.G. (2025). Towards the next development of vitrimers: Recent key topics for the practical application and understanding of the fundamental physics. Prog. Polym. Sci..

[97] Meng F., Saed M.O., Terentjev E.M. (2022). Rheology of vitrimers. Nat. Commun..

[98] Koh H.W., Le D.K., Ng G.N., Zhang X., Phan-Thien N., Kureemun U., Duong H.M. (2018). Advanced recycled polyethylene terephthalate aerogels from plastic waste for acoustic and thermal insulation applications. Gels.

[99] Goh X.Y., Guo K., Nguyen L.T., Ong R.H., Duong H.M. (2023). Fabrication and properties of polyethylene terephthalate (PET) aerogel composites from plastic bottle waste. Mater. Today Commun..

[100] Nguyen L.T., Bai T., Lu Z.B., Woo N.Y.R., Goh X.Y., Tran A.D., Tran C.-D., Duong H.M. (2025). Upcycling polyethylene terephthalate (PET) plastic waste into multifunctional aerogel-inspired materials. Constr. Build. Mater..

[101] Yuan C., Wang D., Zhang Y., Li K., Ding J. (2023). Research progress on preparation, modification, and application of phenolic aerogel. Nanotechnol. Rev..

[102] Guo H., Meador M.A.B., Cashman J.L., Tresp D., Dosa B., Scheiman D.A., McCorkle L.S. (2020). Flexible polyimide aerogels with dodecane links in the backbone structure. ACS Appl. Mater. Interfaces.

[103] Chen Z., Chen J., Hou X., Chen L. (2024). Mechanically robust polyimide aerogel films by polymerization-regulated strategy for flexible thermal protection. Chem. Eng. J..

[104] Nguyen B.N., Scheiman D.A., Meador M.A.B., Guo J., Hamilton B.R., McCorkle L.S. (2021). Effect of urea links in the backbone of polyimide aerogels. ACS Appl. Polym. Mater..

[105] Li M., Wu Z., Chen X., Gan F., Teng C., Li X., Dong J., Zhao X., Zhang Q. (2024). Continuous and strong polyimide aerogel fibers enhanced by para-aramid nanofibers prepared via a reaction spinning method for thermal insulation. Chem. Eng. J..

[106] Goh X.Y., Deng X., Teo W.S., Ong R.H., Nguyen L.T., Bai T., Duong H.M. (2024). Scale-up fabrication and advanced properties of recycled polyethylene terephthalate aerogels from plastic waste. Polym. Eng. Sci..

[107] Goh X.Y., Hwang J., Bai T., Ong R.H., Nguyen L.T., Duong H.M. (2024). Sub-ambient radiative cooling with thermally insulating polyethylene terephthalate aerogels recycled from plastic waste. Sol. Energy.

[108] Nguyen L.T., Goh C.J., Bai T., Ong R.H., Goh X.Y., Duong H.M. (2024). Scalable fabrication of lightweight carbon nanotube aerogel composites for full X-band electromagnetic wave absorption. Carbon.

[109] Phan Q.M.N., Duc T., Nguyen P.T.T., Zheng Y., Jin W., Nguyen L.T., Wang M., Do N.H.N., Le P.K., Duong H.M. (2026). Sustainable end-of-life strategies for thermoset-based fiber-reinforced composites: Advances in recycling and upcycling. J. Phys. Mater..

[110] Krishnakumar B., Sanka R.V.S.P., Binder W.H., Parthasarthy V., Rana S., Karak N. (2020). Vitrimers: Associative dynamic covalent adaptive networks in thermoset polymers. Chem. Eng. J..

[111] Wang C., Eisenreich F., Tomović Ž. (2024). Aerogel-to-sol-to-aerogel (ASA) process for recycling, repairing, and reprogramming organic aerogels. Adv. Funct. Mater..

[112] Zhao X., Chen X., Yuk H., Lin S., Liu X., Parada G. (2021). Soft materials by design: Unconventional polymer networks give extreme properties. Chem. Rev..

[113] Sha R., Cheng X., Dai J., Zu Y., Zeng Y., Sha J. (2022). Lightweight phenolic resin aerogel with excellent thermal insulation and mechanical properties via an ultralow shrinkage process. Mater. Lett..

[114] Gao W., Wang Z., Zhang Y., Shen C., Wang Y., Zhan L. (2024). High-energy B–O bonds enable the phenolic aerogel with enhanced thermal stability and low thermal conductivity. Appl. Surf. Sci..

[115] Wang L., Li W., Wang C., Jiang Y., Liu H., Pang T., He X., Li M., He F. (2024). Cocktail-like double-layered polyimide/alumina composite aerogels via side-direction freezing method as designable high-temperature thermal insulations. Chem. Eng. J..

[116] Li C., Chen Z., Dong W., Lin L., Zhu X., Liu Q., Zhang Y., Zhai N., Zhou Z., Wang Y. (2021). Silicon-based aerogel thermal insulation materials: Performance optimization through composition and microstructure. J. Non-Cryst. Solids.

[117] Zhao S., Malfait W.J., Guerrero-Alburquerque N., Koebel M.M., Nyström G. (2018). Biopolymer Aerogels and Foams: Chemistry, Properties, and Applications. Angew. Chem. Int. Ed..

[118] Fu X., Si L., Zhang Z., Yang T., Feng Q., Song J., Zhu S., Ye D. (2025). Gradient all-nanostructured aerogel fibers for enhanced thermal insulation and mechanical properties. Nat. Commun..

[119] Wang C.-L., Chen Y.-R., Mezari B., Eisenreich F., Tomović Ž. (2025). Advancing aerogel recyclability through polyhexahydrotriazine reactivity. Nat. Commun..

[120] Li J., Ali A.B.M., Babadoust S., Salahshour S., Sabetvand R., Brahmia A. (2025). Molecular dynamics simulation of reinforced silica aerogel with a phase change material at different initial pressures. Int. Commun. Heat Mass Transf..

[121] Abdusalamov R., Scherdel C., Itskov M., Milow B., Reichenauer G., Rege A. (2021). Modeling and Simulation of the Aggregation and the Structural and Mechanical Properties of Silica Aerogels. J. Phys. Chem. B.

[122] Hamelin G., Jauffrés D., Martin C.L., Meille S., Foray G. (2021). Mechanical properties of milimetric silica aerogel particles produced through evaporative drying: A coupled experimental and discrete element approach. J. Non-Cryst. Solids.

[123] Aniszewska D., Rybaczuk M. (2021). Mechanical properties of silica aerogels modelled by Movable Cellular Automata simulations. Mater. Today Commun..

[124] Aney S., Rege A. (2025). Network decomposition model to describe the solid and gaseous thermal conductivity in open-porous (nano)materials. Int. J. Heat Mass Transf..

[125] Zhu W., Kan A., Chen Z., Zhang Q., Zhang J. (2022). A modified Lattice Boltzmann method for predicting the effective thermal conductivity of open-cell foam materials. Int. Commun. Heat Mass Transf..

[126] Xu X., Huang Q., Chen B., Niu B., Zhang Y., Long D. (2024). High-Precision 3D reconstruction and quantitative structure description: Linking microstructure to macroscopic heat transfer of aerogels. Chem. Eng. J..

[127] Li X., Zhou S., Liu X., Zang J., Fu W., Lu W., Zhang H., Yan Z. (2024). 3D microstructure reconstruction and characterization of porous materials using a cross-sectional SEM image and deep learning. Heliyon.

[128] Liu D., Chen H., Chacon L.A., Ramu V.M., Poovathingal S.J. (2024). Micro-CT image-based computation of effective thermal and mechanical properties of fibrous porous materials. Compos. Part B Eng..

[129] Liu D., Maya K.A., Chen H., Banerjee A., Chacon L.A., Poovathingal S.J. (2025). The effective thermal and elastic properties of FiberForm: Computation, microstructure-sensitivity analysis and epistemic uncertainty quantification. Comput. Mater. Sci..

[130] Walker R.C., Hyer A.P., Guo H., Ferri J.K. (2023). Silica Aerogel Synthesis/Process-Property Predictions by Machine Learning. Chem. Mater..

[131] Tafreshi O.A., Saadatnia Z., Ghaffari-Mosanenzadeh S., Okhovatian S., Park C.B., Naguib H.E. (2023). Machine learning-based model for predicting the material properties of nanostructured aerogels. SPE Polym..

[132] Rong C., Zhou L., Zhang B., Xuan F.-Z. (2023). Machine learning for mechanics prediction of 2D MXene-based aerogels. Compos. Commun..

[133] Lu L., Bai A., Wang H., Xi W., Yun S., Gao S., Wang M., Sun X. (2025). Pareto-driven gradient-boosted Bayesian optimization for multi-objective inverse design of aerogel-cement-EPS panels in small-sample regimes. Case Stud. Constr. Mater..

[134] Khatamsaz D., Vela B., Singh P., Johnson D.D., Allaire D., Arróyave R. (2022). Multi-objective materials Bayesian optimization with active learning of design constraints: Design of ductile refractory multi-principal-element alloys. Acta Mater..

[135] Khatamsaz D., Vela B., Singh P., Johnson D.D., Allaire D., Arróyave R. (2023). Bayesian optimization with active learning of design constraints using an entropy-based approach. npj Comput. Mater..

[136] Swain M.C., Cole J.M. (2016). ChemDataExtractor: A toolkit for automated extraction of chemical information from the scientific literature. J. Chem. Inf. Model..

[137] Trewartha A., Walker N., Huo H., Lee S., Cruse K., Dagdelen J., Dunn A., Persson K.A., Ceder G., Jain A. (2022). Quantifying the advantage of domain-specific pre-training on named entity recognition tasks in materials science. Patterns.

[138] Gupta T., Zaki M., Krishnan N.M.A., Mausam (2022). MatSciBERT: A materials domain language model for text mining and information extraction. npj Comput. Mater..

[139] Shetty P., Rajan A.C., Kuenneth C., Gupta S., Panchumarti L.P., Holm L., Zhang C., Ramprasad R. (2023). A general-purpose material property data extraction pipeline from large polymer corpora using natural language processing. npj Comput. Mater..

[140] Polak M.P., Morgan D. (2024). Extracting accurate materials data from research papers with conversational language models and prompt engineering. Nat. Commun..

[141] Mishra V., Singh S., Zaki M., Ahlawat D., Grover H., Mishra B., Miret S., Krishnan N.M.A. (2024). Foundational large language models for materials research. arXiv.

[142] Park J., Gill A.P.S., Moosavi S.M., Kim J. (2024). Inverse design of porous materials: A diffusion model approach. J. Mater. Chem. A.

[143] Yang K., Schwalbe-Koda D. (2025). A generative diffusion model for amorphous materials. npj Comput. Mater..

[144] Zeni C., Pinsler R., Zügner D., Fowler A., Horton M., Fu X., Wang Z., Shysheya A., Crabbé J., Ueda S. (2025). A generative model for inorganic materials design. Nature.

[145] Szymanski N.J., Rendy B., Fei Y., Kumar R.E., He T., Milsted D., McDermott M.J., Gallant M., Cubuk E.D., Merchant A. (2023). An autonomous laboratory for the accelerated synthesis of inorganic materials. Nature.

[146] Burger B., Maffettone P.M., Gusev V.V., Aitchison C.M., Bai Y., Wang X., Li X., Alston B.M., Li B., Clowes R. (2020). A mobile robotic chemist. Nature.

[147] Attia P.M., Grover A., Jin N., Severson K.A., Markov T.M., Liao Y.-H., Chen M.H., Cheong B., Perkins N., Yang Z. (2020). Closed-loop optimization of fast-charging protocols for batteries with machine learning. Nature.

[148] Zhang Y., Ling C. (2018). A strategy to apply machine learning to small datasets in materials science. npj Comput. Mater..

[149] Vishnyakov A. (2025). Machine learning in computational design and optimization of disordered nanoporous materials. Materials.

[150] Jain A., Ong S.P., Hautier G., Chen W., Richards W.D., Dacek S., Cholia S., Gunter D., Skinner D., Ceder G. (2013). Commentary: The Materials Project: A materials genome approach to accelerating materials innovation. APL Mater..

[151] Curtarolo S., Setyawan W., Hart G.L.W., Jahnatek M., Chepulskii R.V., Taylor R.H., Wang S., Xue J., Yang K., Levy O. (2012). AFLOW: An automatic framework for high-throughput materials discovery. Comput. Mater. Sci..

[152] Lin P., Qing X., Liu Q., Yang Y. (2025). Flexible aerogels for thermal insulation: Fabrication and application. ACS Appl. Mater. Interfaces.

[153] Zhao T., Chen Z., Xiao W., Zhou Y., Zhan B., Lyu Y., Li S., Liu Y. (2025). Cellulose aerogels in water pollution treatment: Preparation, applications and mechanism. Adv. Bionics.

[154] Wang X., Chen Y., Lindbråthen A., Waris Z., Deng L. (2025). High capacity and robust moisture-swing CO^2^ adsorption for direct air capture by functionalized cellulose aerogels. Chem. Eng. J..

[155] Carter M., Nguyen H.G.T., Allen A.J., Yi F., Yang W.-C.D., Baumann A.E., McGivern W.S., Manion J.A., Kuzmenko I., Tsinas Z. (2024). Progress in development of characterization capabilities to evaluate candidate materials for direct air capture applications. J. CO2 Util..

[156] Li L., Jin G., Shen J., Guo M., Song J., Li Y., Xiong J. (2025). Carbon aerogels: Synthesis, modification, and multifunctional applications. Gels.

[157] Jeong Y., Patel R., Patel M. (2024). Biopolymer-based biomimetic aerogel for biomedical applications. Biomimetics.

[158] Liu Y., Zheng S., Yang Y., Han M., Zeng Z., Wu N. (2026). Advanced aerogels for high-efficiency electromagnetic wave shielding and absorption. Adv. Funct. Mater..

[159] Wang W., Wang S., Li D., Yang J., Li H. (2025). Tailoring polyimide aerogel performance: The role of solid content in influencing microstructure, mechanical, thermal, and acoustic behaviors. RSC Adv..

[160] Sp T.S., Nguyen P.T.T., Do N.H.N., Le D.K., Thai Q.B., Le P.K., Phan-Thien N., Duong H.M. (2021). Advanced fabrication and multi-properties of aluminium hydroxide aerogels from aluminium wastes. J. Mater. Cycles Waste Manag..

[161] Thai Q.B., Le-Cao K., Nguyen P.T., Le P.K., Phan-Thien N., Duong H.M. (2021). Fabrication and optimization of multifunctional nanoporous aerogels using recycled textile fibers from car tire wastes for oil-spill cleaning, heat-insulating and sound absorbing applications. Colloids Surf. A Physicochem. Eng. Asp..

[162] Loh J.W., Goh X.Y., Nguyen P.T.T., Thai Q.B., Ong Z.Y., Duong H.M. (2022). Advanced aerogels from wool waste fibers for oil spill cleaning applications. J. Polym. Environ..

[163] (2006). Environmental Management—Life Cycle Assessment—Requirements and Guidelines.

[164] Zhang X., Zhao J., Liu K., Li G., Zhao D., Zhang Z., Wan J., Yang X., Bai R., Wang Y. (2022). Weldable and closed-loop recyclable monolithic dynamic covalent polymer aerogels. Natl. Sci. Rev..

[165] Thommes M., Kaneko K., Neimark A.V., Olivier J.P., Rodriguez-Reinoso F., Rouquerol J., Sing K.S.W. (2015). Physisorption of gases, with special reference to the evaluation of surface area and pore size distribution (IUPAC Technical Report). Pure Appl. Chem..

